# A Novel Collection of snRNA-Like Promoters with Tissue-Specific Transcription Properties

**DOI:** 10.3390/ijms130911323

**Published:** 2012-09-11

**Authors:** Sonia Garritano, Arianna Gigoni, Delfina Costa, Paolo Malatesta, Tullio Florio, Ranieri Cancedda, Aldo Pagano

**Affiliations:** 1 Department of Experimental Medicine (DiMES), University of Genoa, 16132 Genoa, Italy; E-Mails: sgarritano@biologia.unipi.it (S.G.); arianna.gigoni@hotmail.it (A.G.); delfinacosta@gmail.com (D.C.); paolo.malatesta@unige.it (P.M.); ranieri.cancedda@unige.it (R.C.); 2 IRCCS-AOU San Martino-IST, Largo Rosanna Benzi 10, 16132 Genoa, Italy; 3 Department of Internal Medicine (DIMI), University of Genoa, 16132 Genoa, Italy; E-Mail: tullio.florio@unige.it; 4 Centre of Excellence for Biomedical research (CEBR), University of Genoa, 16132 Genoa, Italy

**Keywords:** RNA polymerase III, alternative splicing, non-coding RNA, small nuclear RNA

## Abstract

We recently identified a novel dataset of snRNA-like trascriptional units in the human genome. The investigation of a subset of these elements showed that they play relevant roles in physiology and/or pathology. In this work we expand our collection of small RNAs taking advantage of a newly developed algorithm able to identify genome sequence stretches with RNA polymerase (pol) III type 3 promoter features thus constituting putative pol III binding sites. The bioinformatic analysis of a subset of these elements that map in introns of protein-coding genes in antisense configuration suggest their association with alternative splicing, similarly to other recently characterized small RNAs. Interestingly, the analysis of the transcriptional activity of these novel promoters shows that they are active in a cell-type specific manner, in accordance with the emerging body of evidence of a tissue/cell-specific activity of pol III.

## 1. Introduction

RNA polymerase (pol) machineries represent one of the most illustrative differences between prokaryotes and eukaryotes. Indeed, prokaryotes are endowed with a single RNA polymerase able to synthesize all RNA species, whereas eukaryotes are equipped with three different enzymes (pol I, II and III) devoted to the synthesis of different classes of RNA molecules [1]. Among eukaryotic transcriptional machineries, pol III has been associated with the expression of structural RNAs ubiquitously expressed such as 5S rRNA, tRNAs and a few small nuclear (sn) RNAs. However, although the synthesis of 5S rRNA and tRNAs is regulated by the level of activity of the pol III machinery [2], experimental evidence of a gene-specific regulation has recently emerged [3]. In detail, in a previous work [4] we proposed for the first time that the human genome might host a very large number of snRNA-like transcriptional units able to regulate, in a tissue/stage-specific manner, the expression of a set of pol II-transcribed protein-coding counterparts [1,4]. Notably, studying their function we found that pol III-transcribed RNAs play relevant regulatory roles in pathways involved in pathological processes such as cancer and/or neurodegeneration [5–11].

Therefore, it was strongly suggested that possible novel nucleic acid molecules, responsible for the regulation of poorly known physiopathological processes, might be found while searching for novel pol III type 3 transcriptional units.

Based on this hypothesis, in the present work we expand our database by a computational approach searching for human genomic portions in which the simultaneous occurrence of the different regulatory elements of the pol III type 3 transcriptional unit make them appropriate for pol III transcription. To this aim we took advantage of a newly developed algorithm that allows considering simultaneously different regulatory elements of putative transcriptional units. We provide a collection of 3909 novel putative pol III type 3 transcription units, together with detailed bioinformatic analysis of their characteristics that, interestingly, suggest the association with an alternative splicing process, in agreement with our recent results [6,7]. We also show the tissue/cell type-specific regulation of transcription of a restricted number of these RNAs used as experimental models. Altogether, our results support a cell type-specific regulation of pol III transcription reinforcing the view of a large number of pol III-transcribed small RNAs in the human genome with possible regulatory roles and point toward the detailed study of their function in order to open novel ways of investigation of still unresolved pathologies.

## 2. Results and Discussion

### 2.1. RNA Polymerase (pol) III Transcripts Are Widely Spread throughout the Human Genome

In order to identify the highest number of putative pol III transcription units possibly accounting for the synthesis of small nuclear (sn)RNA-like transcripts we took advantage of COMPASS, a public software that identifies the simultaneous occurrence of some superimposed conditions in a relevant number of sequences [12]. The conditions chosen defined a canonical pol III type 3 promoter with a PSE (Proximal Sequence Element) consensus sequence, a TATA box and a transcription termination signal at appropriate distances from one another (Table 1). Using these conditions, we identified 3909 putative transcription units (see supplementary data S1) of which a subset of 1473 (37.68%) were maps within a protein-coding gene portion. The analysis of these sequences shows that the averaged PSE/TATA box distance is 34.19 bp whereas the PSE/PolyT distance is 407.8 bp. Since, according to the literature [1], PSE maps 50 bp upstream from the transcription start site, the average size of the transcript would be 358 nucleotides, a size compatible with that of canonical pol III type 3 ncRNA. Although no structural features that might make the possible transcription of the extragenic putative units unlikely were identified, we focused our analysis on the intragenic transcription units due to our interest in their hypothetic role in the regulation of protein-coding gene expression.

In order to assess whether the putative transcription units map preferentially in introns or rather in exons, their distribution was compared to a theoretical one based on the known structure of the human genome using the χ^2^ test [13]. We found that the number of putative transcripts mapping in introns was significantly higher than expected (*p* = 0.00878), which is in accordance with previous results showing that the introns were enriched in ncRNAs, which mildly regulate gene expression [14].

Next, we analyzed the orientation of the putative ncRNAs inside each gene, assuming the number of elements in 5′-3′ to be statistically equivalent to that in 3′-5′. Interestingly, we found that the vast majority of the transcripts are in 3′-5′ orientation with respect to the coding gene in which they map (*p* = 0.00664). Therefore, altogether these data indicate that the novel putative transcriptional units identified in the human genome map preferentially within intronic portions of pol II coding genes in an antisense configuration.

### 2.2. Analysis of the Gene List

In order to predict *in silico* the possible function of the novel putative transcripts, we took advantage of the David algorithm [15] using all the human genes as background to perform the comparisons. We used the “Functional Annotation Chart” tool to identify the most representative functions among these protein-coding genes.

We found that the most reported function is “alternative splicing”, as 646 of 1075 (60.1%) of the genes belong to this category (Table 2). Since the *p*-value resulting from the modified Fisher exact test is 7.70 × 10^−48^ this test suggests that this function is enriched in our group of transcriptional units. Next, analyzing the false discovery rate of our findings with the Benjamini test, we obtained a *p*-value of 3.90 × 10^−45^ supporting a functional association between protein-coding genes involved in alternative splicing and our collection of transcriptional units.

This result is particularly intriguing in light of our recent works showing that antisense RNAs mapping in introns of protein-coding genes can regulate their splicing pattern in different biological contexts [6,7,14]. It is tantalizing to postulate that putative ncRNAs of our collection mapping in introns of protein-coding genes constitute a new class of splicing regulators across the board, capable of regulating the splicing of splicing regulators. In addition, the “functional annotation clustering” tool of the same algorithm showed that genes grouped into Cluster 1 (enrichment score: 9.19) play a role in the synapse and in the cell junction (Table 3), whereas the “tissue expression” tool showed that their expression is preferentially associated with the hippocampus, epithelium and amygdala (Table 4).

Altogether these results suggest that the function of these novel ncRNAs might be associated with the regulation of expression of protein-coding genes involved in alternative splicing and that they play a role in the central nervous system. In agreement with these results, recent studies show that ncRNAs are indeed enriched in the central nervous system and that their coordinated expression might amplify brain complexity [16]. In addition, several recent reports document a deregulation of ncRNAs in different human neuropathologies such as Alzheimer’s disease, Parkinson’s disease and Fragile X mental retardation, suggesting a possible unprecedented relevance of these small RNAs in physiopathology [8,16–20].

### 2.3. *In vitro* Promoter Activity Assay

**Figure 1:**
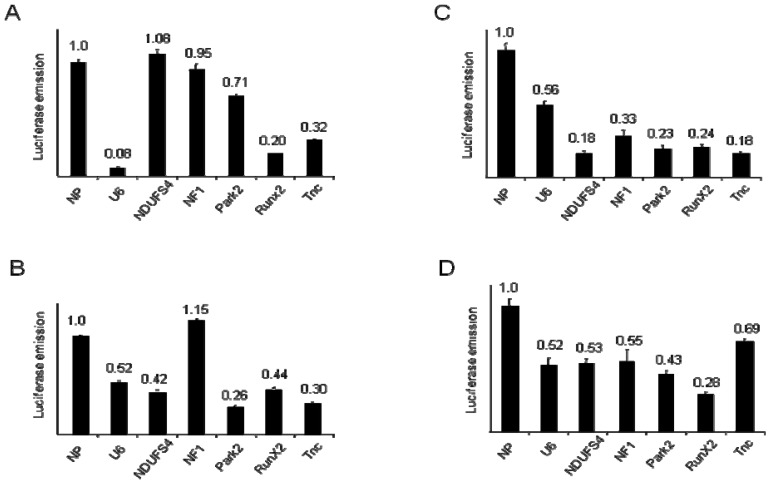
Promoter activity transfection assay of five novel pol III type 3 promoters in cells of different origin: (**A**) NCTC (Murine Liver); (**B**) SH-SY5Y (Human Neuroblastoma); (**C**) HeLa (Human Cervical Cancer) and; (**D**) U2OS (Osteosarcoma) cell lines. NP, pShag-No promoter negative control; U6, pShag-U6 positive control; NDUFS4, pShag-NDUFS4; NF1, pShag-NF1; Park2, pShag-Park2; Runx2, pShag-Runx2; Tnc, pShag-Tnc. Results are reported as the fraction of luciferase emission detected in cells transfected with pShag driven by different pol III type 3 promoters (U6; NDUFS4; NF1; Park2; Runx2; Tnc) with respect to the no promoter control (NP). Data are reported as mean values ± standard deviation resulting of three determinations.

In order to assess whether the predicted novel putative transcription units are indeed transcribed, we selected five elements, mapped in five protein-coding genes of interest (NF1, Neurofibromin 1 NM_001042492; Park2, parkin NM_004562; TNC, Tenascin C NM_002160; Runx2, Runt-related transcription factor 2 NM_004348; NDUFS4, NADH dehydrogenase Fe-S protein 4 NM_002495) as experimental models to test the transcriptional activity of their pol III type 3 promoters *in vitro*, in different cell lines. To detect quantitatively the transcription rate of the novel elements we fused their pol III type 3 promoters to a luciferase silencer hairpin (pSHAG-NF1/-Park2/-TNC/-Runx2/- NDUFS4, hereafter referred to as pS-NF1/-Park2/-TNC/-Runx2/-NDUFS4). In this condition, if the promoter is active in a specific cell line, the transcription of the hairpin drives the post-transcriptional silencing of a co-transfected luciferase cDNA and, ultimately, leads to the decrease of luciferase signal; on the contrary, an unaltered luminescent signal would indicate that the luciferase is not silenced and thus the promoter of the specific ncRNA is not actively transcribed in the cell line tested. Although a different experimental approach such as *in vitro* transcription and/or primer extension is needed to unambiguously validate the transcriptional activity of these putative promoters, as shown in Figure 1, they affect luciferase activity in a cell type-specific manner, suggesting specific biological roles associated with their corresponding protein-coding genes. Accordingly, the Runx2-associated ncRNA is actively transcribed in Murine Liver NCTC and in osteosarcoma Osteosarcoma U2OS cell lines (respectively 80% and 72% decrease of luciferase emission in cells transfected with pShag-Runx2), in which the Runx2 protein-coding gene (that encodes a nuclear protein essential for osteoblastic differentiation and skeletal morphogenesis) is expressed at high levels. Similarly, in neuroblastoma SH-SY5Y cells we detected the highest expression level of the park2-associated ncRNA (77% decrease of luciferase emission in cells SH-SY5Y transfected with pShag-Park2) that maps in the Park2 protein (parkin, NM_004562) [20].

## 3. Experimental Section

### 3.1. Identification of Polymerase III Promoters

The COMPASS (Complex Patterns Search Software, v.1.0.1.1.) software, which allows the use of degenerate sequences [12], was used to search the whole human genome for new possible non coding RNAs transcribed by the pol III promoter. The software searches a motif consisting of sequence and spacer in the whole sequence. As a motif we used the sequence of the pol III type 3 promoter, the PSE (proximal sequence element) and the TATA box, and the poly-T end as a termination signal. The consensus sequence used for the PSE was TYACCNTAAC, for the TATA-Box it was TATA, and for the termination signal a run of four Ts. The spacer between the PSE and TATA-Box was 35 ± 25 bp and the spacer between the TATA box and the poly T was 350 ± 200 bp (Table 1). This distance was chosen because the known transcripts made by pol III have a range from 69 bp (tRNAs, 70–90 bp) to a maximum of 400 bp (SINEs). A hit is only found if all three elements are found in the right distance to each other. To increase the velocity of the search COMPASS searches not only in 5′-3′ direction but also in 3′-5′ direction, converting the search string into 3′-5′. The FastA sequences of the human genome were taken from the University of California, Santa Cruz Genome Browser website [21].

### 3.2. Analysis of the Gene List-David Bioinformatics Resources

Putative pol III type 3 promoters identified by Compass software lay inside a gene sequence (intron, exon or spanning both). We analyzed this gene list using the DAVID algorithm [15] to extract different biological features and highlight the most over-represented biological annotation of all the genes. We used the “functional annotation card” tool to identify the most representative functions. As a background for the comparison we used all the human genome genes, and, if a biological process was enriched in our group, the *p*-value of the modified Fisher exact test would be lower than the cut off (0.05). To test the false discovery rate of the findings the Benjamini correction was applied.

The “functional annotation clustering” tool was used to group the similar annotation terms into clusters, and a high enrichment score indicates that the annotation term members in the groups play more important roles. The enrichment score is the geometric mean of all enrichment *p* values of each annotation term in the group. The minus log transformation is applied to this value so more attention should be given to groups with scores >1.3.

Finally, we used the tool “tissue-expression” to discover whether the genes that lie within these new putative pol III transcription units, are preferably expressed in specific tissues.

### 3.3. Cell Cultures

HeLa, U2OS and SH5YSY cells were maintained on DMEM medium as described elsewhere [22]. NCTC cells were maintained on MEM medium, 10% FBS and 2 mM L-Glutamine (EuroClone). Subconfluent cells were transfected with Effectene (Quiagen) according to the manufacturer’s instructions.

### 3.4. *In Vitro* Promoter Activity Assay

We selected five new putative pol III type 3 promoters lying inside intronic sequences to investigate their *in vitro* activity. In Table 2 these genes and the specific introns are reported. To characterize their activity, transient luciferase reporter assays were carried out. Briefly, each promoter sequence (sequence in Supplementary Material S2) was cloned in a pShag plasmid, upstream of a luciferase silencer hairpin. The promoter that activates the hairpin transcription will lead to an inhibition of the luciferase. On the contrary, an unaltered luciferase activity indicates that the putative promoter is not active. As positive and negative controls, a construct with a well-assessed type 3 promoter (U6, component of U6 small nuclear ribonucleoprotein) and a promoterless hairpin, respectively, were used. All cell lines used in this work were co-transfected with two plasmids: pShag, containing the promoter sequence, and pEGFP-N1 plasmid encoding for GFP gene. The experiment was performed in a 96-well plate, and 48 h after the transfection the medium was replaced with 150 μL of PBS to read the fluorescence (emission 535 nm, excitation 485 nm). Then the PBS was replaced with 150 μL of solution of medium and luciferine. The luminescence was read (integration time 1500 ms) and was normalized to the fluorescence as internal control of transfection efficiency. The readings were performed with the Genius pro instrument (TECAN).

## 4. Conclusions

In previous studies, we provided proof of evidence that novel, previously unknown pol III-transcribed ncRNAs play key roles in the maturation of specific gene products [6,7]. In light of this concept, and postulating a widespread distribution of this class of regulatory molecules in the human genome, we here analyzed the whole human genome taking advantage of a specific software able to identify sequences, putatively constituting a pol III type 3 promoter.

This search brought to light a large set of novel putative transcription units that preferentially map in introns of protein-coding genes possibly subjected to alternative splicing. The experimental analysis of five transcription units in four different cell lines revealed differential promoter activities suggesting widespread functions of the pol III transcript in diverse tissues.

## Supplementary Materials



## Figures and Tables

**Table 1:** Conditions used to identify the novel pol III type 3 promoter collection. Y, pYrimidine; N, aNy base.

PSE sequence	PSE/TATA spacer	TATA box	Transcribed portion length	Termination signal sequence
TYACCNTAAC	35 ± 25	TATA	350 ± 200	TTTT

**Table 2:** The Functional Annotation Chart tool identifies the most representative functions among the protein coding genes.

Category	Term	Count	%	*p*-Value	Benjamini
SP_PIR_KEYWORDS	alternative splicing	646	60.1	7.70 × 10^−48^	3.90 × 10^−45^
SP_PIR_KEYWORDS	phosphoprotein	548	51	2.40 × 10^−20^	6.10 × 10^−18^
SP_PIR_KEYWORDS	coiled coil	191	17.8	1.10 × 10^−13^	1.80 × 10^−11^
SP_PIR_KEYWORDS	cell junction	59	5.5	1.80 × 10^−11^	2.30 × 10^−9^
SP_PIR_KEYWORDS	synapse	39	3.6	1.60 × 10^−10^	1.60 × 10^−8^
SP_PIR_KEYWORDS	polymorphism	730	67.9	1.40 × 10^−8^	1.20 × 10^−6^
SP_PIR_KEYWORDS	ionic channel	45	4.2	2.30 × 10^−8^	1.60 × 10^−6^
SP_PIR_KEYWORDS	cell adhesion	54	5	2.50 × 10^−8^	1.60 × 10^−6^
SP_PIR_KEYWORDS	postsynaptic cell membrane	24	2.2	3.10 × 10^−8^	1.70 × 10^−6^
SP_PIR_KEYWORDS	membrane	425	39.5	3.30 × 10^−7^	1.70 × 10^−5^
SP_PIR_KEYWORDS	ion transport	62	5.8	1.20 × 10^−6^	5.40 × 10^−5^
SP_PIR_KEYWORDS	voltage-gated channel	25	2.3	2.80 × 10^−6^	1.20 × 10^−4^
SP_PIR_KEYWORDS	transport	137	12.7	3.50 × 10^−6^	1.30 × 10^−4^
SP_PIR_KEYWORDS	Nucleotide-binding	135	12.6	1.40 × 10^−5^	5.00 × 10^−4^
SP_PIR_KEYWORDS	chromosomal rearrangement	35	3.3	1.50 × 10^−5^	5.00 × 10^−4^

**Table 3:** The “functional annotation clustering” tool shows the roles played by the selected genes (enrichment score: 9.19).

Annotation cluster 1	Enrichment score: 9.19	Count	*p*-Value	Benjamini
GOTERM_CC_FAT	cell junction	78	7.2 × 10^−14^	3.7 × 10^−11^
GOTERM_CC_FAT	synapse	59	2.0 × 10^−12^	5.1 × 10^−10^
SP_PIR_KEYWORDS	cell junction	59	1.8 × 10^−11^	2.3 × 10^−9^
SP_PIR_KEYWORDS	synapse	39	1.6 × 10^−10^	1.6 × 10^−8^
GOTERM_CC_FAT	synapse part	41	6.8 × 10^−9^	5.8 × 10^−7^
SP_PIR_KEYWORDS	postsynaptic cell membrane	24	3.1 × 10^−8^	1.7 × 10^−6^
GOTERM_CC_FAT	postsynaptic membrane	27	1.0 × 10^−7^	7.3 × 10^−6^
GOTERM_CC_FAT	postsynaptic density	17	3.4 × 10^−6^	1.8 × 10^−4^

**Table 4:** The “tissue expression” tool shows that genes are preferably expressed in the brain, hippocampus, and amygdala.

Category	Term	Count	%	*p*-Value	Benjamini
UP_TISSUE	Brain	610	56.7	1.4 × 10^−24^	3.9 × 10^−22^
UP_TISSUE	Hippocampus	55	5.1	4.3 × 10^−7^	0.000041
UP_TISSUE	Epithelium	196	18.2	0.000024	0.0017
UP_TISSUE	Amygdala	61	5.7	0.00003	0.0017
UP_TISSUE	Fetal brain	71	6.6	0.00011	0.0053
UP_TISSUE	Retina	36	3.3	0.00072	0.029
UP_TISSUE	Neuron	8	0.7	0.0013	0.041
