# Supplementary material for: A Novel Collection of snRNA-Like Promoters with Tissue-Specific Transcription Properties
Source: Int J Mol Sci. 2012 Sep 11;13(9):11323–32. doi: 10.3390/ijms130911323 (PMC3472747; doi:10.3390/ijms130911323)
Supplement: Supplementary file 1 [file ijms-13-11323-s003.pdf]

Supplementary data S1

Chr	Base number (GENOME ASSEMBLY: genome browser hg17)	Score	Gene	Gene orientation	Position	Dir.	AS	Distance	Distance
								Last nucleotide PSE/First nucleotide TATA	Last nucleotide PSE/First nucleotide Poly(T)
**1**	6355765	1,52	ACOT7	3'-5'	I 1	5'-3'	ok	51	298
	7381569	2,585	CAMTA1	5'-3'	I 5	5'-3'	no	32	381
302 results	7757057	2,135	VAMP3	5'-3'	I 2	5'-3'	no	25	457
	7932262	2,305				5'-3'		34	513
	10592609	2,53	PEX14	5'-3'	I 4	5'-3'	no	37	458
	11790002	2,43	CLCN6	5'-3'	I 1	5'-3'	no	31	458
	21348761	1,485	EIF4G3	3'-5'	I 2	5'-3'	ok	17	507
	23211613	2,24				5'-3'		41	342
	24937294	2,16				5'-3'		42	482
	31401210	2,47				5'-3'		31	368
	32277267	1,705	KHDRBS1	5'-3'	I 7 E 8 I 8	5'-3'	no	49	315
	35785118	2,39	KIAA0319L	3'-5'	I 2	5'-3'	ok	34	322
	38649240	2,69				5'-3'		38	402
	40362445	2,475				5'-3'		40	379
	42726347	2,17				5'-3'		21	408
	46304025	1,82	PIK3R3	3'-5'	I 5 E 5 I 4	5'-3'	ok	16	388
	46346418	1,855	PIK3R3	3'-5'	I 1	5'-3'	ok	19	365
	48958923	1,885	AGBL4	3'-5'	I 2	5'-3'	ok	44	517
	50059740	2,175				5'-3'		49	409
	50919216	2,74	FAF1	3'-5'	I 7	5'-3'	ok	33	402
	50936234	2,53	FAF1	3'-5'	I 7	5'-3'	ok	39	380
	51403114	2,245				5'-3'		42	353
	51641249	2,715	EPS15	3'-5'	I 17 E 18 I 18	5'-3'	ok	32	411
	56265461	2,165				5'-3'		29	491
	66148161	2,43	PDE4B	5'-3'	I 1	5'-3'	no	28	428
	66161132	1,96	PDE4B	5'-3'	I 3	5'-3'	no	51	386
	67072257	2,015	WDR78	3'-5'	E 12 I 11	5'-3'	ok	49	441
	67096433	1,935	WDR78	3'-5'	I 7	5'-3'	ok	21	457

67296911	2,475				5'-3'		31	449
67552721	2,09	IL12RB2	5'-3'	I 1	5'-3'	no	20	402
68979970	2,535				5'-3'		38	447
69293375	1,855				5'-3'		38	583
72494806	1,965	NEGR1	3'-5'	I 1	5'-3'	ok	22	461
73722042	2,66				5'-3'		35	366
73810293	2,215				5'-3'		47	397
75262583	2,755				5'-3'		37	405
75865409	2,335				5'-3'		43	381
76125233	2,76	MSH4	5'-3'	I 15	5'-3'	no	36	422
78276500	1,915				5'-3'		50	367
78362057	1,99	GIPC2	5'-3'	I 4	5'-3'	no	20	436
80004607	2,59				5'-3'		38	436
80268439	2,665				5'-3'		38	397
80798725	2,51				5'-3'		41	396
83371072	2,2				5'-3'		28	474
84912427	2,745	SSX2IP	3'-5'	I 2	5'-3'	ok	37	403
87583455	2,1	LMO4	5'-3'	E 5	5'-3'	no	20	414
88031736	2,355				5'-3'		45	413
89011313	2,425	PKN2	5'-3'	I 6	5'-3'	no	27	419
89385833	1,805	GBP7	3'-5'	E 8 I 7	5'-3'	ok	20	345
89419023	2,785				5'-3'		36	401
89426848	1,885	GBP4	3'-5'	E 8 I 7	5'-3'	ok	20	457
89975820	1,97				5'-3'		47	348
90048898	2,245				5'-3'		45	435
93000533	2,295	EVI5	3'-5'	I 1	5'-3'	ok	24	403
94113366	2,74	DNTTIP2	3'-5'	I 3 E 3 I 2	5'-3'	ok	33	416
94425411	2,52	ARHGAP29	3'-5'	I 15	5'-3'	ok	42	408
95176018	2,375				5'-3'		42	379
96022022	2,24				5'-3'		30	486
98235705	1,47				5'-3'		48	258
98419516	2,605				5'-3'		33	443
98613912	2,16				5'-3'		23	386
101644098	2,24				5'-3'		42	466
102454863	2,26				5'-3'		45	432
102746960	2,2				5'-3'		34	534
103190036	2,09	COL11A1	3'-5'	I 41	5'-3'	ok	23	372
103238865	1,48	COL11A1	3'-5'	I 24	5'-3'	ok	22	558
105732777	2,545				5'-3'		31	435
105994330	2,48				5'-3'		41	428

106142369	2,315				5'-3'		46	411
107411725	2,495				5'-3'		42	403
107988212	2,535	VAV3	3'-5'	I 19	5'-3'	ok	41	417
108227886	2,095	VAV3	3'-5'	I 1	5'-3'	ok	43	333
113636227	1,935				5'-3'		43	517
114374108	2,135				5'-3'		40	311
118178082	2,705				5'-3'		38	405
119656744	1,985				5'-3'		48	457
142339433	2,44				5'-3'		40	372
144518438	1,365	GPR89A	5'-3'	I 5	5'-3'	no	16	521
145614614	2,655				5'-3'		37	385
148347701	2,29	VPS45	5'-3'	I 13	5'-3'	no	24	402
149455583	2,625	PIP5K1A	5'-3'	I 1	5'-3'	no	30	409
157650570	2,1				5'-3'		21	424
158086596	1,76				5'-3'		22	502
161307561	1,935	RGS4	5'-3'	I 1	5'-3'	no	19	381
162891828	2,015	PBX1	5'-3'	I 2	5'-3'	no	18	407
163119348	2,03				5'-3'		46	350
163293570	2,215				5'-3'		30	327
165464688	2,48	POU2F1	5'-3'	I 1	5'-3'	no	28	418
165618445	1,83	POU2F1	5'-3'	I 8	5'-3'	no	19	458
168070952	2,85	C1orf112	5'-3'	I 16	5'-3'	no	35	404
169656768	2,02				5'-3'		18	410
172844824	2,075	RABGAP1L	5'-3'	I 13	5'-3'	no	39	529
174956011	2,195	PAPPA2	5'-3'	I 12	5'-3'	no	34	283
175741104	2,43				5'-3'		37	478
175786228	2,025				5'-3'		33	559
175902859	2,195				5'-3'		23	393
178871895	2,01	XPR1	5'-3'	I 1	5'-3'	no	19	396
180886654	2,35	RGS8	3'-5'	I 4	5'-3'	ok	30	464
181848577	1,24				5'-3'		19	242
185222696	1,505	RGS8	3'-5'	I 17	5'-3'	ok	44	593
185735351	1,615				5'-3'		24	551
186212600	2,01				5'-3'		49	376
187000231	2,565				5'-3'		30	397
189701877	2,1				5'-3'		22	434
192376106	1,345				5'-3'		24	213
194746175	2,26	KCNT2	3'-5'	I 1	5'-3'	ok	26	442
195965174	2,385	DENND1B	3'-5'	I 3	5'-3'	ok	31	351
195978018	1,85	DENND1B	3'-5'	I 2	5'-3'	ok	24	314

197685777	2,375				5'-3'		40	359
198018488	2,78				5'-3'		35	390
198343732	1,9	NR5A2	5'-3'	I 4	5'-3'	no	16	414
200079957	2,35	IPO9	5'-3'	I 1	5'-3'	no	43	434
202427164	2,09	KISS1	3'-5'	I 2	5'-3'	ok	23	446
205693473	2,27				5'-3'		46	398
207350614	2,335				5'-3'		44	427
208242069	2,215	SYT14	5'-3'	I 2	5'-3'	no	23	421
209442714	2,465				5'-3'		42	421
212017509	1,465				5'-3'		22	257
216912955	1,67				5'-3'		23	530
217433524	2,205	LYPLAL1	5'-3'	I 3	5'-3'	no	39	315
220462522	1,905				5'-3'		19	375
222115687	2,755				5'-3'		34	423
223178838	2,82				5'-3'		34	408
223284973	2,39				5'-3'		26	402
224198241	2,06				5'-3'		33	266
229300693	2,11				5'-3'		47	442
229802272	2,03				5'-3'		49	438
236703124	1,935				5'-3'		26	507
236878325	2,325				5'-3'		42	369
239029395	2,06	RGS7	3'-5'	I 17	5'-3'	ok	49	432
239591362	2,35				5'-3'		25	414
239700648	2,355				5'-3'		28	443
241424881	1,81	CEP170	3'-5'	I 6	5'-3'	ok	22	492
241599945	2,24	SDCCAG8	5'-3'	I 12	5'-3'	no	45	382
242396556	2,345				5'-3'		28	373
244374336	2,16	SMYD3	3'-5'	I 5	5'-3'	ok	31	306
246737527	1,915				5'-3'		21	357
2042254	2,265	PRKCZ	5'-3'	I 1	3'-5'	ok	24	397
5279019	2,735				3'-5'		36	427
12538241	2,54				3'-5'		30	392
13382877	2,745				3'-5'		36	393
13603707	2,745				3'-5'		36	393
20636128	2,74				3'-5'		37	402
25040563	2,41	CLIC4	5'-3'	E 6	3'-5'	ok	33	482
28752839	2,2	TRSPAP1	5'-3'	I 2	3'-5'	ok	31	504
31188917	2,135	PUM1	3'-5'	I 18	3'-5'	no	24	447
31766276	1,765				3'-5'		54	441
32376879	1,245	KPNA6	5'-3'	I 1	3'-5'	ok	51	575

33262324	1,9	AK2	3'-5'	I 2 E 2 I 1	3'-5'	no	28	534
33555612	2,44	A3GALT2	3'-5'	I 1	3'-5'	no	29	382
33780070	2,435	CSMD2	3'-5'	I 57	3'-5'	no	40	447
42595578	2,62				3'-5'		32	388
43295325	2,445				3'-5'		32	465
43295404	1,35				3'-5'		52	544
45704795	2,26	TESK2	3'-5'	I 1	3'-5'	no	43	452
47403525	2,3				3'-5'		45	394
49507401	2,25				3'-5'		23	404
50968928	2,28	FAF1	3'-5'	I 6	3'-5'	no	31	330
52797787	2,405				3'-5'		26	405
52894173	2,715	MGC52498	3'-5'	I 2	3'-5'	no	32	407
58502081	2,245				3'-5'		27	363
58798279	2,6				3'-5'		31	424
60016844	2,39				3'-5'		40	362
60758655	2,155				3'-5'		45	365
62155335	1,565	INADL	5'-3'	I 26	3'-5'	ok	20	521
62721888	1,98	DOCK7	3'-5'	I 42	3'-5'	no	27	310
63536191	2,435				3'-5'		27	417
65069974	2,395	RAVER2	5'-3'	E 12	3'-5'	ok	40	363
66443088	1,915	PDE4B	5'-3'	I 1	3'-5'	ok	46	327
68647775	2,155				3'-5'		25	453
69666539	2,185				3'-5'		27	467
70269113	1,705	LRRC7	5'-3'	I 16	3'-5'	ok	25	543
71290443	2,195				3'-5'		47	425
71964919	2,27	NEGR1	3'-5'	I 3	3'-5'	no	25	388
73430290	2,875				3'-5'		35	409
74039954	2,785				3'-5'		34	401
74151612	2,25				3'-5'		33	514
74836339	2,48	C1orf173	3'-5'	I 11	3'-5'	no	42	400
75264764	2,665				3'-5'		39	411
76842780	1,775	ST6GALNAC3	5'-3'	I 3	3'-5'	ok	17	369
78558951	1,76				3'-5'		20	336
79070812	1,46				3'-5'		44	216
79562332	2,43				3'-5'		29	438
79704627	1,84				3'-5'		53	436
80053760	1,845				3'-5'		19	455
81940473	1,965				3'-5'		19	431
82852681	2,515				3'-5'		41	421
84840786	1,985				3'-5'		30	537

84849373	2,05				3'-5'		30	524
86083921	2,295	COL24A1	3'-5'	I 39	3'-5'	no	46	403
87146119	2,06	SEP15	3'-5'	I 1	3'-5'	no	23	452
88207236	2,25				3'-5'		32	504
88592591	2,635				3'-5'		39	401
90045617	2,13				3'-5'		44	468
90287995	1,93				3'-5'		29	538
91223833	1,71	ZNF644	3'-5'	I 1	3'-5'	no	23	296
92177508	1,85				3'-5'		46	504
93433752	1,38	CCDC18	5'-3'	I 6	3'-5'	ok	51	270
**94341472**	**1,49**	**ABCA4**	**3'-5'**	**I 4**	**3'-5'**	no	**24**	**576**
94423584	1,875	ARHGAP29	3'-5'	E 15 I 16	3'-5'	no	17	389
95093651	1,74	SLC44A3	5'-3'	I 8	3'-5'	ok	22	506
95199584	2,01				3'-5'		23	462
97607436	1,72	DPYD	3'-5'	I 16	3'-5'	no	20	328
98442090	2,445				3'-5'		42	425
99271749	2,625				3'-5'		33	379
105023346	2,255				3'-5'		27	453
105094502	2,5				3'-5'		28	404
105480660	1,95				3'-5'		27	304
105704127	2,275				3'-5'		43	369
106248303	2,265				3'-5'		47	407
106802057	1,605				3'-5'		49	295
107811591	2,195	NTNG1	5'-3'	I 5	3'-5'	ok	41	333
108093823	2,09	VAV3	3'-5'	I 13	3'-5'	no	23	446
111549811	2,48				3'-5'		38	360
112154844	2,035	KCND3	3'-5'	I 2	3'-5'	no	19	401
112378587	2,565				3'-5'		33	451
113165755	2,13				3'-5'		40	310
117381555	1,92				3'-5'		22	348
**117733853**	**1,785**	**MAN1A2**	**5'-3'**	**I 1**	**3'-5'**	ok	**28**	**261**
119219722	2,265				3'-5'		47	411
143646917	2,225	PDE4DIP	3'-5'	I 5	3'-5'	no	43	359
145209646	2,14	CHD1L	5'-3'	I 11	3'-5'	ok	22	392
145886471	1,365	GPR89B	5'-3'	I 5	3'-5'	ok	16	521
148560687	2,33	PRPF3	5'-3'	I 1	3'-5'	ok	41	360
152130794	2,005	GATAD2B	3'-5'	I 1	3'-5'	no	21	443
159417984	1,945				3'-5'		25	495
160999923	2,28	DDR2	5'-3'	I 10	3'-5'	ok	26	438
161397038	2,005	RGS5	3'-5'	I 3	3'-5'	no	43	315

161851650	1,555				3'-5'		52	503
162290057	1,725				3'-5'		53	459
162638556	1,92				3'-5'		54	408
166576146	2,175				3'-5'		39	509
167620866	2,35	BLZF1	5'-3'	I 6	3'-5'	ok	41	364
168270960	2,42	KIFAP3	3'-5'	I 6 E 6 I 5	3'-5'	no	40	450
168814428	1,495				3'-5'		48	555
170611250	2,11	DNM3	5'-3'	I 17	3'-5'	ok	46	366
171757558	2,345	SLC9A11	3'-5'	I 21	3'-5'	no	32	333
172230159	2,64				3'-5'		34	372
172870770	2,22	RABGAP1L	5'-3'	I 13	3'-5'	ok	26	368
174122195	2,325				3'-5'		28	369
174534115	2,315				3'-5'		40	471
175375890	1,91	ASTN1	3'-5'	I 1	3'-5'	no	24	492
175837578	1,22				3'-5'		20	228
176192020	2,58	SEC16B	3'-5'	I 10	3'-5'	no	30	400
179821608	2,725	CACNA1E	5'-3'	I 6	3'-5'	ok	38	409
180103955	2,12				3'-5'		24	450
180431963	2,16				3'-5'		21	406
182701815	1,385	C1orf21	5'-3'	I 1	3'-5'	ok	49	567
183540280	2,21	IVNS1ABP	3'-5'	I 8	3'-5'	no	32	512
184952249	2,11				3'-5'		50	406
185516856	2,3				3'-5'		37	314
186265520	2,22				3'-5'		26	450
187332624	2,645				3'-5'		39	415
187352423	2,04				3'-5'		27	496
187463551	2,435				3'-5'		36	331
187903818	2,08				3'-5'		47	448
189077045	1,88				3'-5'		53	428
190070812	2,8				3'-5'		36	414
193263754	2,08				3'-5'		44	478
194293424	2,34				3'-5'		32	486
195194521	2,065	CFHR2	5'-3'	I 4 E 5	3'-5'	ok	49	431
195202911	2,235				3'-5'		33	301
195797632	2,075	DENND1B	3'-5'	I 15	3'-5'	no	41	309
196730827	2,315				3'-5'		46	407
197609930	2,41				3'-5'		26	406
197805183	1,84				3'-5'		20	352
200390282	2,15	PTPN7	3'-5'	I 5	3'-5'	no	21	414
200616043	2,035	PPP1R12B	5'-3'	I 1	3'-5'	ok	48	447

	201571853	2,05				3'-5'		47	454
	202073481	2,185	ZC3H11A	5'-3'	I 9 E 10 I 10	3'-5'	ok	37	291
	202247422	2,23				3'-5'		42	350
	207075408	2,805				3'-5'		34	405
	**209221620**	**1,965**	**KCNH1**	**3'-5'**	**I 6**	**3'-5'**	no	**18**	**421**
	209384137	1,95				3'-5'		51	384
	211223367	2,48	VASH2	5'-3'	I 5	3'-5'	ok	30	380
	211337157	1,89	RPS6KC1	5'-3'	I 3	3'-5'	ok	53	392
	214167236	2,31	USH2A	3'-5'	I 38	3'-5'	no	41	462
	214518150	2,06	USH2A	3'-5'	I 11	3'-5'	no	34	562
	216699503	1,9				3'-5'		52	434
	217258004	2,09				3'-5'		46	362
	219940735	2,425				3'-5'		37	479
	220401972	1,925				3'-5'		54	409
	220898720	2,565	MIA3	5'-3'	E 19 I 19	3'-5'	ok	30	421
	221629614	2,115				3'-5'		44	471
	222055076	1,94	TP53BP2	3'-5'	E 10 I 9	3'-5'	no	53	416
	223210880	1,695	C1orf67	5'-3'	I 3	3'-5'	ok	47	293
	223770053	1,845	ENAH	3'-5'	I 6	3'-5'	no	51	363
	225852133	2,18	ZNF678	5'-3'	I 1	3'-5'	ok	23	428
	227179568	1,5				3'-5'		23	564
	228737718	2,105				3'-5'		22	385
	230685247	2,275	SIPA1L2	3'-5'	I 4	3'-5'	no	25	429
	232459387	2,79	SLC35F3	5'-3'	I 3	3'-5'	ok	36	416
	235555353	2,19	RYR2	5'-3'	I 2	3'-5'	ok	27	352
	236037071	1,76	RYR2	5'-3'	I 99	3'-5'	ok	19	472
	237588888	1,945				3'-5'		19	435
	238198567	2,105				3'-5'		23	443
	240600567	2,135	PLD5	3'-5'	I 2	3'-5'	no	24	447
	241457515	2,785	CEP170	3'-5'	I 1	3'-5'	no	36	417
	246106980	1,805	TRIM58	5'-3'	E 6	3'-5'	ok	28	553
	246193181	1,945	OR2L13	5'-3'	I 1	3'-5'	ok	45	323
	246769758	1,915				3'-5'		21	357
	246856699	2,345	OR2T11	3'-5'	E 1	3'-5'	no	30	465
**Ø**				**63**			**131**	**33,92**	**414,65**
				**68**			**65**	**31,65**	**414,37**
				**131**			**66**	**35,65**	**414,85**

**2**	3427633	2,24	TTC15	5'-3'	I 6	5'-3'	no	37	302
	5157819	2,525				5'-3'		39	439
350 results	9596480	2,25	ADAM17	3'-5'	I 16	5'-3'	ok	26	374
	9974260	1,745	TAF1B	5'-3'	I 12	5'-3'	no	21	323
	11292458	2,19	ROCK2	3'-5'	I 5	5'-3'	ok	48	416
	13261328	2,205				5'-3'		47	395
	13311634	2,42				5'-3'		41	440
	14121185	2,555				5'-3'		38	443
	16622167	2,16	FAM49A	3'-5'	I 3	5'-3'	ok	45	366
	17064812	2,445				5'-3'		42	425
	17526114	2,6				5'-3'		34	364
	17802144	2,625	FLJ40869	5'-3'	I 1	5'-3'	no	34	449
	18392885	2,035				5'-3'		19	401
	18417512	1,65				5'-3'		16	354
	18663927	2,765				5'-3'		35	387
	19834422	2,06				5'-3'		46	462
	20829325	1,79	C2orf43	3'-5'	I 4	5'-3'	ok	25	292
	21307706	2,705				5'-3'		33	395
	21888717	2,06				5'-3'		42	502
	27734402	2,265	SUBT7L	5'-3'	I 3	5'-3'	no	24	397
	33258720	2,275	LTBP1	5'-3'	I 4/1	5'-3'	no	28	459
	33687382	1,83				5'-3'		16	390
	34271952	1,795				5'-3'		29	253
	35043797	1,565				5'-3'		17	327
	35362932	1,895				5'-3'		26	515
	35864030	2,53				5'-3'		40	390
	37597747	2,055				5'-3'		48	443
	38650789	2,68	HNRPLL	3'-5'	I 7	5'-3'	ok	34	438
	38665918	2,605	HNRPLL	3'-5'	I 3 E 3 I 2	5'-3'	ok	34	453
	38826574	2,56	SFRS7	3'-5'	I 7 E 7 I 6	5'-3'	ok	29	412
	41600006	2,37				5'-3'		40	460
	43806235	2,38	PLEKHH2	5'-3'	I 13	5'-3'		30	360
	45462730	2,035				5'-3'		22	447
	45714208	2,465				5'-3'		27	411
	48398689	2,47	FOXN2	5'-3'	I 1	5'-3'	no	38	358
	50949499	2,185	NRXN1	3'-5'	I 3	5'-3'	ok	38	517
	51002985	2,425	NRXN1	3'-5'	I 4 E 3 I 3/E 3 I 3	5'-3'	ok	28	429

52132423	1,605				5'-3'		50	305
52462288	1,675				5'-3'		23	289
53180511	2,31				5'-3'		27	376
53820203	2,115	ASB3	3'-5'	I 3	5'-3'	ok	20	411
53964347	1,8	PSME4	3'-5'	I 29	5'-3'	ok	22	494
55346816	2,355	MTIF2	3'-5'	I 4 E 4 I 4/I	5'-3'	ok	25	405
56641812	2,725				5'-3'		38	409
56825729	2,41				5'-3'		34	326
57062876	2,13				5'-3'		32	528
57423121	2,015				5'-3'		44	327
57457934	1,71				5'-3'		25	542
58077650	2,145				5'-3'		23	435
60311315	2,215				5'-3'		48	407
61149154	2,225	KIAA1841	5'-3'	I 1 E 2 I 2	5'-3'	no	30	489
61426047	2,605	USP34	3'-5'	I 9	5'-3'	ok	39	395
63576363	2,245	LOC51057	3'-5'	I 1	5'-3'	ok	28	465
64282238	2,03				5'-3'		49	380
66136076	1,505				5'-3'		47	255
66681979	2,69				5'-3'		35	372
66997294	2,25				5'-3'		24	394
67169288	2,82				5'-3'		35	420
67785248	2,475				5'-3'		42	399
68061413	2,68				5'-3'		38	400
68777904	1,655				5'-3'		47	533
69069188	2,19				5'-3'		44	362
72873453	2,32	EXOC6B	3'-5'	I 1	5'-3'	ok	30	348
74669401	1,885	LOC130951	3'-5'	I 4	5'-3'	ok	21	467
79319026	2,34				5'-3'		29	456
81017603	2,03				5'-3'		51	418
83724978	2,725				5'-3'		33	399
83737809	2,7				5'-3'		37	394
86812670	2,645	RMND5A	5'-3'	I 1	5'-3'	no	33	435
87046351	2,7	RANBP2	5'-3'	I 7	5'-3'	no	36	384
88123921	2,37	KRCC1	3'-5'	I 1	5'-3'	ok	44	398
91095692	2,48				5'-3'		32	360
91555828	1,875				5'-3'		16	419
91570597	2,55				5'-3'		41	404
99654780	1,775	AFF3	3'-5'	I 7	5'-3'	ok	20	479
100723312	2,215				5'-3'		48	407
102476740	2,05	SLC9A4	5'-3'	I 2	5'-3'	no	50	394

103437796	2,1				5'-3'		38	534
104971744	2,39				5'-3'		32	342
107094824	2,015				5'-3'		31	541
107230464	1,735				5'-3'		26	271
107307596	2,07				5'-3'		35	570
107833929	2,63				5'-3'		36	370
**109931042**	**2,7**	**RANBP2**	**5'-3'**	**I 7**	**5'-3'**	**no**	**36**	**384**
110668361	2,01				5'-3'		29	522
113579030	1,86				5'-3'		18	442
115255681	2,2	DPP10	5'-3'	I 1	5'-3'	**no**	44	454
116665800	2,7				5'-3'		38	404
117815318	2,385				5'-3'		29	371
119051707	2,685				5'-3'		32	417
123846373	2,6				5'-3'		38	384
127068068	2,305				5'-3'		34	305
132384025	1,765				5'-3'		48	317
132801750	1,855				5'-3'		18	443
133374214	2,6	NAP5	3'-5'	I 8	5'-3'	ok	40	404
133704331	2,105	NAP5	3'-5'	I 4	5'-3'	ok	21	423
134146479	1,17				5'-3'		19	590
134637786	2,625				5'-3'		31	419
136229156	2,42	UBXD2	5'-3'	I 4 E 4 I 5	5'-3'	no	41	378
137180845	2,27				5'-3'		46	398
139660996	2,135				5'-3'		21	401
139985876	2,535				5'-3'		41	417
140235483	2,36				5'-3'		45	412
147452261	2,145				5'-3'		21	403
148029380	2,255				5'-3'		38	315
151187791	2,025				5'-3'		38	269
154611845	2,04	GALNT13	5'-3'	I 3	5'-3'	no	21	436
154985465	2	GALNT13	5'-3'	I 10	5'-3'	no	26	494
155284239	2,155	KCNJ3	5'-3'	I 2	5'-3'	no	48	395
158891707	2,05	LOC130940	3'-5'	I 7	5'-3'	ok	27	324
163442616	2,555				5'-3'		38	443
163912360	1,895				5'-3'		24	323
164125563	2,095				5'-3'		29	505
164785730	2,595				5'-3'		32	383
164799663	1,84				5'-3'		16	392
165752289	2,585	SCN3A	3'-5'	I 1	5'-3'	ok	31	427
165946061	2,315	SCN2A	3'-5'	I 25	5'-3'	ok	44	431

166013817	1,82				5'-3'		18	450
166545502	2,07				5'-3'		20	398
166598363	2,585	SCN1A	3'-5'	I 16	5'-3'	ok	33	371
168260130	1,415				5'-3'		21	561
169205000	2,23	LASS6	5'-3'	I 4	5'-3'	no	23	418
169616326	1,69				5'-3'		17	466
171530877	2,3	GORASP2	5'-3'	E 10	5'-3'	no	28	454
173570343	2,365	RAPGEF4	5'-3'	I 16	5'-3'	no	38	481
176230415	2,12				5'-3'		21	398
176760251	1,975				5'-3'		39	269
180234592	2,065	ZNF533	3'-5'	I 3	5'-3'	ok	24	357
181010885	1,89				5'-3'		41	546
181040990	2,11				5'-3'		30	306
182493107	2,7	SSFA2	5'-3'	I 14 E 15 I 15	5'-3'	no	35	444
182877786	1,945	PDE1A	3'-5'	I 2	5'-3'	ok	17	403
184899918	2,535				5'-3'		40	391
185031528	2,55				5'-3'		38	444
187187842	2,65	ITGAV	5'-3'	I 2	5'-3'	no	39	414
188387377	2,37				5'-3'		44	420
188463100	2,13				5'-3'		25	360
188547788	2,245				5'-3'		27	455
190447142	2,59	PMS1	5'-3'	I 12	5'-3'	no	31	426
191508785	2,39	GLS	5'-3'	I 14	5'-3'	no	38	342
**191722160**	**2,22**	**STAT4**	**3'-5'**	**I 1**	**5'-3'**	ok	**42**	**470**
193136628	2,8				5'-3'		34	414
196110956	2,08				5'-3'		32	280
196227699	1,925				5'-3'		43	299
196402644	2,705	DNAH7	3'-5'	I 48	5'-3'	ok	35	375
197749304	2,015	ANKRD44	3'-5'	I 2	5'-3'	ok	38	551
197749829	2,15	ANKRD44	3'-5'	I 2	5'-3'	ok	49	414
199811251	2,29				5'-3'		25	426
200174161	2,66				5'-3'		32	396
203566417	2,21				5'-3'		28	472
204437987	1,36				5'-3'		24	602
204929158	2,67				5'-3'		37	388
**207260487**	**2,415**	**DYTN**	**3'-5'**	**I 9**	**5'-3'**	ok	**28**	**431**
208243187	1,86				5'-3'		50	462
208333967	2,08				5'-3'		46	458
208688195	1,97				5'-3'		45	328
209483474	2,22				5'-3'		45	440

210025776	1,635	MAP2	5'-3'	I 1	5'-3'	no	16	467
210647222	2,27	FLJ23861	3'-5'	I 6	5'-3'	ok	38	500
211681490	2,44				5'-3'		27	416
213412402	2,105				5'-3'		48	433
214064425	2,735	SPAG16	5'-3'	I 10	5'-3'	no	35	381
214943371	2,71	SPAG16	5'-3'	I 15	5'-3'	no	33	396
216634487	2,365	PECR	3'-5'	I 3	5'-3'	ok	35	307
221245654	2,145				5'-3'		41	495
221516127	1,955				5'-3'		41	533
222752038	1,975				5'-3'		48	359
225958930	2,165				5'-3'		49	411
227024309	2,115				5'-3'		31	297
227609447	2,77	COL4A4	3'-5'	I 37	5'-3'	ok	33	410
228829328	1,79				5'-3'		47	506
231624181	2,655				5'-3'		37	433
234740901	2,545				5'-3'		39	383
238614951	2,35	UBE2F	5'-3'	E 10	5'-3'	no	26	394
1058861	2,16	SNTG2	5'-3'	I 1	3'-5'	ok	22	396
2307288	2,15	MYT1L	3'-5'	I 1	3'-5'	no	27	474
2408404	1,53				3'-5'		20	290
6950181	2,355	RSAD2	5'-3'	I 4	3'-5'	ok	33	325
7175488	2,03				3'-5'		51	418
7513232	2,61				3'-5'		30	406
8111116	2,66				3'-5'		34	442
8257724	2,385				3'-5'		44	401
14127693	1,545				3'-5'		19	303
14234565	2,65				3'-5'		35	454
18892610	2,015				3'-5'		45	481
22276745	2,13				3'-5'		21	400
23169270	2,495				3'-5'		38	455
26205952	2,135	RAB10	5'-3'	I 5	3'-5'	ok	47	381
28091503	2,155	BRE	5'-3'	I 4	3'-5'	ok	23	433
30706649	1,92	LYCAT	5'-3'	I 5	3'-5'	ok	23	480
32528039	2,12	BIRC6	5'-3'	I 22	3'-5'	ok	21	398
32555020	2,425	BIRC6	5'-3'	I 34	3'-5'	ok	44	409
34934670	2,395				3'-5'		43	393
36516264	2,335	CRIM1	5'-3'	I 2	3'-5'	ok	42	447
36540819	2,46	CRIM1	5'-3'	I 4	3'-5'	ok	33	346
38103908	2,55	FAM82A	5'-3'	I 10	3'-5'	ok	36	464
38936304	2,465	DHX57	3'-5'	I 7	3'-5'	no	32	461

39318666	2,52				3'-5'		42	410
41000999	2,485				3'-5'		38	361
43837288	1,975	PLEKHH2	5'-3'	I 25 E 26	3'-5'	ok	22	459
44485990	2,14	C2orf34	5'-3'	I 3	3'-5'	ok	48	392
**46190211**	**2,195**	**PRKCE**	**5'-3'**	**I 11**	**3'-5'**	ok	**31**	**313**
51194572	2,58				3'-5'		34	360
52394341	2,08				3'-5'		23	448
52453194	2,18				3'-5'		39	310
52486718	2,14				3'-5'		48	392
52532268	2,06				3'-5'		19	406
53675323	1,905				3'-5'		23	335
54447017	2,705				3'-5'		36	433
56395437	1,83	CCDC85A	5'-3'	I 2	3'-5'	ok	48	330
57841128	2,145				3'-5'		41	323
58004469	2,605				3'-5'		31	423
59120420	1,585				3'-5'		17	331
66616541	1,915	MEIS1	5'-3'	I 8	3'-5'	ok	16	407
66632783	2,37	MEIS1	5'-3'	I 9	3'-5'	ok	40	358
66680586	2,715				3'-5'		33	397
67631312	1,79				3'-5'		17	446
67706239	1,79				3'-5'		50	476
67742895	2,21				3'-5'		48	406
68209857	2,48				3'-5'		38	360
68686325	2,225				3'-5'		24	429
76715992	1,495				3'-5'		45	585
76933754	2,16				3'-5'		27	472
**76984839**	**2,04**				**3'-5'**		**26**	**332**
78168606	2,07				3'-5'		44	480
78714908	2,82				3'-5'		35	398
79596949	2,245	CTNNA2	5'-3'	I 1	3'-5'	ok	44	445
79922460	2,56	CTNNA2	5'-3'	I 3	3'-5'	ok	37	366
80302494	1,64	CTNNA2	5'-3'	I 7	3'-5'	ok	51	322
80865500	2,005				3'-5'		19	423
82141079	1,635				3'-5'		51	321
82970266	2,15				3'-5'		46	444
84726172	1,87				3'-5'		54	398
86675325	1,52				3'-5'		27	600
87144709	2,635				3'-5'		33	437
87451721	2,48				3'-5'		32	360
87884213	2,7	RANBP2	5'-3'	I 7	3'-5'	ok	36	384

88787522	1,705	RPIA	5'-3'	I 3	3'-5'	ok	54	453
89939284	2,48				3'-5'		32	360
91055159	2,04				3'-5'		49	382
91385049	2,55				3'-5'		41	404
91547457	2,48				3'-5'		32	360
98335572	1,74	CNGA3	5'-3'	I 1	3'-5'	ok	50	332
98709096	2,475	MGAT4A	3'-5'	I 2 E 2 I 1	3'-5'	no	39	369
98749121	1,635				3'-5'		30	607
**99704768**	**2,04**	**AFF3**	**3'-5'**	**I 10**	**3'-5'**	no	**38**	**272**
101789763	2	MAP4K4	5'-3'	I 3	3'-5'	ok	34	574
106427176	2,625				3'-5'		36	369
108633863	1,99	LIMS1	5'-3'	I 1	3'-5'	ok	29	526
110009502	2,01				3'-5'		29	522
**110746876**	**2,7**	**RANBP2**	**5'-3'**	**I 7**	**3'-5'**	ok	**36**	**384**
**111028347**	**2,7**	**RANBP2**	**5'-3'**	**I 7**	**3'-5'**	ok	**36**	**384**
111385607	2,71	ACOXL	5'-3'	I 5	3'-5'	ok	36	432
**112883327**	**2,71**				**3'-5'**		**36**	**384**
113334940	2,39				3'-5'		44	416
113372209	2,045				3'-5'		24	353
114676184	2,42				3'-5'		37	480
115243229	2,16	DPP10	5'-3'	I 1	3'-5'	ok	47	386
117160563	1,755				3'-5'		16	443
117334572	2,32				3'-5'		43	440
118627376	1,65				3'-5'		49	514
120510204	2,16	EPB41L5	5'-3'	I 2	3'-5'	ok	38	522
122032234	1,7	CLASP1	3'-5'	I 2	3'-5'	no	53	464
125624528	2,785				3'-5'		34	401
126281248	1,49				3'-5'		48	556
129504686	2,21				3'-5'		24	386
129795956	2,405				3'-5'		42	385
131661195	2,185				3'-5'		41	487
135745422	2,4	ZRANB3	3'-5'	I 10 E 9 I 9	3'-5'	no	43	424
135761852	1,61	ZRANB3	3'-5'	I 8	3'-5'	no	18	326
138672050	2,745				3'-5'		36	425
140587680	2,445				3'-5'		43	403
140754342	2,535	LRP1B	3'-5'	I 84	3'-5'	no	29	417
141565710	2,33	LRP1B	3'-5'	I 7	3'-5'	no	39	478
142797961	2,24				3'-5'		24	392
144222102	2,74	ARHGAP15	5'-3'	I 13	3'-5'	ok	37	416
**144761816**	**0,99**	**GTDC1**	**3'-5'**	**I 1**	**3'-5'**	no	**16**	**596**

145557193	1,635				3'-5'		54	351
145984636	2,37				3'-5'		45	408
146510044	1,88				3'-5'		53	390
147952660	2,54				3'-5'		30	392
148714301	2,21				3'-5'		45	442
149391013	1,6	KIF5C	5'-3'	I 1	3'-5'	ok	44	574
151588496	2,38				3'-5'		29	370
155213219	2,33				3'-5'		28	370
156104525	2,17				3'-5'		25	368
156217188	1,735				3'-5'		16	447
156551961	2,585				3'-5'		39	427
157600891	1,775				3'-5'		17	449
157943862	2,155				3'-5'		23	433
160520116	2,17	PLA2R1	3'-5'	I 23 E 22 I 22	3'-5'	no	21	408
160685618	2,65	ITGB6	3'-5'	I 12	3'-5'	no	31	404
165061865	2,375	GRB14	3'-5'	2 E 12 I 11 E 11 I	3'-5'	no	44	419
165614199	2,685				3'-5'		32	401
166036507	2,37				3'-5'		43	430
167797571	1,9	XIRP2	3'-5'	I 3	3'-5'	no	17	394
170729769	2,455				3'-5'		35	493
172291315	2,41	DYNC1I2	5'-3'	I 10 E 10 I 11	3'-5'	ok	42	432
172703662	2,185				3'-5'		46	437
173341513	2,335	RAPGEF4	5'-3'	I 1	3'-5'	ok	25	417
176876808	2,715	MTX2	5'-3'	I 4	3'-5'	ok	38	407
177186397	2,19				3'-5'		42	476
177577453	2,815				3'-5'		36	411
180286915	2,23	ZNF533	3'-5'	I 3	3'-5'	no	45	380
181723081	2,03				3'-5'		20	390
181869168	2,67				3'-5'		32	420
182652500	2,665	PPP1R1C	5'-3'	I 4	3'-5'	ok	38	421
184385795	1,65				3'-5'		17	344
184699353	2,135				3'-5'		24	447
184735510	2,015				3'-5'		51	397
185100004	2,465				3'-5'		42	421
185752201	1,92				3'-5'		19	440
187072461	1,64	ZC3H15	5'-3'	I 2	3'-5'	ok	44	252
187356648	1,62				3'-5'		54	348
188969302	2,655	GULP1	5'-3'	I 2	3'-5'	ok	34	375
189987527	2,625				3'-5'		30	409
190770152	1,16	MGC13057	5'-3'	I 1	3'-5'	ok	54	562

	191040348	2,1	FLJ20160	5'-3'	I 1	3'-5'	ok	23	374
	191152758	1,52				3'-5'		54	328
	192778120	2,09				3'-5'		24	456
	193311549	2,315				3'-5'		43	377
	193936987	1,99				3'-5'		18	416
	194527119	1,94				3'-5'		17	416
	195022625	1,79				3'-5'		41	252
	195881946	2,16				3'-5'		21	406
	196252606	1,555	SLC39A10	5'-3'	I 1 E 2	3'-5'	ok	18	315
	196618088	2,195	DNAH7	3'-5'	I 5	3'-5'	no	22	403
	200999468	1,895	LOC26010	5'-3'	I 6	3'-5'	ok	18	383
	204758479	2,365				3'-5'		32	481
	205900683	1,95	PARD3B	5'-3'	I 18	3'-5'	ok	51	384
	209586550	2,04				3'-5'		50	392
	210946812	2,07				3'-5'		43	328
	**214673622**	**2,6**	**SPAG16**	**5'-3'**	**I 13**	**3'-5'**	ok	**37**	**374**
	215533510	2,055	ABCA12	3'-5'	I 31	3'-5'	no	24	355
	220585235	2,305				3'-5'		39	335
	223135274	1,765				3'-5'		51	347
	223165925	2,495	FARSB	3'-5'	I 16	3'-5'	no	40	383
	224061178	1,195				3'-5'		51	585
	**224595517**	**2,335**	**SERPINE2**	**3'-5'**	**I 1**	**3'-5'**	no	**26**	**391**
	224626969	1,96				3'-5'		53	412
	226588104	2,715				3'-5'		37	421
	227060218	2,02				3'-5'		44	328
	229900722	2,715				3'-5'		32	411
	230826346	2,235	SP140	5'-3'	E 11 I 11	3'-5'	ok	33	517
	231328189	1,14	CAB39	5'-3'	I 1	3'-5'	ok	50	212
	231531046	1,925				3'-5'		20	449
	232600964	2,065	DIS3L2	5'-3'	I 4	3'-5'	ok	41	307
	233257532	2,71				3'-5'		35	442
	238629207	2,565				3'-5'		41	407
	239355143	2,4		74		3'-5'	ok	42	434
**Ø**				53			**127**	**34,47**	**410,72**
				**127**			**46**	**33,84**	**409,87**
							**81**	**34,82**	**411,21**

**3**	424939	2,355	CLH1	5'-3'	E 28 I	5'-3'	no	43	385
	1480916	1,95				5'-3'		21	454
319 results	2572960	2,675	CNTN4	5'-3'	I 2	5'-3'	no	37	389
	3785297	1,365				5'-3'		17	287
	5933560	1,835				5'-3'		16	391
	6123765	2,345				5'-3'		43	383
	7377138	2,725	GRM7	5'-3'	I 4	5'-3'	no	32	409
	9540437	2,695	LHFPL4	3'-5'	I 2	5'-3'	ok	32	403
	11030346	2,025	SLC6A1	5'-3'	I 1	5'-3'	no	32	549
	11753633	1,865				5'-3'		20	461
	14126115	1,79				5'-3'		21	332
	15071259	2,405	MRPS25	3'-5'	I 2	5'-3'	ok	31	355
	15627004	2,28	BTD	5'-3'	I 1	5'-3'	no	27	370
	16935153	2,67	PLCL2	5'-3'	I 1	5'-3'	no	31	410
	20691173	1,405				5'-3'		46	225
	21256635	2,28				5'-3'		25	390
	21982928	2,125				5'-3'		43	479
	24576149	2,265				5'-3'		25	387
	25039688	2,545				5'-3'		40	393
	26309729	2,615				5'-3'		40	411
	26996978	1,98				5'-3'		23	350
	28437213	1,96	ZCWPW2	5'-3'	I 2	5'-3'	no	46	336
	29602004	1,78	RBMS3	5'-3'	I 3	5'-3'	no	17	370
	31538471	2,665				5'-3'		39	411
	34035619	2,085				5'-3'		50	401
	34532173	2,585				5'-3'		35	467
	37039677	1,94	MLH1	5'-3'	I 11	5'-3'	no	22	466
	37110514	2,21	LRRFIP2	3'-5'	I 16	5'-3'	ok	25	442
	41138641	1,94				5'-3'		20	372
	42912518	1,515				5'-3'		43	217
	44212160	2,065				5'-3'		51	411
	45039427	2,14				5'-3'		46	372
	46086079	2,725				5'-3'		32	409
	**46256742**	**2,38**				**5'-3'**		**43**	**428**
	47312834	1,935	KLHL18	5'-3'	I 1	5'-3'	no	44	507
	47667898	2,705	SMARCC1	3'-5'	I 21	5'-3'	ok	38	405
	49192086	1,925	LOC646498	3'-5'	I 2	5'-3'	ok	26	309
	53026806	1,765	SFMBT1	3'-5'	I 2	5'-3'	ok	30	581

53474919	1,955				5'-3'		23	345
54670550	1,965	CACNA2D3	5'-3'	I 11	5'-3'	no	49	451
58824161	2,145	FLJ42117	3'-5'	I 12 E 12 I 11	5'-3'	ok	44	465
58944404	1,98	FLJ42117	3'-5'	I 4	5'-3'	ok	41	528
59838737	2,385	FHIT	3'-5'	I 8	5'-3'	ok	34	497
61321510	2,525				5'-3'		36	349
64432029	2,2				5'-3'		23	424
**64569513**	**2,245**	**ADAMTS9**		**I 22**	**5'-3'**		**45**	**383**
**64569513**	**2,245**	**ADAMTS9**	**3'-5'**	**I 22**	**5'-3'**	ok	**45**	**383**
64580430	2,41	ADAMTS9	**3'-5'**	I 19	5'-3'	ok	36	326
64581158	2,18	ADAMTS9	**3'-5'**	I 19	5'-3'	ok	22	418
67327514	2,455				5'-3'		29	385
67348460	2,19				5'-3'		45	372
68254599	2,295	FAM19A1	5'-3'	I 2	5'-3'	no	35	293
68938327	1,705	FAM19A4	3'-5'	I 3	5'-3'	ok	30	593
69520609	2,21				5'-3'		47	422
71267878	2,085	FOXP1	3'-5'	I 6	5'-3'	ok	34	557
73457664	2,485				5'-3'		38	457
75783139	2,22				5'-3'		47	420
76390341	2,85				5'-3'		35	414
76556019	2,13				5'-3'		26	468
78565964	2,325				5'-3'		28	369
78679833	2,18				5'-3'		28	478
79146673	1,99	ROBO1	3'-5'	I 2	5'-3'	ok	44	322
81170864	2,7				5'-3'		32	404
81445109	2,01				5'-3'		50	432
82160393	1,93				5'-3'		18	390
83075569	2,155				5'-3'		23	433
83288932	2,55				5'-3'		31	434
83848433	1,685				5'-3'		49	311
84120383	2,565				5'-3'		40	421
85446772	1,775				5'-3'		18	459
85969602	2,245	CADM2	5'-3'	I 2	5'-3'	no	40	333
86696934	2,71				5'-3'		34	386
87636925	2,65				5'-3'		36	374
89548773	2,25	EPHA3	5'-3'	I 10	5'-3'	no	32	504
89654638	2,39				5'-3'		31	352
89856094	1,965				5'-3'		21	367
95500863	2,08				5'-3'		46	458
96183481	1,99				5'-3'		18	402

99015790	2,6				5'-3'		31	424
99816263	2,255				5'-3'		24	395
100009507	2,795	DCBLD2	3'-5'	I 13 E 13 I 12	5'-3'	ok	34	415
100377917	2,835				5'-3'		35	417
100619757	2,325				5'-3'		41	459
101478759	2,725	TBC1D23	5'-3'	I 1	5'-3'	no	35	379
102108155	2,45	ABI3BP	3'-5'	I 2	5'-3'	ok	27	414
103144757	2,145				5'-3'		49	415
103664780	2,225	ZPLD1	5'-3'	I 6	5'-3'	no	46	429
105079471	1,95				5'-3'		18	394
106213661	1,34				5'-3'		16	292
110944082	1,81				5'-3'		21	482
112618651	2,77				5'-3'		34	420
116285676	2,19	ZBTB20	3'-5'	I 1	5'-3'	ok	48	416
117891163	1,84				5'-3'		24	506
119694812	1,985				5'-3'		27	311
121329662	1,935				5'-3'		18	427
122217486	1,04	STXBP5L	5'-3'	I 4	5'-3'	no	17	222
133161846	1,84	CPNE4	3'-5'	I 1	5'-3'	ok	21	476
133697897	2,6	DNAJC13	5'-3'	I 35 E 36 I 36	5'-3'	no	39	424
133733894	2,195	DNAJC13	5'-3'	I 55	5'-3'	no	46	383
134011580	2,245				5'-3'		23	403
134606184	2,36	BFSP2	5'-3'	I 1	5'-3'	no	34	316
138570516	2,605				5'-3'		39	423
139997227	1,835				5'-3'		36	211
141796401	2,49				5'-3'		39	372
142757264	1,735	RASA2	5'-3'	I 6 E 7 I 7	5'-3'	no	43	261
143875607	2,34	PLS1	5'-3'	I 4	5'-3'	no	42	446
143938000	2,11	TRPC1	5'-3'	E 2 I 2	5'-3'	no	21	422
144724254	1,775	SLC9A9	3'-5'	I 9	5'-3'	ok	19	469
146762367	2,1				5'-3'		21	394
147864895	1,615				5'-3'		51	501
148368104	2,145				5'-3'		23	383
154067497	2,21				5'-3'		46	432
154879909	2,31				5'-3'		24	412
156294028	2,405	MME	5'-3'	I 3	5'-3'	no	43	423
158210135	1,92	FLJ16641	5'-3'	I 10	5'-3'	no	19	440
159321508	2,345	RSRC1	5'-3'	I 1	5'-3'	no	30	353
159380010	2,49	RSRC1	5'-3'	I 3	5'-3'	no	28	416
159870080	2,59	GFM1/LXN	5'-3'	I 13/E 3 I 2	5'-3'	no	31	392

160948034	2,505	SCHIP1	5'-3'	I 1	5'-3'	no	28	413
**161194922**	**2,495**	**IL12A**	**5'-3'**	**I 6**	**5'-3'**	no	**28**	**403**
161306812	2,655				5'-3'		31	405
163475914	2,065				5'-3'		25	347
163730349	2,36				5'-3'		26	422
163827517	1,775				5'-3'		25	529
164366329	2,065				5'-3'		48	377
165873261	2,61				5'-3'		33	376
165901425	2,785				5'-3'		36	401
166039444	2,055				5'-3'		42	315
166372048	2,14				5'-3'		48	426
168224439	2,015				5'-3'		19	397
168566363	2,425	FLJ23049	3'-5'	E 6 I 5	5'-3'	ok	26	409
170441912	2,445	MDS1	3'-5'	I 2	5'-3'	ok	28	393
170848764	2,305	MDS1	3'-5'	I 1	5'-3'	ok	24	405
172299199	2,295	TNIK	3'-5'	I 22	5'-3'	ok	25	425
172994973	2,13				5'-3'		49	418
173705836	2,625	TNFSF10	3'-5'	E 5	5'-3'	ok	37	379
173764463	2,77				5'-3'		33	408
**175253010**	**2,84**	**NLGN1**	**5'-3'**	**I 4**	**5'-3'**	no	**35**	**416**
175859121	2,145				5'-3'		44	353
176364094	2,385	NAALADL2	5'-3'	I 2	5'-3'	no	40	457
178288638	1,84	TBL1XR1	3'-5'	I 2	5'-3'	ok	18	446
178340408	2,17	TBL1XR1	3'-5'	I 1	5'-3'	ok	40	500
178373878	2,48	TBL1XR1	3'-5'	I 1	5'-3'	ok	33	468
181230895	2,365	PEX5L	3'-5'	I 1	5'-3'	ok	28	377
181922897	1,9				5'-3'		22	474
182169362	2,715	FXR1	5'-3'	I 14	5'-3'	no	38	411
182326264	2,645				5'-3'		34	373
188008146	1,96				5'-3'		26	502
189194399	2,135				5'-3'		46	447
189242421	1,9				5'-3'		41	274
189341250	2,525				5'-3'		40	429
189975067	2,08	LPP	5'-3'	I 8	5'-3'	no	46	360
190049709	2,04	LPP	5'-3'	I 8	5'-3'	no	47	456
190679331	2,325				5'-3'		45	399
190725723	1,845				5'-3'		45	303
191255906	2,055	LEPREL1	3'-5'	I 1	5'-3'	ok	19	413
191291841	2,065	LEPREL1	3'-5'	I 1	5'-3'	ok	19	411
192319110	1,705				5'-3'		47	523

193182275	1,985				5'-3'		20	381
193522731	2,44	FGF12	3'-5'	I 4	5'-3'	ok	31	456
198480012	2,205	DLG1	3'-5'	I 4	5'-3'	ok	26	453
368837	1,935	CHL1	5'-3'	I 10	3'-5'	ok	20	447
1570990	1,7				3'-5'		16	454
1579665	2,545				3'-5'		36	353
1975605	2,22				3'-5'		42	348
2357330	2,805	CNTN4	5'-3'	I 2	3'-5'	ok	36	413
3197013	2,125				3'-5'		20	409
3229711	2,49				3'-5'		34	342
3605441	1,96				3'-5'		19	432
5307564	2,59				3'-5'		32	382
5647334	2,47				3'-5'		33	470
6124421	2,375				3'-5'		25	409
**8396155**	**1,96**				**3'-5'**		**17**	**412**
9410728	2,095				3'-5'		49	393
12299422	2,215				3'-5'		26	451
12617977	2,52	RAF1	3'-5'	I 7	3'-5'	no	29	398
13096864	2,49				3'-5'		33	352
13758693	1,93				3'-5'		46	488
15436175	2,03	METTL6	3'-5'	I 3	3'-5'	no	23	458
18554774	2,42				3'-5'		26	408
19886868	2,27				3'-5'		46	420
20130679	2,28	PCAF	5'-3'	I 6	3'-5'	ok	28	360
20432740	1,635				3'-5'		42	587
20683884	2,255				3'-5'		31	493
20897922	2,005				3'-5'		20	433
22191771	2,665				3'-5'		32	421
23451074	2,77	UBE2E2	5'-3'	I 3	3'-5'	ok	34	420
24688765	1,865				3'-5'		20	461
26051724	2,35				3'-5'		36	504
27299391	2,665	NEK10	3'-5'	I 5	3'-5'	no	38	397
27706570	2,61				3'-5'		39	396
27800098	2,11				3'-5'		20	406
28732349	2,575				3'-5'		29	409
29603369	1,17	RBMS3	3'-5'	I 3 E 4 I 4	3'-5'	no	18	580
29746391	2,695	RBMS3	3'-5'	I 4	3'-5'	no	33	425
33128205	1,145				3'-5'		52	585
33811721	1,875				3'-5'		22	339
34248960	1,83				3'-5'		51	458

34873335	1,55				3'-5'		45	244
35351487	2,545				3'-5'		35	343
39993842	1,8	MYRIP	5'-3'	I 2	3'-5'	ok	16	384
41333926	2,135	ULK4	3'-5'	I 35	3'-5'	no	21	417
42290912	2,505				3'-5'		29	395
50072906	2,43	RBM6	5'-3'	I 11 E 12 I 12	3'-5'	ok	33	478
51114453	2,39	DOCK3	5'-3'	I 9	3'-5'	ok	33	332
51289237	1,65	DOCK3	5'-3'	I 25	3'-5'	ok	48	524
53484037	2,25				3'-5'		47	404
56438769	2,51				3'-5'		39	376
56586266	2,515	CCDC66	5'-3'	I 7	3'-5'	ok	33	461
58653083	2,28				3'-5'		24	400
62864322	2,695				3'-5'		34	435
65590400	1,95	MAGI1	3'-5'	I 1	3'-5'	no	50	374
66774083	2,785				3'-5'		35	427
70867741	2,115				3'-5'		27	481
71164732	1,715	FOXP1	3'-5'	I 11	3'-5'	no	17	461
72116060	1,415				3'-5'		54	307
72473246	2,46				3'-5'		29	432
74830953	2,07				3'-5'		20	398
75831774	2,02				3'-5'		49	440
76255638	2,69				3'-5'		38	416
77631778	2,7	ROBO2	5'-3'	I 5	3'-5'	ok	38	404
78183511	2,38				3'-5'		28	380
79006724	2,795	ROBO1	3'-5'	I 3	3'-5'	no	36	415
79254106	2,695	ROBO1	3'-5'	I 2	3'-5'	no	32	403
79985737	2,565				3'-5'		40	397
82067030	1,515				3'-5'		18	307
84805750	2,39				3'-5'		29	446
86122776	2,325	CADM2	5'-3'	I 8	3'-5'	ok	45	399
87041721	2,47				3'-5'		43	408
87372620	1,57	CHMP2B	5'-3'	I 1	3'-5'	ok	22	278
87626271	1,85				3'-5'		20	354
88201008	2,56				3'-5'		34	356
90196407	2,5				3'-5'		30	434
95604766	2,52				3'-5'		32	450
99716446	1,64	CLDND1	3'-5'	E 6	3'-5'	no	51	322
100232595	2,365				3'-5'		25	411
102780998	1,55	PCNP	5'-3'	I 1 E 2 I 2	3'-5'	ok	53	324
103473027	2,465				3'-5'		42	397

104458711	2,37				3'-5'		44	398
107238627	2,41				3'-5'		35	316
107291333	1,92				3'-5'		16	408
107599424	1,915				3'-5'		48	471
108735000	2,63	BBX	5'-3'	I 2	3'-5'	ok	38	390
108809412	1,41	BBX	5'-3'	I 2	3'-5'	ok	24	592
109877172	1,615	DZIP3	5'-3'	I 24 E 25 I 25	3'-5'	ok	54	347
111563461	2,84				3'-5'		35	402
111766790	2,27				3'-5'		47	408
111907731	1,915				3'-5'		46	327
112087658	1,97				3'-5'		24	480
113895932	2,13				3'-5'		39	300
114197070	1,945	GTPBP8	5'-3'	I 2	3'-5'	ok	28	293
115858434	2,49	ZBTB20	3'-5'	I 5	3'-5'	no	38	456
116624076	2,385				3'-5'		42	381
117381370	1,54	LSAMP	3'-5'	I 1	3'-5'	no	26	586
117938429	2,365				3'-5'		45	407
118159189	2,3				3'-5'		43	374
118460969	2,12				3'-5'		44	348
118934755	2,135				3'-5'		48	427
119337721	2,335				3'-5'		26	391
123291650	2,045	CD86	5'-3'	I 1	3'-5'	ok	27	495
124656801	2,065				3'-5'		38	541
125727992	1,95	KALRN	5'-3'	I 33	3'-5'	ok	23	344
129971824	2,19	RAB7A	5'-3'	I 1	3'-5'	ok	38	302
131998690	1,095				3'-5'		18	223
132122876	2,02	ATP2C1	5'-3'	I 1	3'-5'	ok	18	408
133147804	1,975	CPNE4	3'-5'	I 1	3'-5'	no	46	479
136478702	2,595				3'-5'		36	363
137923277	2,42	STAG1	3'-5'	I 1	3'-5'	no	42	430
139728799	1,81	CEP70	3'-5'	I 10	3'-5'	no	50	346
140367105	2,46				3'-5'		27	412
140589826	2,54	COPB2	3'-5'	I 1	3'-5'	no	37	362
143667026	2,6	ATR	3'-5'	I 40	3'-5'	no	39	394
144347769	2,185				3'-5'		42	477
144588094	1,82	SLC9A9	3'-5'	I 12	3'-5'	no	16	388
145438150	2,165				3'-5'		49	407
145445135	2,52				3'-5'		42	410
149143550	2,465				3'-5'		41	431
149420613	2,105				3'-5'		21	423

151776812	1,95	EIF2A	5'-3'	I 11	3'-5'	ok	50	374
152858371	2,5				3'-5'		40	384
153979140	2,275				3'-5'		26	379
153982135	2,075				3'-5'		22	379
154642902	2,225				3'-5'		45	439
158227275	2,19	FlJ16641	5'-3'	I 12	3'-5'	ok	45	372
161930008	2,605				3'-5'		38	433
161959548	1,865	PPM1L	5'-3'	I 1	3'-5'	ok	19	367
162959919	2,12				3'-5'		32	288
163156616	2,555				3'-5'		30	395
164114625	2,54				3'-5'		36	352
165171757	2,22				3'-5'		48	408
165902065	2,05				3'-5'		49	384
168827870	2,685	WDR49	3'-5'	I 1	3'-5'	no	35	371
169418533	1,475				3'-5'		44	219
169805069	2,42				3'-5'		42	430
173334959	2,035	FNDC3B	5'-3'	I 2	3'-5'	ok	50	427
176608530	1,955	NAALADL2	5'-3'	I 5	3'-5'	ok	53	405
176906214	2,03	NAALADL2	5'-3'	I 11	3'-5'	ok	22	448
177064534	1,63				3'-5'		16	350
177183978	1,795				3'-5'		32	595
178207903	2,17				3'-5'		49	410
179035733	2,465				3'-5'		42	397
179574953	2,36				3'-5'		25	406
180843534	2,245				3'-5'		47	415
181419688	2,585				3'-5'		30	417
182090805	2,475				3'-5'		43	409
182162953	1,92	FXR1	5'-3'	11 E 12 I 12 E 1[illegible]	3'-5'	ok	42	530
182732589	2,255				3'-5'		23	405
183585446	2,12				3'-5'		49	420
183692432	2,68				3'-5'		38	418
183897016	2,31				3'-5'		26	432
184011020	1	ATP11B	5'-3'	I 1	3'-5'	ok	16	224
185347034	1,885				3'-5'		53	427
189221302	2,03				3'-5'		25	478
190824017	2,345				3'-5'		36	505
192557778	2,035	CCDC50	5'-3'	I 2	3'-5'	ok	44	331
192753029	2,555				3'-5'		30	395
196545375	1,84	CENTB2	3'-5'	I 5	3'-5'	no	41	262
196885928	1,655	SDHA	5'-3'	E 10 I 10	3'-5'	ok	47	285

	198633974	1,485				3'-5'		45	587
	198959323	2,295				3'-5'		27	373
**Ø**				**66**			**120**	**33,9**	**406,27**
				**54**			**56**	**33,6**	**409,88**
				**120**			**64**	**34,1**	**404,1**
**4**	655452	1,97			**I**	5'-3'		43	308
	4641068	2,37				5'-3'		45	410
295 results	6887166	2,3	KIAA0232	3'-5'	**I** 3	5'-3'	ok	30	344
	10884323	1,9				5'-3'		21	354
	11164606	1,76				5'-3'		16	442
	12618092	2,24				5'-3'		45	382
	17505160	2,655	LCORL	3'-5'	**I** 5	5'-3'	ok	31	405
	18541852	2,19				5'-3'		23	426
	19744867	1,7				5'-3'		51	484
	20691190	1,755	KCNIP4	3'-5'	**I** 1	5'-3'	ok	50	335
	22474522	2,21				5'-3'		47	422
	22621487	2,745				5'-3'		34	425
	23989253	2,55				5'-3'		30	394
	29421278	2,515				5'-3'		33	461
	29650237	1,865				5'-3'		50	357
	29898854	2,755				5'-3'		34	423
	31599793	1,465				5'-3'		17	307
	33006953	2,6				5'-3'		34	364
	34012078	2,055				5'-3'		46	355
	34042682	1,535				5'-3'		17	321
	34601788	2,435				5'-3'		42	391
	36369653	1,525				5'-3'		44	229
	36382550	2,115				5'-3'		48	431
	**40670981**	**1,905**	**APBB2**	**3'-5'**	**I 6**	**5'-3'**	ok	**44**	**305**
	41507389	1,94				5'-3'		20	446
	42265800	2,805	ATP8A1	3'-5'	**I** 15 E 15 **I** 14	5'-3'	ok	35	423
	42774618	2,77				5'-3'		33	408
	44388884	1,555	GUF1	5'-3'	**I** 13	5'-3'	no	21	285
	45770156	2,13	GABRB1	5'-3'	**I** 3	5'-3'	no	49	418

46857429	2,745	GABRB1	5'-3'	I 3 E 4	5'-3'	no	35	435
52789551	1,87				5'-3'		34	218
53471315	2,695	SCFD2	3'-5'	I 6	5'-3'	ok	37	425
55466254	1,77				5'-3'		32	218
55896597	2,115				5'-3'		48	431
58517131	1,945				5'-3'		21	455
59641317	2,505				5'-3'		39	375
60711891	2,63				5'-3'		34	370
61165423	2,225				5'-3'		44	369
61507140	2,15				5'-3'		35	264
61742799	2,025				5'-3'		19	419
62235562	2,65	LPHN3	5'-3'	I 3	5'-3'	no	31	404
62540581	2,375	LPHN3	5'-3'	I 16	5'-3'	no	44	419
63133906	2,585				5'-3'		30	401
64573101	2,215				5'-3'		30	491
65044736	2,305				5'-3'		44	385
65589827	2,52				5'-3'		35	480
66672734	2,31				5'-3'		46	406
69831278	2,41	UGT2A3	3'-5'	I 4 E 4 I 3	5'-3'	ok	43	422
70464924	1,935				5'-3'		17	417
70766388	2,23				5'-3'		26	448
72643136	2,265	SLC4A4	5'-3'	I 19	5'-3'	no	25	387
74207632	2,26	ANKRD17	3'-5'	I 18	5'-3'	ok	31	326
74894915	2,415				5'-3'		44	411
80013449	1,52	BMP2K	5'-3'	I 13	5'-3'	no	51	520
82518331	2,705				5'-3'		32	405
83243263	1,89				5'-3'		49	352
84394129	2,4				5'-3'		40	454
84615537	2,575	CCDC98	3'-5'	I 3	5'-3'	ok	41	409
85965237	1,9	WDFY3	3'-5'	I 10	5'-3'	ok	19	444
87237265	1,93	MAPK10	3'-5'	I 9	5'-3'	ok	25	320
89283230	2,245	ABCG2	3'-5'	I 1	5'-3'	ok	47	403
91772575	2,645	MGC48628	5'-3'	I 6	5'-3'	no	37	435
91855192	1,33	MGC48628	5'-3'	I 6	5'-3'	no	47	220
93657852	2,255	GRID2	5'-3'	I 1	5'-3'	no	44	443
94422125	2,06	GRID2	5'-3'	I 8	5'-3'	no	45	346
96503288	2,215	UNC5C	3'-5'	I 1	5'-3'	ok	47	421
97364710	2,51				5'-3'		30	432
97524758	1,97				5'-3'		17	408
98660695	2,145				5'-3'		47	435

100019264	2,14				5'-3'		48	426
103040195	2,565	BANK1	5'-3'	I 6	5'-3'	no	35	347
104466852	1,865				5'-3'		50	357
104577484	2,055				5'-3'		25	345
105891272	2,435				5'-3'		41	381
106079702	2,435				5'-3'		38	351
109345602	2,61				5'-3'		30	412
112174435	2,425				5'-3'		29	379
112856637	2,245				5'-3'		23	415
114581884	2,755				5'-3'		37	405
116151874	2,74	NDST4	3'-5'	I 2	5'-3'	ok	37	416
117433621	2,16				5'-3'		49	406
117631048	2,315				5'-3'		29	357
117789315	2,32				5'-3'		45	420
118075000	2,8				5'-3'		34	404
120047020	2,715	SYNPO2	5'-3'	I 1	5'-3'	no	38	407
120111147	2,575	SYNPO2	5'-3'	I 1	5'-3'	no	30	399
120727143	2,715	PDE5A	3'-5'	I 3	5'-3'	ok	34	387
121955200	1,735	PRDM5	3'-5'	I 7	5'-3'	ok	51	477
122052841	2,63	PRDM5	3'-5'	I 1	5'-3'	ok	39	418
123414826	2,195	KIAA1109	5'-3'	I 46 E 47 I 47	5'-3'	no	25	373
124723482	1,855				5'-3'		45	513
124924251	2,525				5'-3'		36	349
128274756	2				5'-3'		31	274
131207488	2,02				5'-3'		44	490
133322957	2,14				5'-3'		21	416
134411802	2,73				5'-3'		33	400
134616900	1,615				5'-3'		45	561
135504691	2,265				5'-3'		30	481
138961111	2,315				5'-3'		24	407
139778937	2,595				5'-3'		31	393
141193605	1,845	MAML3	3'-5'	I 1	5'-3'	ok	19	455
143409639	1,86	INPP4B	3'-5'	I 7	5'-3'	ok	17	432
145010187	2,325				5'-3'		39	479
145793723	2,25	HHIP	5'-3'	I 2	5'-3'	no	47	404
146944510	2,72	LOC152485	3'-5'	I 8	5'-3'	ok	33	420
151447039	2,47	LRBA	3'-5'	I 53	5'-3'	ok	36	480
**151558698**	**2,065**	**LRBA**	3'-5'	**I 47**	**5'-3'**	ok	**49**	**431**
152080516	1,985	LRBA	3'-5'	I 2	5'-3'	ok	28	517
152546545	2,22				5'-3'		28	470

152546647	2,37				5'-3'		28	378
152662008	2,295	LOC729830	5'-3'	I 3	5'-3'	no	30	475
155490864	2,765	DCHS2	3'-5'	I 6	5'-3'	ok	33	411
155918700	2,56				5'-3'		37	452
156090938	2,605				5'-3'		33	375
156246634	1,855				5'-3'		16	423
161336453	2,71				5'-3'		32	412
162172746	2,495				5'-3'		29	393
162854071	2,34	FSTL5	3'-5'	I 6	5'-3'	ok	27	382
163402671	2,625				5'-3'		31	419
163971833	2,835				5'-3'		35	401
164560583	2,355				5'-3'		28	375
165357573	1,89				5'-3'		18	382
167689773	2,35				5'-3'		32	484
167727541	2,21				5'-3'		43	356
168116312	2,66	SPOCK3	3'-5'	I 4	5'-3'	ok	32	396
170015570	2,155	PALLD	5'-3'	I 10	5'-3'	no	21	413
170384127	2,295	SH3RF1	3'-5'	I 2	5'-3'	ok	42	455
171408987	2,065				5'-3'		25	471
172983746	2,395	GALNT17	5'-3'	I 2	5'-3'	no	26	403
174738083	1,98				5'-3'		18	400
177475032	1,765				5'-3'		15	387
180028259	2,28				5'-3'		46	400
181194494	1,91				5'-3'		44	306
181345624	1,89				5'-3'		45	312
182652039	2,62				5'-3'		40	408
183281811	2,735				5'-3'		34	391
184229479	2,7				5'-3'		32	414
188150887	2,625				5'-3'		31	419
189901877	1,98				5'-3'		48	458
190238994	2,235				5'-3'		23	417
5693684	2,775	EVC2	3'-5'	I 9	3'-5'	no	37	409
8501896	2,56				3'-5'		41	406
10132874	2,18	MIST	3'-5'	I 14	3'-5'	no	43	350
11960517	1,965				3'-5'		53	411
11964941	2,405				3'-5'		43	423
13022952	2,81	RAB28	3'-5'	I 4	3'-5'	no	34	406
13859771	2,555				3'-5'		29	413
14383072	2,25				3'-5'		35	534
16046334	1,815				3'-5'		19	357

16120853	1,405	LDB2	3'-5'	I 6	3'-5'	no	24	593
16849254	2,645				3'-5'		37	435
18390963	2,105				3'-5'		50	413
19637038	2,7				3'-5'		35	444
20311986	2,52	C4orf28	5'-3'	I 1	3'-5'	ok	42	410
20832592	2,01	KCNIP4	3'-5'	I 1	3'-5'	no	23	462
20961744	2,055	KCNIP4	3'-5'	I 1	3'-5'	no	27	493
21750274	2,675				3'-5'		33	389
23268745	2,175				3'-5'		25	449
24759689	1,635	SEPSECS	3'-5'	I 5	3'-5'	no	40	607
25300417	1,985				3'-5'		32	261
25386608	1,695	KIAA0746	3'-5'	I 17 E 17 I 16	3'-5'	no	22	515
28481393	1,9				3'-5'		43	524
29742377	2,595				3'-5'		33	445
30146315	2,565				3'-5'		41	407
33015721	2,43				3'-5'		41	438
34748645	2,825				3'-5'		34	409
37142224	2,085	C4orf19	5'-3'	I 1	3'-5'	ok	21	391
38175809	2,465				3'-5'		29	387
41427336	2,52				3'-5'		36	348
41471799	2,345				3'-5'		25	403
45192157	2,82				3'-5'		36	410
45747115	1,885	GABRG1	3'-5'	I 8	3'-5'	no	49	351
47742217	1,675				3'-5'		29	229
48273535	2,06	FRYL	3'-5'	I 21	3'-5'	no	31	532
52468893	1,76	DCUN1D4	5'-3'	I 8	3'-5'	ok	49	326
58699469	1,58				3'-5'		51	508
59350207	2,13				3'-5'		22	390
59351292	1,81				3'-5'		49	482
59804312	2,295				3'-5'		31	485
59993811	2,44				3'-5'		41	436
61189947	2,7				3'-5'		35	444
**62329201**	**2,34**	**LPHN3**	**5'-3'**	**I 5**	**3'-5'**	ok	**30**	**466**
63967242	1,805				3'-5'		50	473
64781785	2,07				3'-5'		48	378
64793494	2,385				3'-5'		27	427
65329530	2,37				3'-5'		42	378
66680582	1,895				3'-5'		51	373
67037600	1,985				3'-5'		47	351
67189210	2,415				3'-5'		44	407

69251675	2,185				3'-5'		25	447
69603675	2,185				3'-5'		25	447
70149347	2,41				3'-5'		43	422
70256715	2,34				3'-5'		43	436
70436047	2,355				3'-5'		45	405
70965890	2,42				3'-5'		39	358
73087917	2,225				3'-5'		42	469
73200256	2,715	NPFFR2	5'-3'	I 1	3'-5'	ok	36	431
73912602	2,26				3'-5'		47	412
76233047	2,585				3'-5'		40	417
78521864	2,1				3'-5'		44	344
78547450	2,385				3'-5'		42	437
79542071	2,56	FRAS1	5'-3'	I 30	3'-5'	ok	29	412
83981944	2,455	SEC31A	3'-5'	I 22 E 22	3'-5'	no	27	405
**87040883**	**2,14**	**ARHGAP24**	**5'-3'**	**I 3**	**3'-5'**	ok	**22**	**426**
87663820	2,185				3'-5'		23	427
91578035	2,485	MGC48628	5'-3'	I 4	3'-5'	ok	30	381
91764513	2,03	MGC48628	5'-3'	I 5	3'-5'	ok	47	360
91919728	2,44	MGC48628	5'-3'	I 7	3'-5'	ok	42	392
95100071	2,675				3'-5'		31	409
95207506	2,285				3'-5'		29	351
96403394	2,235	UNC5C	5'-3'	I 4	3'-5'	ok	43	457
96454777	2,165	UNC5C	5'-3'	I 2	3'-5'	ok	21	407
96972505	2,22				3'-5'		48	410
97355725	2,43				3'-5'		40	448
98321491	1,795				3'-5'		54	435
100173631	2,525	METAP1	5'-3'	I 2	3'-5'	ok	29	399
**100273562**	**2,11**	**ADH4**	**3'-5'**	**I 5**	**3'-5'**	no	**43**	**336**
100899557	2,46				3'-5'		36	336
105823918	2,12				3'-5'		21	420
106128642	1,755				3'-5'		53	365
106431095	1,94				3'-5'		18	426
107927818	2,4				3'-5'		40	364
109381194	1,79				3'-5'		24	302
109463953	1,79				3'-5'		38	596
110957823	2,135	NOLA1	5'-3'	I 2	3'-5'	ok	49	417
115045241	2,67	ARSJ	3'-5'	I 2	3'-5'	no	33	388
116205315	2,22	NDST4	3'-5'	I 2	3'-5'	no	24	430
117544141	2,65				3'-5'		33	434
118118125	1,945				3'-5'		43	303

118365061	2,425				3'-5'		40	449
119624523	1,81				3'-5'		22	492
120062656	2,13	SYNPO2	5'-3'	I 1	3'-5'	ok	41	320
121494191	1,535				3'-5'		19	301
121556234	2,515				3'-5'		37	461
**124251630**	**2,425**	**SPATA5**	**5'-3'**	**I 14**	**3'-5'**	ok	**26**	**409**
124362520	2,765	SPATA5	**5'-3'**	I 14	3'-5'	ok	35	387
124740772	2,61				3'-5'		39	422
124834162	2,125				3'-5'		24	449
125403195	2,615				3'-5'		34	367
125471766	2,455				3'-5'		27	413
126130514	2,135				3'-5'		23	381
126595716	2,02	FAT4	5'-3'	I 9	3'-5'	ok	43	318
127142234	2,11				3'-5'		21	422
128546768	2,61				3'-5'		30	412
129162665	2,57	C4orf29	5'-3'	I 9	3'-5'	ok	41	408
131167128	1,7				3'-5'		50	494
132014640	1,79				3'-5'		23	506
133458763	2,76				3'-5'		35	386
133643413	2,395				3'-5'		26	403
133813535	2,72				3'-5'		37	398
133998636	2,22				3'-5'		25	440
134997722	2,555				3'-5'		39	433
136270298	1,82				3'-5'		22	490
138352509	2,685				3'-5'		32	417
139084911	2,855				3'-5'		35	405
139540119	2,09				3'-5'		50	402
143500717	2,22	INPP4B	3'-5'	I 4	3'-5'	no	47	420
143635435	2,075	INPP4B	3'-5'	I 2	3'-5'	no	33	549
144423774	2,465				3'-5'		41	431
145429050	2,17				3'-5'		37	530
147729456	1,62				3'-5'		41	600
152582837	1,9	LOC729830	5'-3'	I 1	3'-5'	ok	22	474
153123229	2,2				3'-5'		41	484
153846228	2,225				3'-5'		46	429
155293993	1,37				3'-5'		48	580
155442104	2,135	DCHS2	3'-5'	I 17	3'-5'	no	38	291
156241679	2,445				3'-5'		38	465
156586443	2,185				3'-5'		45	371
156978274	1,505	ACCN5	3'-5'	I 7	3'-5'	no	24	245

	157209133	2,235				3'-5'		47	401
	159053292	1,895				3'-5'		52	435
	**162537727**	**2,005**	**FSTL5**	**5'-3'**	**I 15**	**3'-5'**	ok	**51**	**395**
	162861432	1,7	FSTL5	**5'-3'**	I 6	3'-5'	ok	54	454
	164311594	2,375				3'-5'		26	399
	164760716	2,035				3'-5'		47	361
	164848965	2,555				3'-5'		39	385
	164880755	1,005				3'-5'		17	603
	165636226	1,68				3'-5'		54	458
	166941791	2,125				3'-5'		27	479
	167790721	2,42				3'-5'		32	348
	168187709	2,13	SPOCK3	3'-5'	I 3	3'-5'	no	21	400
	**173133742**	**1,97**	**GALNT17**	**5'-3'**	**I 2**	**3'-5'**	ok	**22**	**358**
	173192103	1,695	GALNT17	**5'-3'**	I 2	3'-5'	ok	53	353
	175096427	1,965				3'-5'		21	367
	175385713	2,465				3'-5'		43	411
	178168703	1,7				3'-5'		49	314
	178909073	1,475	LOC285501	5'-3'	I 2	3'-5'	ok	18	299
	179158911	2,505				3'-5'		37	355
	180234033	1,645				3'-5'		43	575
	181701669	2,18				3'-5'		26	360
	181744517	2,425				3'-5'		29	439
	182672158	2,395				3'-5'		43	393
	184478228	2,225	CLDN22	3'-5'	E 1	3'-5'	no	23	419
	187211277	2,38				3'-5'		42	380
	187460843	1,785				3'-5'		49	331
**Ø**				**44**			**91**	**35,34**	**409,03**
				**47**			**39**	**34,91**	**407,07**
				**91**			**52**	**35,52**	**409,91**
**5**	1645715	1,625			**I**	5'-3'		47	279
	4101847	2,045				5'-3'		19	415
242 results	10315899	1,535	CCT5	5'-3'	I 9 E 10 I 10	5'-3'	no	50	291
	12844111	2,03				5'-3'		40	528
	15777853	2,535	FBXL7	5'-3'	I 2	5'-3'	no	41	401
	18759882	2,75				5'-3'		36	394
	**22420974**	**2,525**	**CDH12**	**3'-5'**	**I 3**	**5'-3'**	ok	**29**	**419**
	25779127	2,26				5'-3'		40	482

26358242	2,645				5'-3'		33	383
28043454	2,445				5'-3'		32	353
28830695	2,025				5'-3'		30	529
29599262	1,905				5'-3'		47	335
34541321	1,68				5'-3'		20	320
36376341	1,31				5'-3'		16	286
37617612	1,89	WDR70	5'-3'	I 9	5'-3'	no	16	402
38123185	2,03				5'-3'		24	468
39725895	2,375				5'-3'		27	429
41513066	2,1	PLCXD3	3'-5'	I 1	5'-3'	ok	42	324
41887281	2,44	OXCT1	3'-5'	I 4	5'-3'	ok	30	372
42915869	2,15				5'-3'		36	544
43128658	1,43				5'-3'		19	538
44059993	2,715				5'-3'		36	387
44955862	1,795				5'-3'		17	373
45362186	1,96	HCN1	3'-5'	I 5	5'-3'	ok	18	396
49673498	2,12				5'-3'		20	408
51049413	2,485				5'-3'		38	361
51346918	2,205				5'-3'		26	365
53475039	2,755	ARL15	3'-5'	I 3	5'-3'	ok	34	423
54277189	2,29				5'-3'		45	426
57171257	2,71				5'-3'		37	422
59048382	2,565	PDE4D	3'-5'	I 1	5'-3'	ok	32	377
59895892	2,17				5'-3'		25	450
60707430	2,155				5'-3'		23	433
61310319	1,845				5'-3'		26	525
64275513	2,285	SDCCAG10	5'-3'	I 11	5'-3'	no	43	447
64529514	1,98	ADAMTS6	3'-5'	I 20	5'-3'	ok	19	390
71621888	2,485	MRPS27	3'-5'	I 4	5'-3'	ok	40	437
72337296	2,435	FCHO2	5'-3'	I 5	5'-3'	no	39	361
72743806	2,67				5'-3'		39	410
74578470	1,825				5'-3'		19	359
75086217	2,82				5'-3'		36	408
75666379	2,37				5'-3'		33	490
**76210266**	**2,01**	**S100Z**	**5'-3'**	**I 4**	**5'-3'**	no	**19**	**422**
76795204	2,695	WDR41	3'-5'	I 3	5'-3'	ok	33	425
79806054	2,175	ZFYVE16	5'-3'	I 16 E 17 I 17	5'-3'	no	21	409
81516197	2,535	ATG10	5'-3'	I 5	5'-3'	no	32	447
82077124	2,195				5'-3'		25	373
82527623	2,865	XRCC4	5'-3'	I 4	5'-3'	no	35	407

85198999	2,37				5'-3'		41	450
87847657	2,385				5'-3'		43	427
91347413	2,7				5'-3'		33	394
91511314	1,855				5'-3'		21	345
92005978	2,065				5'-3'		21	387
92349356	1,715				5'-3'		47	521
93372133	2,155	C5orf21	3'-5'	I 3	5'-3'	ok	40	315
93577607	1,64				5'-3'		19	496
94238906	2,745	MCTP1	3'-5'	I 12	5'-3'	ok	34	425
95480377	2,64				5'-3'		35	362
96956236	2,47				5'-3'		27	408
98810007	2,27				5'-3'		46	398
99171219	2,12				5'-3'		49	398
100152507	2,11				5'-3'		47	442
100741107	1,92				5'-3'		20	368
101569365	2,685				5'-3'		33	427
105031941	2,67				5'-3'		38	398
105059712	2,71				5'-3'		37	422
105579844	2,11				5'-3'		30	512
109170035	2,53	MAN2A1	5'-3'	I 12	5'-3'	no	40	390
112035420	2,685				5'-3'		38	401
112590506	2,18	MCC	3'-5'	I 1	5'-3'	ok	41	488
113306812	1,505				5'-3'		18	305
117140540	2,055				5'-3'		27	325
117243150	1,895				5'-3'		50	455
118209928	1,875	DTWD2	3'-5'	I 5	5'-3'	ok	25	509
**118865620**	**2,81**	**HSD17B4**	**5'-3'**	**I 13 E 14 I 14**	**5'-3'**	no	**34**	**412**
120758758	1,565				5'-3'		17	491
126535602	1,925				5'-3'		22	349
127439823	2,625				5'-3'		36	369
129036324	2,19	ADAMTS19	5'-3'	I 16	5'-3'	no	25	372
130239608	1,575				5'-3'		25	249
137342010	1,85	C5orf5	3'-5'	I 10	5'-3'	ok	20	464
137511093	2,675	BRD8	3'-5'	I 23	5'-3'	ok	34	439
137677115	2,3	CDC25C	3'-5'	I 7	5'-3'	ok	25	424
139580216	2,365	C5orf32	5'-3'	I 2	5'-3'	no	40	357
141724326	2,075				5'-3'		51	409
143200238	2,24				5'-3'		29	342
143476537	1,965				5'-3'		19	431
144044430	2,225				5'-3'		34	529

145191812	1,55	PRELID2	3'-5'	I 1	5'-3'	ok	20	294
149666962	1,965				5'-3'		30	277
153490073	2,38				5'-3'		27	390
154507617	1,88				5'-3'		19	448
154709669	2,025				5'-3'		21	439
155530035	2,23				5'-3'		23	400
157003346	2,545	SOX30	3'-5'	I 3	5'-3'	ok	41	403
157892778	2,19				5'-3'		30	496
158475115	2,265				5'-3'		38	501
158708045	2,77				5'-3'		37	408
158962501	1,785				5'-3'		23	507
161163501	2,19				5'-3'		28	342
162269489	2,755				5'-3'		36	423
163987630	2,39				5'-3'		31	352
164921728	2,66				5'-3'		37	432
165480978	2,645				5'-3'		34	445
170650314	2,33	RANBP17	5'-3'	I 25	5'-3'	no	31	478
172879488	1,97				5'-3'		49	450
174867657	2,04	SFXN1	5'-3'	I 2	5'-3'	no	50	392
179588779	1,43				5'-3'		46	588
289604	1,635	SDHA	5'-3'	E 10 I 10	3'-5'	ok	47	281
2319917	1,935				3'-5'		19	437
4637519	1,84				3'-5'		24	312
8306521	2,56				3'-5'		41	412
9910788	2,245				3'-5'		34	525
10804588	2,065	DAP	3'-5'	I 1	3'-5'	no	19	407
10890811	2,11				3'-5'		28	326
12691502	1,685				3'-5'		41	231
13819968	2,79	DNAH5	3'-5'	I 58	3'-5'	no	36	416
14146729	2,02				3'-5'		28	308
15699292	1,775	FBXL7	5'-3'	I 2	3'-5'	ok	23	309
15902687	2,465	FBXL7	5'-3'	I 2	3'-5'	ok	30	377
16157388	1,92	MARCH11	3'-5'	I 7	3'-5'	no	19	378
17933613	2,015				3'-5'		19	397
19016019	2,13				3'-5'		41	498
22566600	2,5	CDH12	3'-5'	I 1	3'-5'	no	30	384
22754624	2,86	CDH12	3'-5'	I 1	3'-5'	no	35	406
23390106	2,505				3'-5'		28	413
25048485	2,79				3'-5'		34	402
26384154	2,54				3'-5'		30	392

27178387	2,615				3'-5'		32	431
28145494	2,095				3'-5'		44	475
28393679	2,275				3'-5'		44	379
29039183	2,66				3'-5'		31	406
29353554	2,57				3'-5'		30	420
30083501	1,95				3'-5'		21	454
34690496	2,765				3'-5'		37	411
34941122	1,92	RAD1	3'-5'	E 6	3'-5'	no	16	408
36234489	2,165	C5orf33	3'-5'	I 10	3'-5'	no	44	461
39033317	2,075	RICTOR	3'-5'	I 5	3'-5'	no	51	409
41287310	2,1	C6	3'-5'	I 1	3'-5'	no	50	414
52418009	2,53	ITGA2	5'-3'	I 27 E 28	3'-5'	ok	32	448
52999060	2,08	NDUFS4	5'-3'	I 4	3'-5'	ok	49	390
53984200	2,685				3'-5'		32	401
**58550622**	**2,665**	**PDE4D**	**3'-5'**	**I 1**	**3'-5'**	no	**37**	**431**
58674799	2,06	PDE4D	**3'-5'**	I 1	3'-5'	no	46	356
59800654	1,725				3'-5'		31	599
60301423	2,565	NDUFA12L	5'-3'	I 1	3'-5'	ok	31	431
61066566	2,165				3'-5'		45	367
62525090	1,305				3'-5'		19	255
**64913045**	**2,07**	**PPWD1**	**5'-3'**	**I 7**	**3'-5'**	ok	**51**	**408**
66651329	1,755				3'-5'		48	503
68237131	2,105				3'-5'		41	315
68322535	1,86				3'-5'		48	482
69365916	1,655	SERF1A/SMA	5'-3'	I 2/I 10	3'-5'	ok	42	583
70241335	1,655	SERF1A/SMA	5'-3'	I 2/10	3'-5'	ok	42	583
70797623	2,81	BDP1	5'-3'	I 4 E 5 I 5	3'-5'	ok	36	412
71453077	2,015	MAP1B	5'-3'	I 2	3'-5'	ok	52	411
71500000	2,045	MAP1B	5'-3'	I 2	3'-5'	ok	50	425
71739982	2,665				3'-5'		36	441
74679547	2,235	HMGCR	5'-3'	I 6	3'-5'	ok	26	371
78527692	2,865				3'-5'		35	407
82965744	2,03				3'-5'		19	418
84987389	1,61				3'-5'		27	236
85866772	2,465				3'-5'		40	441
86672056	2,2	RASA1	5'-3'	I 5	3'-5'	ok	43	464
86798893	1,365				3'-5'		46	217
87222673	2,375				3'-5'		30	359
89162283	2,4				3'-5'		26	414
92477243	2,51				3'-5'		34	472

92637342	2,74				3'-5'		35	382
93660450	1,88				3'-5'		28	538
97227470	2,65				3'-5'		39	404
99213769	1,675				3'-5'		49	509
99248098	2,335				3'-5'		44	391
99299380	2,455				3'-5'		41	433
100418505	2,87				3'-5'		35	410
101905537	2,39				3'-5'		40	362
101982417	2,045				3'-5'		27	495
103157019	2,43				3'-5'		42	428
105515695	2,435				3'-5'		31	457
105946076	1,78				3'-5'		16	438
107356295	2,095	FBXL17	3'-5'	I 8	3'-5'	no	35	565
109137946	2,025	MAN2A1	5'-3'	I 7 E 8	3'-5'	ok	23	359
114982176	2,29	ICAM2/TMED	3'-5'	I 2/I 2	3'-5'	no	31	332
115264978	1,925	AP3S1	5'-3'	I 5	3'-5'	ok	49	459
**115536097**	**2,36**	**COMMD10**	**5'-3'**	**I 5**	**3'-5'**	ok	**31**	**346**
115684677	2,585				3'-5'		34	361
116594238	2,17				3'-5'		22	398
117148938	2,61				3'-5'		36	366
117713880	1,425				3'-5'		17	299
118419198	2,315				3'-5'		33	501
118893011	2,395	HSD17B4	5'-3'	I 20 E 21 I 21	3'-5'	ok	40	455
119421893	2,155				3'-5'		45	365
120157455	2,225				3'-5'		23	399
120407336	2,33				3'-5'		44	428
122212358	2,21	SNX24	5'-3'	I 1	3'-5'	ok	28	472
122726604	1,95	CCDC100	3'-5'	I 11	3'-5'	no	52	424
123105707	1,62				3'-5'		41	218
124889200	2,18				3'-5'		48	400
127486996	1,105	SLC12A2	5'-3'	I 4	3'-5'	ok	54	245
127828697	2,295	FBN2	3'-5'	I 5	3'-5'	no	43	445
128060236	2,09				3'-5'		22	382
128831676	2,085	ADAMTS19	5'-3'	I 2	3'-5'	ok	22	381
128858497	1,57	ADAMTS19	5'-3'	I 2	3'-5'	ok	52	318
135753737	2,8				3'-5'		34	404
135796999	2,26				3'-5'		46	396
136597569	1,685	SPOCK1	3'-5'	I 3	3'-5'	no	52	477
137786846	1,74	JMJD1B	5'-3'	I 15	3'-5'	ok	16	446
138945977	1,985	UBE2D2	5'-3'	I 1	3'-5'	ok	23	351

	140417722	1,885				3'-5'		50	457
	143170106	1,315				3'-5'		50	571
	143775455	2,54	KCTD16	5'-3'	I 3	3'-5'	ok	30	392
	144208677	1,945				3'-5'		49	455
	**146678649**	**2,365**	**STK32A**	**5'-3'**	**I 4**	**3'-5'**	ok	**43**	**387**
	146778114	2,42	DPYSL3	3'-5'	I 3 E 3 I 2	3'-5'	no	35	318
	146971037	1,73	JAKMIP2	3'-5'	I 20	3'-5'	no	24	528
	147509275	2,295				3'-5'		31	485
	148676374	2,55	AFAP1L1	5'-3'	I 11	3'-5'	ok	41	414
	150945093	2,045				3'-5'		26	333
	150996758	2,38				3'-5'		27	390
	151318313	1,965				3'-5'		29	531
	151588884	1,825				3'-5'		26	289
	**152885578**	**2,695**	**GRIA1**	**5'-3'**	**I 2**	**3'-5'**	ok	**37**	**393**
	153424812	2,25				3'-5'		28	464
	154540788	2,325				3'-5'		24	409
	154556548	1,905				3'-5'		43	523
	154798757	1,9				3'-5'		48	344
	156310120	2,275	TIMD4	3'-5'	I 3	3'-5'	no	25	429
	157699953	2,365				3'-5'		29	451
	159227522	2,035				3'-5'		49	437
	160537843	2,225				3'-5'		29	339
	163586255	2,495				3'-5'		35	485
	163837950	2,07				3'-5'		19	410
	166791345	2,225				3'-5'		41	339
	167067323	2,445	ODZ2	5'-3'	I 1	3'-5'	ok	27	403
	168668384	2,225				3'-5'		39	499
	169163884	1,96	DOCK2	5'-3'	I 26	3'-5'	ok	43	512
	170522316	1,765	RANBP17	5'-3'	I 14	3'-5'	ok	31	591
	173303272	2,525	CPEB4	5'-3'	I 4	3'-5'	ok	31	439
	174145931	2,355				3'-5'		30	463
	175687329	2,84	C5orf25	3'-5'	I 9	3'-5'	no	35	402
	175854470	2,02	UBXD8	5'-3'	I 7	3'-5'	ok	44	328
	179305143	1,88				3'-5'		32	240
**Ø**				**48**			**86**	**33,67**	**411,16**
				**38**			**35**	**34,97**	**410,3**
				**86**			**57**	**32,96**	**411,63**

**6**	3516199	2,405			**I**	5'-3'		30	365
	4290427	1,6				5'-3'		49	294
275 results	6443618	1,69				5'-3'		45	546
	6899507	1,91				5'-3'		37	236
	**7961135**	**2,745**	**XNDC5/MUTE**	**3'-5'**	**I 4**	**5'-3'**	ok	**34**	**425**
	8488497	2,445				5'-3'		41	435
	9871354	2,22				5'-3'		45	378
	10866240	1,405				5'-3'		21	255
	11530992	2,05				5'-3'		49	384
	12443245	1,955				5'-3'		47	473
	13005737	2,67	PHACTR1	5'-3'	I 3	5'-3'	no	36	440
	13846016	2,53				5'-3'		37	458
	14078329	1,855	RNF182	5'-3'	I 1	5'-3'	no	51	453
	17615207	2,375	CAP2	5'-3'	I 4 E 5 I 5	5'-3'	no	33	489
	17851579	2,53				5'-3'		33	458
	20371415	1,95				5'-3'		25	494
	21002431	2,53	CDKAL1	5'-3'	I 9	5'-3'	no	34	468
	22822129	2,375				5'-3'		44	419
	23810801	2,21				5'-3'		23	396
	24112716	1,82				5'-3'		24	510
	24264501	2,165				5'-3'		21	411
	27471958	2,115	ZNF391	5'-3'	I 1	5'-3'	no	34	551
	28934599	2,295				5'-3'		40	343
	29559250	2,705				5'-3'		35	375
	30262502	1,935	TRIM26	3'-5'	I 9	5'-3'	ok	21	457
	32444115	2,51	C6orf10	3'-5'	I 3 E 3 I 2	5'-3'	ok	30	386
	41889540	2,595	USP49	3'-5'	I 3	5'-3'	ok	40	415
	42301069	2,43				5'-3'		41	380
	43680298	2,565	POLH	5'-3'	I 7 E 8 I 8	5'-3'	no	29	411
	45863999	2,265				5'-3'		31	327
	46000392	1,935	CLIC5	3'-5'	I 4	5'-3'	ok	26	507
	47667669	2,405	CD2AP	5'-3'	I 11	5'-3'	no	30	453
	47934059	1,37				5'-3'		23	590
	48686427	2,165				5'-3'		34	277
	49048670	2,59				5'-3'		34	362
	49239429	2,655				5'-3'		36	375
	49711312	2,11	RHAG	3'-5'	I 1	5'-3'	ok	21	422
	50132555	1,82				5'-3'		19	358
	50492845	2,2				5'-3'		44	364

50610804	1,76				5'-3'		47	512
52144594	2,395				5'-3'		38	343
53659824	2,035				5'-3'		22	447
53855408	2,72	LRRC1	5'-3'	I 4 E 5 I 5	5'-3'	no	34	388
55140087	1,9				5'-3'		20	454
56381105	2,715				5'-3'		37	397
63337063	2,365				5'-3'		43	431
63817340	1,875				5'-3'		46	319
65887495	2,575				5'-3'		35	469
68306612	2,855				5'-3'		35	413
71727797	2,23				5'-3'		47	418
73362789	2,19				5'-3'		23	392
78379120	2,045				5'-3'		50	425
79651274	2,245	IRAK1BP1	5'-3'	I 1 E 2 I 2	5'-3'	no	23	415
79872093	2,34				5'-3'		45	402
81491501	2,23				5'-3'		43	458
85100055	2,635				5'-3'		32	427
86113795	1,86				5'-3'		50	356
86546973	1,36				5'-3'		20	562
87196126	2,165				5'-3'		26	461
87619027	2,235				5'-3'		45	381
87985786	1,93	ZNF292	5'-3'	I 4	5'-3'	no	50	370
88403392	2,45	ORC3L	5'-3'	I 13	5'-3'	no	32	464
88992913	2,595				5'-3'		32	383
91197503	2,345				5'-3'		45	403
93151898	1,645				5'-3'		29	223
94638265	2,505				5'-3'		31	443
94809603	2,015				5'-3'		45	337
96756054	2,21	FUT9	5'-3'	I 2	5'-3'	no	43	462
97434990	1,65				5'-3'		20	504
98021948	2,235				5'-3'		46	427
99339188	1,56				5'-3'		44	236
102797228	2,59				5'-3'		31	426
102896058	2,62				5'-3'		30	408
105135364	2,305				5'-3'		44	433
105561695	2,595	LIN28B	5'-3'	I 2	5'-3'	no	30	403
105879470	1,745	PREP	3'-5'	I 9	5'-3'	ok	31	223
108821614	2,385	LACE1	5'-3'	I 6	5'-3'	no	41	371
111322569	2,295	AMD1	5'-3'	E 6	5'-3'	no	46	415
111955501	2,055				5'-3'		46	355

112265014	2,03	FYN	3'-5'	I 2	5'-3'	ok	26	488
113752246	1,44				5'-3'		16	312
115331210	2,05				5'-3'		33	554
115768940	1,835				5'-3'		43	281
116181788	1,375				5'-3'		19	549
117522817	2,195				5'-3'		38	303
119688036	1,57	MAN1A1	3'-5'	I 2	5'-3'	ok	17	490
121218613	2,72				5'-3'		33	398
121419935	1,28				5'-3'		21	588
121601964	2,53	C6orf170	3'-5'	E 20 I 19	5'-3'	ok	39	438
125543297	2,28	TPD52L1	5'-3'	I 1	5'-3'	no	27	448
127538963	2,32	RSPO3	5'-3'	I 4	5'-3'	no	46	408
128472353	1,975	PTPRK	3'-5'	I 7	5'-3'	ok	20	379
128684983	2,7	PTPRK	3'-5'	E 3 I 2	5'-3'	ok	38	404
130261850	2,52				5'-3'		29	398
130405283	2,47	L3MBTL3	5'-3'	I 2 E 3 I 3	5'-3'	no	38	460
131517926	2,2	AKAP7	5'-3'	I 1	5'-3'	no	48	414
132684998	1,905	MOXD1	3'-5'	I 8 E 8	5'-3'	ok	46	325
133304602	1,73				5'-3'		29	578
133520013	1,94				5'-3'		44	312
133904810	2,5				5'-3'		39	444
134122423	2,46				5'-3'		27	406
135534970	2,52				5'-3'		28	408
135672944	2,21	AHI1	3'-5'	I 25	5'-3'	ok	42	346
140245822	2,535				5'-3'		32	447
140746440	2,5				5'-3'		33	354
140764369	2,055				5'-3'		50	423
141525958	2,425				5'-3'		28	389
142534465	2,31	VTA1	5'-3'	I 4	5'-3'	no	43	376
143325623	1,955				5'-3'		17	405
144314785	1,785	PLAGL1	3'-5'	I 4	5'-3'	ok	50	477
145792047	2,62				5'-3'		31	420
146292711	2,485	SHPRH	3'-5'	I 14	5'-3'	ok	28	417
150121656	2,335	PCMT1	5'-3'	I 1	5'-3'	no	28	371
151873518	1,96	C6orf97	5'-3'	I 1	5'-3'	no	21	366
153884724	2,21				5'-3'		22	412
157388255	1,73	ARID1B	5'-3'	I 4	5'-3'	no	17	360
157633045	2,14				5'-3'		31	516
158481031	1,615	SERAC1	3'-5'	I 8 E 8	5'-3'	ok	24	551
160434009	2,26	IGF2R	5'-3'	I 44	5'-3'	no	29	346

160701814	1,65	SLC22A3	5'-3'	I 1	5'-3'	no	23	534
160705012	2,51	SLC22A3	5'-3'	I 1	5'-3'	no	33	356
164589149	1,415				5'-3'		51	541
165382446	2,675				5'-3'		34	439
165714543	1,91	PDE10A	3'-5'	I 18	5'-3'	ok	16	406
166821263	1,81	RPS6KA2	3'-5'	I 11/10	5'-3'	ok	22	326
167861898	2,55				5'-3'		39	384
169192879	2,71				5'-3'		38	406
169354309	2,255				5'-3'		40	335
452784	1,69	EXOC2	3'-5'	I 23	3'-5'	no	18	476
4852416	2,27	CDYL	5'-3'	I 4	3'-5'	ok	31	490
6352624	2,07				3'-5'		40	298
6471692	2,48				3'-5'		30	380
9886180	1,765				3'-5'		29	571
11790353	1,6				3'-5'		19	504
**12144024**	**2,775**	**HIVEP1**	**5'-3'**	**I 2**	**3'-5'**	ok	**37**	**409**
13272825	1,965	PHACTR1	5'-3'	I 5	3'-5'	ok	17	411
14492850	2,29				3'-5'		29	352
14800629	2,495				3'-5'		37	465
14801133	2,69				3'-5'		38	402
16817948	2,55	ATXN1	3'-5'	I 3	3'-5'	no	29	404
19142576	1,95				3'-5'		18	424
20011068	2,26				3'-5'		23	412
25429119	2,195	LRRC16A	5'-3'	I 2	3'-5'	ok	46	383
25447510	1,85	LRRC16A	5'-3'	I 2	3'-5'	ok	49	344
29087841	1,07				3'-5'		16	580
29740127	2,57	MOG	5'-3'	I 2	3'-5'	ok	36	460
32794658	1,855				3'-5'		48	483
33023367	1,555				3'-5'		45	573
34754412	2,515	C6orf106	3'-5'	I 1	3'-5'	no	42	411
45020207	2,225	SUPT3H	3'-5'	I 9	3'-5'	no	29	339
45591490	1,905	RUNX2	5'-3'	I 7	3'-5'	ok	52	433
48000471	2,26	6orf138	3'-5'	I 2	3'-5'	no	25	432
48195426	1,95				3'-5'		23	474
54135522	1,985	C6orf142	5'-3'	I 7	3'-5'	ok	20	437
54689640	1,615				3'-5'		18	327
56484095	1,645	DST	3'-5'	E 54 I 53	3'-5'	no	41	223
56823234	2,475				3'-5'		32	359
57278582	2,245				3'-5'		24	425
62116848	2,09				3'-5'		49	426

62352906	2,645				3'-5'		32	425
63247875	2,255				3'-5'		24	395
63787184	2,285				3'-5'		27	447
64653312	2,135				3'-5'		35	261
65086093	2,27				3'-5'		40	338
65255024	1,945				3'-5'		52	425
65342932	2,235				3'-5'		25	437
65730551	2,475				3'-5'		43	409
66323063	2,745	EGFL11	3'-5'	I 1	3'-5'	no	37	403
67264120	1,67				3'-5'		20	318
67562809	2,395				3'-5'		30	363
68176659	1,39				3'-5'		45	212
68239090	2,685				3'-5'		32	417
68665252	2,35				3'-5'		33	494
71037387	1,59	COL9A1	3'-5'	I 14	3'-5'	no	52	322
72257677	2,115				3'-5'		43	337
72555524	1,97				3'-5'		53	408
72898362	1,41	RIMS1	5'-3'	I 4	3'-5'	ok	21	256
73417831	2,515	KCNQ5	5'-3'	I 1	3'-5'	ok	40	431
73684623	1,985	KCNQ5	5'-3'	I 1	3'-5'	ok	22	361
74182926	1,795	DDX43	5'-3'	I 16	3'-5'	ok	27	545
74493310	1,85	CD109	5'-3'	I 3	3'-5'	ok	46	314
74553064	2,2	CD109	5'-3'	I 20 E 21	3'-5'	ok	30	494
75153452	2,105				3'-5'		26	345
75531205	2,725				3'-5'		32	409
76101859	1,99	FILIP1	3'-5'	I 4	3'-5'	no	48	456
76489227	2,145				3'-5'		28	333
77371852	2,28				3'-5'		25	428
77910239	1,54				3'-5'		54	332
78443791	1,805				3'-5'		33	215
78588206	2,795				3'-5'		34	403
81157482	2,46				3'-5'		34	336
81307077	1,97				3'-5'		34	580
83811951	2,415	UBE2CBP	3'-5'	I 3	3'-5'	no	41	441
**83887231**	**2,11**	**DOPEY1**	**5'-3'**	**I 10**	**3'-5'**	ok	**27**	**336**
**83887231**	**2,11**	**DOPEY1**		**I 10**	**3'-5'**		**27**	**336**
**83887231**	**2,11**	**DOPEY1**		**I 10**	**3'-5'**		**27**	**336**
87011305	1,74				3'-5'		44	272
89115221	2,745				3'-5'		34	425
89307473	2,795				3'-5'		34	415

89321006	2,175				3'-5'		23	389
**90417918**	**2,105**	**MDN1**	**3'-5'**	**I 95**	**3'-5'**	no	**24**	**453**
91374947	2,28				3'-5'		25	428
91869472	2,095				3'-5'		22	383
92393917	2,72				3'-5'		35	378
92536038	2,535				3'-5'		33	361
92840882	1,88				3'-5'		52	438
93956078	1,965				3'-5'		53	411
94463135	1,96				3'-5'		20	376
95188287	2,765				3'-5'		36	397
96585480	2,265	FUT9	5'-3'	I 1	3'-5'	ok	25	387
**96685186**	**2,31**	**FUT9**	5'-3'	**I 2**	**3'-5'**	ok	**24**	**406**
97583394	1,245	KLHL32	5'-3'	I 3	3'-5'	ok	17	555
98102280	1,94				3'-5'		33	242
98743150	2,275				3'-5'		47	409
99268609	2,31				3'-5'		24	412
100296600	2,16				3'-5'		31	512
100464026	1,895				3'-5'		18	383
**101980304**	**1,075**	**GRIK2**	**5'-3'**	**I 1**	**3'-5'**	ok	**16**	**579**
103079067	1,845				3'-5'		18	445
103377192	2,43				3'-5'		29	438
104301810	1,275				3'-5'		50	579
104584938	1,31				3'-5'		18	552
105089529	2,175				3'-5'		36	539
105636730	1,4	LIN28B	5'-3'	E 4	3'-5'	ok	18	534
106089715	2,77				3'-5'		36	420
109065323	1,595	FOXO3	5'-3'	I 1	3'-5'	ok	49	525
109214747	2,09				3'-5'		29	312
109557787	1,73	C6orf182	5'-3'	I 1	3'-5'	ok	50	330
111004605	1,585				3'-5'		52	497
**111765829**	**2,17**	**REV3L**	**3'-5'**	**I 23**	**3'-5'**	no	**23**	**430**
112676158	2,5	LAMA4	3'-5'	I 2	3'-5'	no	31	374
112991552	2,695				3'-5'		32	403
114504272	2,595				3'-5'		38	383
114794458	1,79				3'-5'		49	486
115181284	2,795				3'-5'		34	415
115598927	2,29				3'-5'		37	312
116033813	2,21				3'-5'		45	442
116891556	2,595	FAM26F	5'-3'	E 3	3'-5'	ok	30	403
118573152	2,26	SLC35F1	5'-3'	I 1	3'-5'	ok	23	406

	120974090	2,415				3'-5'		44	411
	121367503	2,05				3'-5'		24	354
	123459246	2,765				3'-5'		36	421
	124687960	2,6	NKAIN2	5'-3'	I 2	3'-5'	ok	30	414
	126984592	2,74				3'-5'		36	392
	127191306	2,825				3'-5'		36	409
	129058053	1,87				3'-5'		23	490
	131888214	2,715				3'-5'		37	421
	132837920	2,145	STX7	3'-5'	I 3 E 3 I 2	3'-5'	no	31	303
	132959554	2,45				3'-5'		27	404
	133440444	1,13				3'-5'		52	588
	134739342	1,445				3'-5'		50	545
	135354464	2,525	HBS1L	3'-5'	I 8	3'-5'	no	36	469
	136604598	2,575	FAM54A	3'-5'	I 4	3'-5'	no	33	449
	138028511	2,285				3'-5'		25	427
	138046142	1,315				3'-5'		47	601
	140371039	2,645				3'-5'		31	415
	140634175	1,895				3'-5'		53	393
	141062349	2,14				3'-5'		22	426
	141605616	2,6				3'-5'		39	424
	141618704	2,225				3'-5'		48	409
	142169185	2,63				3'-5'		33	438
	142214965	1,785				3'-5'		18	361
	142579135	1,9	VTA1	5'-3'	I 7	3'-5'	ok	49	464
	144802435	2,205	UTRN	5'-3'	I 11	3'-5'	ok	26	365
	145953981	2,22				3'-5'		23	398
	148086094	2,14				3'-5'		29	496
	150752594	1,83	IYD	5'-3'	I 2	3'-5'	ok	20	350
	152299797	2,535	ESR1	5'-3'	I 3	3'-5'	ok	29	401
	153858825	1,93				3'-5'		49	458
	154428475	1,875	OPRM1	5'-3'	I 1	3'-5'	ok	54	399
	155246000	2,685				3'-5'		37	427
	162989723	2,76	PARK2	3'-5'	I 1	3'-5'	no	34	396
	163058485	2,505	PARK2	3'-5'	I 1	3'-5'	no	30	385
	163858314	1,695	QKI	5'-3'	I 3	3'-5'	ok	41	233
	165402747	2,32				3'-5'		25	398
	167803421	1,3				3'-5'		47	214
	168837815	2,12				3'-5'		31	298
**Ø**				**57**			94	**33,88**	**411,63**
				**37**			44	**32,72**	**413,78**

				**94**			329	**34,48**	**410,52**
**7**	3894155	1,715	SDK1	5'-3'	I 5	5'-3'	no	24	287
	5014157	2,4				5'-3'		30	454
242 results	7447012	1,88	COL28A1	3'-5'	E 21 I 20	5'-3'	ok	16	400
	8161308	1,795	ICA1	3'-5'	I 8	5'-3'	ok	42	263
	8792971	2,53				5'-3'		29	400
	11483127	2,63	THSD7A	3'-5'	I 7	5'-3'	ok	39	418
	11720560	2,72				5'-3'		33	398
	11902682	2,225				5'-3'		24	429
	13846731	2,45				5'-3'		27	414
	14852134	2,275				5'-3'		23	409
	16623653	2,225	ANKMY2	3'-5'	I 4	5'-3'	ok	48	409
	18983225	2,42	HDAC9	5'-3'	I 25	5'-3'	no	38	348
	19339303	2,03				5'-3'		48	448
	20270104	1,97				5'-3'		21	368
	21728693	2,015	DNAH11	5'-3'	I 44	5'-3'	no	21	377
	22826449	1,67	TOMM7	3'-5'	I 1	5'-3'	ok	19	490
	23078720	2,595				5'-3'		40	415
	23100469	2,135				5'-3'		43	477
	24436057	1,83				5'-3'		27	538
	25682572	2,22				5'-3'		45	378
	25851205	2,235				5'-3'		30	331
	26668305	1,99				5'-3'		36	242
	30532854	2,14				5'-3'		31	516
	35125808	2,325				5'-3'		45	399
	35836804	2,665	SEPT7	5'-3'	I 2	5'-3'	no	37	431
	36201943	2,545	EEPD1	5'-3'	I 2	5'-3'	no	32	445
	38050929	2,02				5'-3'		25	480
	38719951	2,735				5'-3'		35	381
	39083904	2,005	POU6F2	5'-3'	I 1	5'-3'	no	49	375
	40740515	1,74	C7orf10	5'-3'	I 13	5'-3'	no	47	302
	40790778	2,62	C7orf10	5'-3'	I 14	5'-3'	no	38	430
	41001549	2,415				5'-3'		35	501
	42264662	2,055				5'-3'		51	405
	44611796	2,06				5'-3'		20	396
	45359953	2,44				5'-3'		30	446
	46486050	2,645				5'-3'		33	435
	49516605	1,39				5'-3'		18	282
	49737644	1,625				5'-3'		16	469

49757160	1,88				5'-3'		45	310
50901563	2,66				5'-3'		39	406
52980325	2,03				5'-3'		50	428
53231329	2,81				5'-3'		36	406
53386204	2,2				5'-3'		42	474
53389588	1,875				5'-3'		25	509
53688059	2,71				5'-3'		33	422
54590872	2,255	VSTM2A	5'-3'	I 4	5'-3'	no	23	413
63994953	2,045				5'-3'		46	353
65890085	2,56	RABGEF1	5'-3'	I 4	5'-3'	no	41	406
69903764	1,645				5'-3'		48	525
71258633	2,29	CALN1	3'-5'	I 2	5'-3'	ok	33	312
**71408635**	**2,145**	**CALN1**	3'-5'	**I 1**	**5'-3'**	ok	**46**	**445**
71493638	1,625	CALN1	3'-5'	I 2	5'-3'	ok	22	289
72829215	2,63				5'-3'		38	390
72996614	1,85				5'-3'		30	564
81422160	2,62	CACNA2D1	3'-5'	I 38	5'-3'	ok	38	388
83011161	2,225	SEMA3E	3'-5'	I 1	5'-3'	ok	37	299
83363879	2,78				5'-3'		35	390
83862434	2,31				5'-3'		31	482
84147714	1,91				5'-3'		48	346
85101254	2,745				5'-3'		37	403
85482927	2,46				5'-3'		27	412
86107643	2,47				5'-3'		27	410
90192778	2,185	PFTK1	5'-3'	I 1	5'-3'	no	48	417
93451520	2,155				5'-3'		26	355
93523803	1,56				5'-3'		51	512
93649991	1,995				5'-3'		20	383
96989389	2,795				5'-3'		36	415
97224516	2,44				5'-3'		28	392
98415200	2,185	TRRAP	5'-3'	I 56	5'-3'	no	40	321
98962987	2,605	ZKSCAN5	5'-3'	I 6	5'-3'	no	40	413
99001644	2,305	ZNF655	5'-3'	I 2	5'-3'	no	29	463
102571466	2,12	NAPEPLD	3'-5'	I 1	5'-3'	ok	24	450
102639866	2,31				5'-3'		25	396
103606503	1,74	ORC5L	3'-5'	I 8	5'-3'	ok	16	446
106663888	1,93	COG5	3'-5'	I 18 E 18 I 17	5'-3'	ok	50	448
107653310	2,22	NRCAM	3'-5'	I 10 E 10 I 9 E 9	5'-3'	ok	38	510
108792990	2,805				5'-3'		34	413
108964988	2,345				5'-3'		45	415

111198820	2,515	DOCK4	3'-5'	I 35	5'-3'	ok	39	377
112750186	2,255				5'-3'		43	453
114019673	1,92	FOXP2	5'-3'	I 3	5'-3'	no	17	398
114494958	1,74				5'-3'		48	506
114835388	2,26				5'-3'		43	366
114967468	2,13				5'-3'		26	350
115063027	2,445				5'-3'		38	465
115178551	2,63				5'-3'		39	418
115185223	2,425				5'-3'		28	429
115307040	2,205				5'-3'		43	355
115449937	1,76	TFEC	3'-5'	I 1	5'-3'	ok	21	326
115600979	2,19				5'-3'		32	302
115853861	2,18				5'-3'		43	468
117629916	2,295				5'-3'		39	485
118247721	2,485				5'-3'		38	457
118409126	1,855				5'-3'		43	533
119878547	1,995	KCND2	5'-3'	I 1	5'-3'	no	45	333
119954399	1,92	KCND2	5'-3'	I 1	5'-3'	no	18	430
120389884	2,545	ING3	5'-3'	I 4	5'-3'	no	38	445
120570000	1,93	C7orf58	5'-3'	I 16	5'-3'	no	50	370
123565459	2,75				5'-3'		36	394
125233097	2,15				5'-3'		40	504
125592637	2,275				5'-3'		43	449
125853593	1,725				5'-3'		20	329
126611454	2,14	GRM8	3'-5'	I 1	5'-3'	ok	37	536
126795993	1,605				5'-3'		23	275
127014453	2,29				5'-3'		26	382
127070527	2,07				5'-3'		44	480
127979390	2,09				5'-3'		48	382
128662962	1,61	AHCYL2	5'-3'	I 1	5'-3'	no	16	472
130054469	2,42				5'-3'		40	450
132067792	2,39				5'-3'		41	446
136216426	2,59	CHRM2	5'-3'	I 2	5'-3'	no	31	426
137938214	1,91	SVOPL	5'-3'	I 10	5'-3'	no	19	442
140904142	2,57	AGK	5'-3'	I 2	5'-3'	no	32	440
141669282	2,365				5'-3'		45	407
143379883	2,74				5'-3'		33	416
143406897	1,825				5'-3'		39	239
145160586	2,23				5'-3'		47	418
145923696	2,18	CNTNAP2	5'-3'	I 1	5'-3'	no	47	390

147342512	2,41	CNTNAP2	5'-3'	I 15	5'-3'	no	41	442
147403111	1,795	CNTNAP2	5'-3'	I 15	5'-3'	no	43	545
148311712	2,14				5'-3'		29	496
152314046	2,585				5'-3'		31	391
154827537	1,975				5'-3'		44	319
7546702	2,17				3'-5'		40	500
7906457	1,56				3'-5'		17	492
9010129	1,97				3'-5'		42	520
11445406	2,23	THSD7A	3'-5'	I 13	3'-5'	no	30	330
11666764	2,035	THSD7A	3'-5'	I 1	3'-5'	no	21	437
12447669	1,89				3'-5'		17	392
14135353	2,41				3'-5'		40	366
14235974	2,055	DGKB	3'-5'	I 22	3'-5'	no	51	413
14946575	2,2				3'-5'		42	474
17302154	2,13				3'-5'		24	448
18869250	2,48	HDAC9	5'-3'	I 21	3'-5'	ok	33	468
20861899	2,485				3'-5'		34	341
21576770	2,25	DNAH11	5'-3'	I 7	3'-5'	ok	23	414
22630256	2,46				3'-5'		30	376
25796602	2,66				3'-5'		38	396
27663638	2,33	HIBADH	3'-5'	I 1	3'-5'	no	45	400
28426045	2,55	CREB5	5'-3'	I 1	3'-5'	ok	32	374
29348330	2	CHN2	5'-3'	I 1	3'-5'	ok	21	374
31870530	2,205	PDE1C	3'-5'	I 8 E 8	3'-5'	no	45	375
35326189	2,175				3'-5'		48	399
36451677	2,275	ANLN	5'-3'	I 22	3'-5'	ok	29	349
36703270	1,8	AOAH	3'-5'	I 1	3'-5'	no	27	274
40751461	1,865	C7orf10	5'-3'	I 13	3'-5'	ok	54	421
43132299	2,325	HECW1	5'-3'	I 2	3'-5'	ok	29	459
46131531	2,19				3'-5'		24	436
49525437	1,365				3'-5'		21	247
50154471	1,96				3'-5'		18	396
51373157	2,31				3'-5'		46	406
53564335	2,04				3'-5'		24	352
53856529	2,62				3'-5'		30	408
54981079	2,74				3'-5'		36	392
63209464	2,195				3'-5'		44	363
64034824	2,155				3'-5'		44	463
**65898181**	**1,99**	**RABGEF1**	**5'-3'**	**I 5**	**3'-5'**	ok	**19**	**392**
68051773	1,805				3'-5'		32	593

68754502	2,14	AUTS2	5'-3'	I 1	3'-5'	ok	22	392
69068231	2,245	AUTS2	5'-3'	I 2	3'-5'	ok	27	363
72825755	2,49				3'-5'		38	456
76068594	2,2				3'-5'		28	474
77826722	2,665	MAGI2	3'-5'	I 7	3'-5'	no	39	411
78194820	1,785	MAGI2	3'-5'	I 2	3'-5'	no	46	301
78541467	2,26	MAGI2	3'-5'	I 1	3'-5'	no	28	356
78811475	1,845	MAGI2	3'-5'	I 1	3'-5'	no	52	445
79820335	2,275				3'-5'		39	489
79935856	2,43	GNAT3	3'-5'	I 5	3'-5'	no	31	458
80208270	2,49				3'-5'		29	426
80249164	2,72	SEMA3C	3'-5'	I 12	3'-5'	no	38	410
80283250	2,465	SEMA3C	3'-5'	I 5	3'-5'	no	41	431
80801489	2,34				3'-5'		36	506
81020022	2,645				3'-5'		33	435
81303693	2,66				3'-5'		39	412
81754995	1,36	CACNA2D1	3'-5'	I 3	3'-5'	no	49	572
82445316	2,42	PCLO	3'-5'	I 4	3'-5'	no	43	420
83615579	2,205	SEMA3A	3'-5'	I 1	3'-5'	no	37	523
84543881	2,01	SEMA3D	3'-5'	I 3	3'-5'	no	50	432
85752296	2,485				3'-5'		42	417
86847703	2,485	CROT	5'-3'	I 10	3'-5'	ok	39	447
88002078	2,72				3'-5'		33	420
88169164	2,13				3'-5'		21	418
88207834	2,735				3'-5'		37	417
88259758	1,825	ZNF804B	5'-3'	I 1	3'-5'	ok	46	309
88469710	2,395	ZNF804B	5'-3'	I 1	3'-5'	ok	44	403
89038181	2,455				3'-5'		43	413
90334832	2,505	PFTK1	5'-3'	I 5	3'-5'	ok	37	355
90400402	1,935	PFTK1	5'-3'	I 7	3'-5'	ok	22	467
90456997	2,78	PFTK1	5'-3'	I 9	3'-5'	ok	34	400
91413617	1,905	AKAP9	5'-3'	I 1	3'-5'	ok	52	433
91867611	2,52	ANKIB1	5'-3'	E 21	3'-5'	ok	39	440
92028917	2,155	FAM133B	3'-5'	E 12	3'-5'	no	23	433
92316671	2,43				3'-5'		30	448
93633924	1,935				3'-5'		48	467
95314747	2,72	DYNC1I1	5'-3'	I 5	3'-5'	ok	38	408
98068372	1,87				3'-5'		54	420
102363594	1,755	BXL13/LRRC1	3'-5'	I 8/I 2	3'-5'	no	29	245
103333813	2,085	RELN	3'-5'	I 2	3'-5'	no	50	401

109170196	1,635				3'-5'		52	487
109305702	2,045				3'-5'		51	403
109445485	1,96				3'-5'		21	366
110328712	2,09	IMMP2L	3'-5'	I 4	3'-5'	no	47	372
111796000	2,025				3'-5'		19	419
112704789	2,05				3'-5'		44	334
114246946	2,41				3'-5'		37	336
114810440	2,095				3'-5'		47	445
116532325	2,44	ST7	5'-3'	I 2	3'-5'	ok	41	382
117681806	1,85				3'-5'		18	444
117705857	1,84				3'-5'		49	342
117845600	2,21				3'-5'		48	412
119214873	2,53				3'-5'		40	428
119215189	2,575				3'-5'		31	389
120209602	2,505				3'-5'		36	345
120605089	2,765	C7orf58	5'-3'	I 16	3'-5'	ok	33	411
122008221	2,4	CADPS2	3'-5'	I 8 E 8 I 7	3'-5'	no	26	404
122980923	2,065	NDUFA5	3'-5'	I 2	3'-5'	no	26	337
122985394	1,925				3'-5'		42	529
123386014	2,42	SPAM1	5'-3'	I 4	3'-5'	ok	34	490
124579967	2,425				3'-5'		30	369
125044598	2,705				3'-5'		35	443
125271830	2,36				3'-5'		25	412
125990676	2,72	GRM8	3'-5'	I 7	3'-5'	no	38	408
126273743	2,435	GRM8	3'-5'	I 5	3'-5'	no	29	381
126568989	2,635	GRM8	3'-5'	I 1	3'-5'	no	31	417
130028851	2,17				3'-5'		38	298
130179629	1,86				3'-5'		53	386
130822956	1,765	MKLN1	5'-3'	E 18	3'-5'	ok	23	511
133319789	1,985	EXOC4	5'-3'	I 14	3'-5'	ok	31	547
133531692	1,555	LRGUK	5'-3'	I 12 E 13	3'-5'	ok	51	305
134711679	2,3	CNOT4	3'-5'	I 10	3'-5'	no	43	374
135173524	2,175				3'-5'		29	329
135280697	2,435	MTPN	3'-5'	I 3	3'-5'	no	30	371
135520875	1,475				3'-5'		52	299
135619502	2,565				3'-5'		35	347
137522761	2,39				3'-5'		35	506
140752430	2,015				3'-5'		24	347
144640173	2,37				3'-5'		45	408
144805678	2,505				3'-5'		28	413

	146255044	2,135	CNTNAP2	5'-3'	I 3	3'-5'	ok	46	371
	150111546	2				3'-5'		18	404
	156424472	2,39				3'-5'		44	402
	156717148	1,91	UBE3C	5'-3'	I 18	3'-5'	ok	50	366
**Ø**				**53**			**98**	**35,37**	**410,14**
				**45**			**54**	**35,34**	**403,56**
				**98**			**44**	**35,39**	**414,63**
**8**	145721	1,585			I	5'-3'		27	231
	2994691	1,255	CSMD1	3'-5'	I 41	5'-3'	ok	19	573
192 results	3636460	2,16	CSMD1	3'-5'	I 5	5'-3'	ok	24	442
	3996122	1,575	CSMD1	3'-5'	I 3	5'-3'	ok	19	309
	**4254167**	**2,125**	**CSMD1**	3'-5'	**I 3**	**5'-3'**	ok	**50**	**409**
	7706974	1,705	DEFB107B	3'-5'	I 1	5'-3'	ok	16	365
	9673104	2,66	TNKS	5'-3'	E 27	5'-3'	no	36	376
	10879086	2,43	XKR6	3'-5'	I 1	5'-3'	ok	31	360
	12800207	2,215				5'-3'		38	511
	14158066	2,27	SGCZ	3'-5'	I 3	5'-3'	ok	47	408
	14158528	2,12	SGCZ	3'-5'	I 3	5'-3'	ok	44	348
	14615761	2,68	SGCZ	3'-5'	I 1	5'-3'	ok	32	418
	15523857	2,195	TUSC3	5'-3'	I 1	5'-3'	no	40	323
	16168367	2,725				5'-3'		36	389
	16461176	2,675				5'-3'		34	379
	18793665	2,19	PSD3	3'-5'	I 2	5'-3'	ok	43	352
	21347343	1,475				5'-3'		48	259
	24708280	1,895				5'-3'		41	545
	25348968	2,575	KCTD9	3'-5'	I 8	5'-3'	ok	31	429
	30582791	2,03	GTF2E2	3'-5'	I 6	5'-3'	ok	21	438
	30990177	2,015	PURG	3'-5'	I 1	5'-3'	ok	40	287
	32947403	1,88				5'-3'		48	340
	35995078	2,32				5'-3'		45	398
	36569565	2,82				5'-3'		36	410
	36763427	2,34	KCNU1	5'-3'	I 1 E 2	5'-3'	no	27	382
	38343638	2,8	WHSC1L1	3'-5'	I 1	5'-3'	ok	36	404
	38344428	1,94	WHSC1L1	3'-5'	I 1	5'-3'	ok	38	566
	40242910	2,62				5'-3'		37	378
	40310699	1,295				5'-3'		50	575
	40654974	2,635	ZMAT4	3'-5'	I 4	5'-3'	ok	31	401
	41933232	2,42	MYST3	3'-5'	I 12	5'-3'	ok	38	348
	49419337	2,695				5'-3'		38	403

50975561	2,065				5'-3'		43	491
51613328	2,15	SNTG1	5'-3'	I 11	5'-3'	no	22	394
52545633	1,665	PXDNL	3'-5'	I 8	5'-3'	ok	46	541
53401712	1,975	ST18	3'-5'	I 2	5'-3'	ok	51	429
54172319	1,87				5'-3'		44	520
59651788	2,445	SDCBP	5'-3'	I 5	5'-3'	no	42	425
59685606	1,16	NSMAF	3'-5'	I 9	5'-3'	ok	17	572
65775312	2,54	CYP7B1	3'-5'	I 1	5'-3'	ok	31	436
66015062	1,845				5'-3'		27	283
66432453	1,845				5'-3'		19	455
69586985	2,715	C8orf34	5'-3'	I 4	5'-3'	no	33	397
72525576	2,435				5'-3'		43	401
**72562268**	**2,805**				**5'-3'**		**35**	**395**
73293804	2,04				5'-3'		20	392
74666195	2,235	STAU2	3'-5'	I 10	5'-3'	ok	47	417
78365187	2,01				5'-3'		19	396
81517861	2,82				5'-3'		34	408
83432935	2,685				5'-3'		33	391
85505027	2,8	RALYL	5'-3'	I 1	5'-3'	no	36	414
86720416	2,155				5'-3'		49	413
89710562	2,495				5'-3'		32	363
90003569	1,895				5'-3'		16	415
90991596	1,925	OSGIN2	5'-3'	I 2	5'-3'	no	16	409
92492428	2,015				5'-3'		51	397
92936603	2,42				5'-3'		31	358
93214502	1,825				5'-3'		30	569
94500873	2,37				5'-3'		45	410
96747142	2,5				5'-3'		31	444
97814619	2,135	PGCP	5'-3'	I 1	5'-3'	no	21	417
99878568	2,505	STK3	3'-5'	I 1	5'-3'	ok	29	395
100085712	2,26				5'-3'		23	406
100587039	2,615	VPS13B	5'-3'	I 27	5'-3'	no	32	387
101508398	1,69				5'-3'		31	212
102672564	2,48	GRHL2	5'-3'	I 7	5'-3'	no	39	448
106658256	2,555	ZFPM2	5'-3'	I 4	5'-3'	no	29	413
108752081	2,25				5'-3'		31	494
109378251	2,075				5'-3'		23	449
111140465	2,07				5'-3'		20	398
116244474	2,01				5'-3'		50	432
116768989	1,575				5'-3'		18	319

123125505	2,73				5'-3'		33	418
123499281	1,62				5'-3'		27	238
124026303	2,19	ZHX2	5'-3'	I 2	5'-3'	no	41	486
124225995	2,245	WDR67	5'-3'	I 19 E 20 I 20	5'-3'	no	28	353
124512440	1,82	C8orf32	5'-3'	I 3	5'-3'	no	49	480
127138433	1,95				5'-3'		19	434
127309421	2,31				5'-3'		34	306
129834428	2,28				5'-3'		39	330
130487709	1,735				5'-3'		44	271
131975718	1,965	ADCY8	3'-5'	I 7	5'-3'	ok	20	377
132777918	1,29				5'-3'		17	272
136595154	2,465	KHDRBS3	5'-3'	I 1	5'-3'	no	34	481
138155253	2,075				5'-3'		22	379
139391091	2,03	FAM135B	3'-5'	I 3	5'-3'	ok	22	370
683661	2,04				3'-5'		48	446
3634768	1,42	CSMD1	3'-5'	I 5	3'-5'	no	46	228
4714278	2,56	CSMD1	3'-5'	I 1	3'-5'	no	30	396
7353297	1,705	DEFB107B	3'-5'	I 1	3'-5'	no	16	365
11471438	1,525				3'-5'		18	509
13478697	2,135				3'-5'		38	527
13654248	2,62				3'-5'		36	450
14851166	2,06	SGCZ	3'-5'	I 1	3'-5'	no	24	462
14923359	1,33	SGCZ	3'-5'	I 1	3'-5'	no	53	280
15093359	2,01	SGCZ	3'-5'	I 1	3'-5'	no	20	432
16041878	1,88	MSR1	5'-3'	I 8/E 9	3'-5'	ok	47	330
16202692	2,61				3'-5'		40	412
**16998646**	**2,2**	**EFHA2**	**5'-3'**	**I 8**	**3'-5'**	ok	**42**	**474**
20055417	1,605	SLC18A1	3'-5'	I 10	3'-5'	no	53	483
21799486	2,025				3'-5'		21	439
21903439	2,26	XPO7	5'-3'	I 16 E 17 I 17	3'-5'	ok	42	356
23158459	2,185	CHMP7	5'-3'	I 1	3'-5'	ok	47	391
23369452	2,155	ENTPD4	3'-5'	I 2	3'-5'	no	37	533
23919819	1,38				3'-5'		19	548
28879942	2,435	HMBOX1	5'-3'	I 3	3'-5'	ok	43	401
31601266	2,31				3'-5'		46	412
31637496	2,49	NRG1	5'-3'	I 1	3'-5'	ok	41	426
31695349	1,135	NRG1	5'-3'	I 1	3'-5'	ok	51	597
32924712	1,79				3'-5'		37	606
34621760	2,27				3'-5'		47	408
34914252	1,845				3'-5'		43	283

34964210	2,81				3'-5'		36	406
41385620	2,5				3'-5'		28	414
43138909	2,58	HGSNAT	5'-3'	I 7	3'-5'	ok	31	428
48315252	1,805				3'-5'		38	593
50267533	2,485				3'-5'		33	467
52014678	2,525				3'-5'		31	439
52272189	2,445				3'-5'		41	435
53668411	2,32				3'-5'		26	388
57567080	2,695				3'-5'		38	415
57936047	2,47				3'-5'		28	420
59399911	2,54				3'-5'		41	402
60355632	1,205				3'-5'		49	603
62016230	1,945				3'-5'		29	535
62477512	2,79	RLBP1L1	5'-3'	I 3	3'-5'	ok	35	426
63279494	2,42				3'-5'		42	430
64056603	2,315	NKAIN3	5'-3'	I 5	3'-5'	ok	29	461
65226781	2,015				3'-5'		22	451
67095943	1,915	DNAJC5B	5'-3'	E 1 I 1	3'-5'	ok	27	521
67125084	1,755	DNAJC5B	5'-3'	I 2	3'-5'	ok	53	365
68204089	1,89	CSPP1	5'-3'	I 11	3'-5'	ok	17	392
69697133	2,285	C8orf34	5'-3'	I 6	3'-5'	ok	39	487
70087876	2,565				3'-5'		33	367
70513327	2,43				3'-5'		40	448
71272755	2,22	NCOA2	3'-5'	I 4	3'-5'	no	22	410
72511767	2,58				3'-5'		32	380
75421353	2,22				3'-5'		26	450
78240257	2,74				3'-5'		36	392
78358001	1,91				3'-5'		51	376
79307360	1,68				3'-5'		22	300
79350611	1,525				3'-5'		16	329
83996126	2,555				3'-5'		30	395
86915088	2,055				3'-5'		28	503
91186620	2,345				3'-5'		44	393
93707543	1,975				3'-5'		36	239
95441791	1,58				3'-5'		52	320
95934700	2,675	INTS8	5'-3'	I 13 E 14	3'-5'	ok	32	419
96166900	2,815				3'-5'		36	407
100207249	1,95	VPS13B	5'-3'	I 8	3'-5'	ok	49	454
100537799	2,185	VPS13B	5'-3'	I 23	3'-5'	ok	32	517
101024360	2,31				3'-5'		45	422

101169004	1,88	RGS22	3'-5'	I 3	3'-5'	no	48	340
101594554	2,215				3'-5'		27	461
102654099	2,335	GRHL2	5'-3'	I 5	3'-5'	ok	38	487
103045112	2,01	NCALD	3'-5'	I 3	3'-5'	no	51	396
103676611	2,135				3'-5'		21	401
104711158	2,61	RIMS2	5'-3'	I 1	3'-5'	ok	39	396
104847697	2,785	RIMS2	5'-3'	E 3 I 3	3'-5'	ok	34	417
104948341	2,715	RIMS2	5'-3'	I 1	3'-5'	ok	32	411
105164869	2,8	RIMS2	5'-3'	I 17	3'-5'	ok	35	424
106394101	1,735				3'-5'		38	211
106405147	2,58	ZFPM2	5'-3'	I 1	3'-5'	ok	31	428
106698431	2,575	ZFPM2	5'-3'	I 4	3'-5'	ok	31	389
107258046	2,68				3'-5'		33	390
108386475	1,795	ANGPT1	3'-5'	I 4	3'-5'	no	16	435
109688319	1,835				3'-5'		18	371
109979737	2,805				3'-5'		34	405
110955520	2,385				3'-5'		39	467
111484046	2,525				3'-5'		30	429
112512336	1,815				3'-5'		53	377
113233950	2,29				3'-5'		24	402
113366999	1,795	CSMD3	3'-5'	I 56	3'-5'	no	47	505
113789423	2,715	CSMD3	3'-5'	I 12	3'-5'	no	34	431
114696822	1,845				3'-5'		47	323
115048010	2,47				3'-5'		38	358
115066224	2,825				3'-5'		36	409
115680857	1,9				3'-5'		54	414
116059419	2,465				3'-5'		39	367
117584247	2,115				3'-5'		20	407
118037143	2,655				3'-5'		33	433
118960364	2,15				3'-5'		21	414
127288166	2,02				3'-5'		29	520
128183585	2,62				3'-5'		40	408
128365620	2,03				3'-5'		49	380
131686101	1,615				3'-5'		51	501
133773060	1,37				3'-5'		53	288
135095464	1,83				3'-5'		37	598
137192980	2,715				3'-5'		37	421
139721908	1,675	COL22A1	3'-5'	I 47	3'-5'	no	24	539
141874720	2,23	PTK2	3'-5'	I 12	3'-5'	no	30	330
144272187	1,915				3'-5'		36	591

**Ø**				**40**			**81**	**34,61**	**414,18**
				**41**			**32**	**34,52**	**419,02**
				**81**			**49**	**34,68**	**410,65**
**9**	1114054	1,905			I	5'-3'		50	453
	3403675	2,18	RFX3	3'-5'	I 2	5'-3'	ok	45	370
174 results	4350559	2,43				5'-3'		29	438
	7506033	2,405				5'-3'		27	423
	7827749	2,405				5'-3'		28	433
	10502191	2,605	PTPRD	3'-5'	I 1	5'-3'	ok	39	423
	11952627	2,515				5'-3'		29	397
	12139800	2,085				5'-3'		50	417
	14319338	1,835				5'-3'		21	341
	14437575	2,32				5'-3'		32	328
	14472618	2,05				5'-3'		37	264
	15239459	1,405	C9orf52	3'-5'	I 2	5'-3'	ok	17	523
	15441720	2,46	SNAPC3	5'-3'	I 6	5'-3'	no	27	406
	15884578	2,235	C9orf93	5'-3'	I 24	5'-3'	no	23	417
	17853901	1,77				5'-3'		40	580
	18461554	2,625				5'-3'		32	389
	20405622	2,65	MLLT3	3'-5'	I 4	5'-3'	ok	35	364
	20566688	2,1	MLLT3	3'-5'	I 2	5'-3'	ok	27	334
	21576296	1,945				5'-3'		43	515
	22736652	2,73				5'-3'		33	418
	23203999	1,61				5'-3'		19	502
	25044494	2,025				5'-3'		48	449
	27003224	2,09	IFT74	5'-3'	I 10	5'-3'	no	21	392
	27788418	2,465				5'-3'		40	441
	29016817	2,395				5'-3'		27	393
	29145993	2,395				5'-3'		29	373
	29216504	2,325				5'-3'		41	359
	29369701	1,9				5'-3'		30	554
	29467781	2,05				5'-3'		19	404
	38953171	2,845				5'-3'		35	403
	40060698	1,54				5'-3'		25	242
	40369433	1,54				5'-3'		25	242
	42776199	2,475				5'-3'		32	359
	65442096	1,55				5'-3'		25	244
	66677238	2,475				5'-3'		32	359
	67055448	1,875				5'-3'		16	419

69051667	2,525				5'-3'		41	399
69403589	1,875				5'-3'		16	419
69418347	2,525				5'-3'		41	399
69604014	1,875				5'-3'		16	419
69618816	2,525				5'-3'		41	399
71007276	2,405	TJP2	5'-3'	I 1	5'-3'	no	39	355
73089464	2,05				5'-3'		51	414
76213666	2,05				5'-3'		24	354
76799856	2,335	C9orf41	3'-5'	I 6	5'-3'	ok	29	361
77956407	2,66	PCSK5	5'-3'	I 10	5'-3'	no	34	376
78561015	2,18	.OC10012976	3'-5'	I 6	5'-3'		31	310
79235495	2,16	GNA14	3'-5'	I 4 E 4	5'-3'	ok	43	346
79682341	2,47	GNAQ	3'-5'	I 2	5'-3'	ok	30	378
82078582	1,325				5'-3'		18	269
85688749	2,17	KIF27	3'-5'	E 10 I 9	5'-3'	ok	21	410
86747824	1,77	NTRK2	5'-3'	I 16	5'-3'	no	28	258
89696425	1,935				5'-3'		17	417
92060132	2,695				5'-3'		33	393
92999013	1,66				5'-3'		19	326
93882968	2,185	SPTLC1	3'-5'	E 4 I 3	5'-3'	ok	45	371
101527530	1,99				5'-3'		47	466
**101599087**	**1,825**				**5'-3'**		**22**	**489**
101604377	2,275				5'-3'		25	429
101911009	1,77	INVS	5'-3'	I 2	5'-3'	no	50	480
103608597	2,09				5'-3'		47	372
104698084	1,85				5'-3'		16	424
106059979	2,255				5'-3'		46	423
106535916	2,38				5'-3'		40	458
107421534	2,655	FKTN	5'-3'	I 8 E 9	5'-3'	no	31	413
111964648	1,91	M2-AKAP2/AK	5'-3'	I 3/10	5'-3'	no	22	472
113478510	2,03				5'-3'		22	370
117472515	1,935				5'-3'		51	381
119377210	1,955				5'-3'		26	315
121510669	1,86				5'-3'		24	502
**123107513**	**1,575**	**GSN**	**5'-3'**	**I 3/10**	**5'-3'**	no	**26**	**239**
126364429	2,225	NR6A1	3'-5'	I 2	5'-3'	ok	46	389
127049249	2,235				5'-3'		24	391
128951062	1,91	RALGPS1	5'-3'	I 8	5'-3'	no	21	356
134780906	2,67	TSC1	3'-5'	I 8	5'-3'	ok	39	408
137860831	2,45	CAMSAP1	3'-5'	I 5	5'-3'	ok	29	434

139483822	2,54	PNPLA7	3'-5'	I 24	5'-3'	ok	32	372
**2079221**	**1,84**	**SMARCA2**	**5'-3'**	**I 19**	**3'-5'**	ok	**39**	**242**
2917065	2,54				3'-5'		30	392
3554834	2,36				3'-5'		42	376
7569864	2,025				3'-5'		50	389
9664089	2,19	PTPRD	3'-5'	I 6	3'-5'	no	38	516
10284392	1,89	PTPRD	3'-5'	I 2	3'-5'	no	53	392
12358905	2,76				3'-5'		33	406
12982316	2,65				3'-5'		33	384
13173537	2,265	MPDZ	3'-5'	E 18 I 17	3'-5'	no	30	337
13629788	1,29				3'-5'		22	222
13769636	1,785				3'-5'		50	341
14033317	1,86				3'-5'		22	336
14842014	1,5	FREM1	3'-5'	I 6	3'-5'	no	54	324
15378579	2,48				3'-5'		38	458
15761052	2,515	C9orf93	5'-3'	I 18	3'-5'	ok	36	471
17048785	1,34				3'-5'		21	242
17172935	2,385	CNTLN	5'-3'	I 2	3'-5'	ok	36	321
17381144	2,28	CNTLN	5'-3'	I 10	3'-5'	ok	30	340
17622917	1,565	SH3GL2	5'-3'	I 1	3'-5'	ok	24	561
20461373	2,495	MLLT3	3'-5'	I 2	3'-5'	no	29	393
21317043	1,95				3'-5'		18	394
22433669	2,13				3'-5'		21	418
23210172	2,19				3'-5'		24	382
25070421	2,29				3'-5'		28	362
27279134	1,905	C9orf11	3'-5'	I 6 E 6 I 5	3'-5'	no	19	375
27958209	2,12	LINGO2	3'-5'	I 6	3'-5'	no	50	408
28634566	2,76	LINGO2	3'-5'	I 2	3'-5'	no	36	396
28787541	2,29				3'-5'		25	392
28852489	2,16				3'-5'		26	462
28956026	2,235				3'-5'		47	401
29080422	2,44				3'-5'		27	416
31247255	1,975				3'-5'		17	409
31681105	2,47				3'-5'		28	420
35187421	1,475	UNC13B	5'-3'	I 1	3'-5'	ok	52	519
37243512	1,735	ZCCHC7	5'-3'	I 2	3'-5'	ok	16	371
39021404	1,555				3'-5'		25	245
42757008	2,525				3'-5'		41	399
42771810	1,875				3'-5'		16	419
47048421	1,55				3'-5'		25	244

66658083	2,525				3'-5'		41	399
66672849	1,875				3'-5'		16	419
67051065	2,475				3'-5'		32	359
69032524	2,475				3'-5'		32	359
69399202	2,475				3'-5'		32	359
69599625	2,475				3'-5'		32	359
70323513	2,77	PGM5	5'-3'	I 10	3'-5'	ok	36	398
71445743	2,365	APBA1	3'-5'	I 1	3'-5'	no	25	411
71800377	1,555				3'-5'		23	553
72859039	2,48	TRPM3	3'-5'	I 1	3'-5'	no	39	448
75522739	1,85				3'-5'		20	464
75853811	1,59				3'-5'		43	586
76085311	2,41				3'-5'		43	422
76141860	2,315				3'-5'		25	421
76796997	2,345	C9orf41	3'-5'	I 6	3'-5'	no	36	313
77786815	1,745	PCSK5	5'-3'	I 2	3'-5'	ok	18	353
79804030	2,37	GNAQ	3'-5'	I 1	3'-5'	no	45	410
81211046	1,955				3'-5'		50	443
81964294	2,58				3'-5'		32	438
87649957	2,165				3'-5'		21	407
87695482	1,86				3'-5'		52	376
88367805	2,02				3'-5'		47	460
94182280	2,02	CENPP	5'-3'	I 5	3'-5'	ok	30	288
97276074	2,27	PTCH1	3'-5'	I 12	3'-5'	no	47	408
97682127	2,51	C9orf102	5'-3'	I 1	3'-5'	ok	32	452
99041042	2,505	KIAA1529	5'-3'	I 1	3'-5'	ok	30	385
99530799	2,61				3'-5'		31	396
101040078	2,74				3'-5'		33	416
101593942	1,915				3'-5'		46	327
102805286	1,635				3'-5'		19	497
103171245	1,77	BAAT	3'-5'	I 2	3'-5'	no	52	358
103535572	2,69	GRIN3A	3'-5'	I 1	3'-5'	no	37	426
103940080	2,17				3'-5'		40	318
104987977	2,65				3'-5'		39	404
105053525	2,69				3'-5'		35	446
105535927	2,2				3'-5'		26	364
105712931	1,56				3'-5'		22	542
108092039	2,38				3'-5'		26	400
108092118	2,375				3'-5'		32	479
108793228	2,47	ZNF462	5'-3'	I 10	3'-5'	ok	27	408

	109747481	2,715				3'-5'		32	407
	111099694	2,245	EPB41L4B	3'-5'	I 1	3'-5'	no	40	333
	111322215	2,445				3'-5'		36	333
	112947515	2,1				3'-5'		39	524
	113350228	1,42	ZNF483	5'-3'	I 5	3'-5'	ok	50	550
	114094817	2,12	ROD1	3'-5'	I 3	3'-5'	no	20	410
	116349297	2,53				3'-5'		31	380
	**116908622**	**2,63**	**TNC**	**3'-5'**	**I 1**	**3'-5'**	no	**31**	**418**
	117245745	2,415				3'-5'		26	407
	117986996	1,845	PAPPA	5'-3'	I 1	3'-5'	ok	17	383
	118468789	2,475	ASTN2	3'-5'	I 15	3'-5'	no	27	409
	122998881	2,31	RAB14	3'-5'	I 1	3'-5'	no	27	442
	124685152	2,605	RC3H2	3'-5'	I 3 E 3 I 2	3'-5'	no	34	365
	126168022	2,61	PSMB7	3'-5'	I 6	3'-5'	no	40	412
	127100717	1,835	GAPVD1	5'-3'	I 1 E 2 I 2	3'-5'	ok	54	391
	128058838	2,585				3'-5'		40	417
	135884861	1,815				3'-5'		30	247
	139791199	1,5	EHMT1	5'-3'	I 11	3'-5'	ok	53	314
**Ø**				**28**			**64**	**32,49**	**395,47**
				**37**			**33**	**34,14**	**390,89**
				**65**			**31**	**31,5**	**398,2**
**10**	2012380	2,51			I	5'-3'		29	396
	5155798	2,605				5'-3'		34	365
161 results	6784500	1,895				5'-3'		21	465
	7390019	2,695	SFMBT2	3'-5'	I 4	5'-3'	ok	34	383
	7845687	1,8	KIN	3'-5'	E 10 I 9	5'-3'	ok	42	554
	10489122	1,9				5'-3'		17	424
	10693280	2,355				5'-3'		32	483
	10718685	1,915				5'-3'		16	407
	11641840	1,84				5'-3'		51	456
	12356991	1,425				5'-3'		49	559
	13154135	1,41				5'-3'		18	532
	13990885	2,61	FRMD4A	3'-5'	I 2	5'-3'	ok	39	396
	16383844	2,155				5'-3'		40	315
	21307974	2,275	NEBL	3'-5'	I 3	5'-3'	ok	47	409
	21998722	2,285	MLLT10	5'-3'	I 8	5'-3'	no	45	391
	22813198	1,735				5'-3'		51	341
	**22959275**	**1,905**	**PIP5K2A**	**3'-5'**	**I 1**	**5'-3'**	ok	**17**	**395**
	24123668	2,315	KIAA1217	5'-3'	I 1	5'-3'	no	44	387

28459746	2,365	MPP7	3'-5'	I 8	5'-3'	ok	28	441
29865066	2,34	SVIL	3'-5'	I 8/6	5'-3'	ok	42	446
30673990	1,66	PAPD1	3'-5'	I 1	5'-3'	ok	47	286
33822242	2,555				5'-3'		38	375
36368859	1,875				5'-3'		18	439
39092542	1,875				5'-3'		16	419
39107317	2,55				5'-3'		41	404
42036248	2,48				5'-3'		32	360
42526240	1,885				5'-3'		42	281
45048672	2,66				5'-3'		36	376
46339057	1,91				5'-3'		50	452
46486713	2,545				5'-3'		32	445
46821130	1,91				5'-3'		50	452
47033658	2,23				5'-3'		43	458
53276279	1,475	PRKG1	5'-3'	I 4	5'-3'	no	49	269
53925543	1,76				5'-3'		17	366
54055714	1,83				5'-3'		26	528
57165862	2,37				5'-3'		26	398
57806165	2,42				5'-3'		43	398
58326930	1,945				5'-3'		17	415
59044911	1,92				5'-3'		17	398
60146045	2,12	BICC1	5'-3'	I 3	5'-3'	no	23	440
62296973	2,055				5'-3'		31	533
64954974	2,66	REEP3	5'-3'	I 1	5'-3'	no	34	376
68393366	2,175	TNNA3/LRRTI	3'-5'	I 7/I 2	5'-3'	ok	49	409
70741965	1,99	HK1	5'-3'	I 4	5'-3'	no	24	342
70808592	2,795	HK1	5'-3'	I 8	5'-3'	no	36	403
72689427	2,38	UNC5B	5'-3'	I 1	5'-3'	no	36	498
74298651	1,43	CCDC109A	5'-3'	I 5	5'-3'	no	19	280
75725879	1,98	ADK	5'-3'	I 3	5'-3'	no	47	468
78162663	1,99				5'-3'		18	416
81411382	1,98				5'-3'		24	340
84082670	1,725	NRG3	5'-3'	I 1	5'-3'	no	24	529
85605471	2,295				5'-3'		24	415
87231906	2,4				5'-3'		39	464
88479256	2,18	LDB3	5'-3'	I 12	5'-3'	no	45	448
88921094	1,93	FAM35A	5'-3'	I 6	5'-3'	no	50	448
90635199	2,745	STAMPL1		I 1	5'-3'		33	403
91274996	2,375	SLC16A12	3'-5'	I 2	5'-3'	ok	26	399
93356200	1,84				5'-3'		45	302

93535085	1,85				5'-3'		16	424
100831953	2,33	HPSE2	3'-5'	I 3	5'-3'	ok	45	400
101514834	2,42				5'-3'		34	490
109222680	2,27				5'-3'		25	430
109270658	1,66				5'-3'		25	552
109592077	2,355				5'-3'		35	513
112773594	1,185				5'-3'		19	231
112812401	2,47				5'-3'		41	430
114732212	2,25	TCF7L2	5'-3'	I 3	5'-3'	no	41	344
116823873	2,37				5'-3'		27	388
117358892	2,175	ATRNL1	5'-3'	I 26	5'-3'	no	29	329
117558607	2,35	ATRNL1	5'-3'	I 27	5'-3'	no	25	404
119573380	1,84				5'-3'		16	426
127319428	2,425				5'-3'		43	399
129403487	2,175				5'-3'		34	539
274329	1,525	ZMYND11	5'-3'	I 6	3'-5'	ok	45	239
1146605	1,59	WDR37	5'-3'	I 11	3'-5'	ok	48	282
2971606	1,92				3'-5'		22	348
4257587	1,79				3'-5'		48	496
4390211	2,715				3'-5'		38	407
4548344	1,57				3'-5'		20	520
4816949	2,545				3'-5'		41	415
4830641	2,565				3'-5'		31	387
5831797	2,555	C10orf18	5'-3'	E 17 I 17	3'-5'	ok	35	473
6116917	2,095	IL2RA	3'-5'	I 1	3'-5'	no	34	555
6904131	2,025				3'-5'		19	419
8054578	2,225	TAF3	5'-3'	I 6	3'-5'	ok	25	439
9582193	2,395				3'-5'		44	415
10171791	2,49				3'-5'		28	416
11055433	1,47				3'-5'		25	590
11678392	2,095				3'-5'		23	373
12081666	1,755	UPF2	3'-5'	I 7 E 7 I 6	3'-5'	no	54	443
13552254	2,45	C10orf30	3'-5'	I 6	3'-5'	no	43	414
14046566	2,135	FRMD4A	3'-5'	I 2	3'-5'	no	34	547
15883491	2,465	C10orf97	3'-5'	I 10	3'-5'	no	39	451
16049256	1,92				3'-5'		43	298
16575241	2,655	PTER	5'-3'	I 4	3'-5'	ok	32	395
17205560	2,645	CUBN	3'-5'	I 5 E 5 I 4	3'-5'	no	32	425
18770286	2,145	CACNB2	5'-3'	I 2	3'-5'	ok	23	383
20509415	2,38	PLXDC2	5'-3'	I 9	3'-5'	ok	27	428

20635333	1,87				3'-5'		52	378
26135704	1,72				3'-5'		17	460
26698875	1,35				3'-5'		50	564
27207584	2,24				3'-5'		23	402
27866538	1,98	RAB18	5'-3'	I 6 E 7	3'-5'	ok	52	400
27968156	2,165				3'-5'		49	411
28445435	2,17	MPP7	3'-5'	I 13	3'-5'	no	45	450
29145934	2,27				3'-5'		45	430
30081319	2,73				3'-5'		33	418
31176647	1,81	ZNF438	3'-5'	I 6	3'-5'	no	20	346
32147496	2,06	ARHGAP12	3'-5'	I 12	3'-5'	no	20	422
32419982	1,26				3'-5'		50	236
32754818	2,015				3'-5'		24	471
36072038	2,245				3'-5'		23	415
42013103	2,55				3'-5'		41	404
43318230	2,42				3'-5'		39	358
43443241	2,51				3'-5'		28	406
56001397	2,495	PCDH15	3'-5'	I 2	3'-5'	no	40	383
57069879	2,195				3'-5'		22	403
57792504	2,555				3'-5'		41	405
58746143	2,45				3'-5'		29	434
58956887	2,47				3'-5'		41	430
59526269	1,745				3'-5'		16	373
60631266	2,6	PHYHIPL	5'-3'	I 1	3'-5'	ok	36	364
60864619	2,57				3'-5'		30	420
65248141	1,56				3'-5'		18	502
65979101	2,675				3'-5'		35	449
67315942	2,04				3'-5'		31	282
70334098	2,135	DDX50	5'-3'	I 1	3'-5'	ok	27	477
73177016	1,9	CDH23	5'-3'	I 38	3'-5'	ok	38	574
77184697	2,505				3'-5'		41	423
77244716	2,245	C10orf11	5'-3'	I 1	3'-5'	ok	30	333
78314853	2,56	KCNMA1	3'-5'	I 28/E 27	3'-5'	no	31	386
79628906	2,515				3'-5'		34	471
80829550	1,905	C10orf56	3'-5'	I 2	3'-5'	no	35	215
83073476	1,805				3'-5'		17	375
84285651	1,71	NRG3	5'-3'	I 2	3'-5'	ok	43	562
84774059	2,505				3'-5'		36	345
85689186	2,08				3'-5'		28	320
87022516	1,96				3'-5'		52	422

	92870581	2,14				3'-5'		48	426
	93440242	1,91				3'-5'		46	326
	97896042	1,545	ZNF518	3'-5'	I 3	3'-5'	no	48	273
	100923606	2,135	HPSE2	3'-5'	I 2	3'-5'	no	47	381
	104008940	2,185	GBF1	5'-3'	I 2	3'-5'	ok	30	497
	104630450	2,295	AS3MT	5'-3'	I 9	3'-5'	ok	46	403
	104762935	1,445				3'-5'		17	515
	106248931	2,615				3'-5'		39	397
	108127194	2,555				3'-5'		33	365
	108833039	1,98	SORCS1	3'-5'	I 1	3'-5'	no	52	418
	110558477	1,55				3'-5'		54	484
	114922411	2,33				3'-5'		45	400
	115989745	2,51	VWA2	5'-3'	I 1	3'-5'	ok	34	346
	117082957	1,775	ATRNL1	5'-3'	I 18	3'-5'	ok	44	279
	117419100	2	ATRNL1	5'-3'	I 26	3'-5'	ok	25	334
	119516861	2,29				3'-5'		30	476
	120784810	2,485	EIF3A	3'-5'	E 22	3'-5'	no	29	391
	121300734	1,765				3'-5'		54	441
	123669486	2,48	ATE1	3'-5'	I 3	3'-5'	no	28	400
	126230823	1,675	LHPP	5'-3'	I 6	3'-5'	ok	41	589
	129646191	2,1	PTPRE	5'-3'	I 1	3'-5'	ok	20	404
	130706147	1,985				3'-5'		52	401
	131128728	2,325				3'-5'		46	409
**Ø**				**36**			**64**	**34,42**	**413,09**
				**28**			**33**	**36,18**	**405,82**
				**64**			**31**	**33,22**	**418,02**
**11**	3329909	2,31			I	5'-3'		24	406
	4315334	2,72				5'-3'		34	430
174 results	4871137	2,525				5'-3'		42	409
	5476227	2,345				5'-3'		43	435
	10069570	2,425	SBF2	3'-5'	I 2	5'-3'	ok	28	429
	11546522	1,81	GALNTL4	3'-5'	I 1	5'-3'	ok	16	386
	15332396	2,14				5'-3'		22	426
	15830553	2,555				5'-3'		41	405
	20939375	2,625	NELL1	5'-3'	I 12	5'-3'	no	40	409
	21010443	2,455	NELL1	5'-3'	I 12	5'-3'	no	27	413
	21139083	1,595	NELL1	5'-3'	I 13	5'-3'	no	26	243
	21384594	2,58	NELL1	5'-3'	I 15	5'-3'	no	33	370
	22065457	1,87				5'-3'		30	560

22513734	2,665				5'-3'		38	397
24305369	2,49				5'-3'		30	382
24679949	2,395	LUZP2	5'-3'	I 1	5'-3'	no	42	435
26304748	2,01				5'-3'		38	552
26701545	2,675				5'-3'		38	419
27592847	2,825				5'-3'		36	409
29341283	2,505				5'-3'		30	385
30219087	1,995				5'-3'		35	585
30548365	2,245	MPPED2	3'-5'	I 1	5'-3'	ok	42	465
31171334	2,35				5'-3'		33	324
32662646	1,47	CCDC73	3'-5'	I 6	5'-3'	ok	19	530
33015529	2,11				5'-3'		50	406
34751384	2,415				5'-3'		42	387
37447167	1,605				5'-3'		20	513
37600576	2,765				5'-3'		33	411
37932775	2,435				5'-3'		42	391
38320924	2,455				5'-3'		31	365
39711398	1,955				5'-3'		19	385
39839042	2,79				5'-3'		35	426
40147628	2,79	LRRC4C	3'-5'	I 1	5'-3'	ok	34	416
41285432	2,695				5'-3'		34	383
42997611	1,98				5'-3'		40	538
43225801	2,615				5'-3'		40	407
43668894	2,135	HSD17B12	5'-3'	I 1	5'-3'	no	42	331
46478614	2,58	FLJ20294	3'-5'	I 10	5'-3'	ok	30	400
46532225	1,67	FLJ20294	3'-5'	I 1	5'-3'	ok	25	550
49329528	1,57				5'-3'		21	288
55572608	2,41				5'-3'		31	356
56279375	2,02				5'-3'		47	460
58380849	2,375				5'-3'		28	439
68408609	2,06				5'-3'		22	376
73070719	2,39	RAB6A	3'-5'	I 6	5'-3'	ok	36	322
74385900	1,835	NEU3	5'-3'	I 2	5'-3'	no	21	341
76276445	2,54	PHCA	5'-3'	I 1	5'-3'	no	38	372
76632382	2,505	GDPD4	3'-5'	E 12 I 11	5'-3'	ok	35	483
76701228	2,71				5'-3'		37	422
78235375	2,045				5'-3'		51	403
79110190	2,525				5'-3'		42	409
84039503	2,03	DLG2	3'-5'	I 1	5'-3'	ok	51	400
84292767	2,44	DLG2	3'-5'	I 1	5'-3'	ok	43	416

85037995	2,145	TMEM126A	5'-3'	I 1	5'-3'	no	46	373
85054268	1,89				5'-3'		21	466
85358601	1,64	PICALM	3'-5'	I 19	5'-3'	ok	46	546
86407014	2,665				5'-3'		39	407
87499587	2,165	RAB38	3'-5'	I 2	5'-3'	ok	25	367
88757903	2,14	NOX4	3'-5'	I 11	5'-3'	ok	40	506
91438756	1,965				5'-3'		27	511
91565846	2,49				5'-3'		36	342
92923661	2,62				5'-3'		39	398
93257673	2,05				5'-3'		50	394
94451893	2,21				5'-3'		41	336
94513530	1,97				5'-3'		46	480
96525490	1,86				5'-3'		17	432
96582809	2,1				5'-3'		29	504
98732831	2,04	CNTN5	5'-3'	I 2	5'-3'	no	46	466
101787296	2,72	TMEM123	3'-5'	I 1	5'-3'	ok	37	398
107773323	1,365	C11orf65	3'-5'	I 7	5'-3'	ok	48	237
108097131	2,37	DDX10	5'-3'	I 12	5'-3'	no	45	408
109352143	2,36				5'-3'		33	326
110556747	1,475				5'-3'		19	529
110712225	2,495				5'-3'		35	333
110890983	2,41	FLJ46266	5'-3'	E 1 I 1	5'-3'	no	44	412
114728073	1,69	CADM1	3'-5'	I 1	5'-3'	ok	16	362
114835794	2,005	CADM1	3'-5'	I 1	5'-3'	ok	51	395
115354267	2,35				5'-3'		31	344
117312795	2,08				5'-3'		22	380
120343863	1,96	GRIK4	5'-3'	I 17	5'-3'	no	42	522
121790045	2,28				5'-3'		46	400
124475413	1,62	LOC219854	3'-5'	I 4	5'-3'		25	560
125402697	2,215	CDON	3'-5'	I 1	5'-3'	ok	48	411
127757166	2,025				5'-3'		23	459
128637073	2,575				5'-3'		29	409
129020800	1,97				5'-3'		39	550
134369689	1,965				5'-3'		20	377
2431776	2,66	KCNQ1	5'-3'	I 1	3'-5'	ok	31	412
4212189	2,275				3'-5'		25	429
4257606	2,27				3'-5'		25	430
4645043	1,97				3'-5'		21	368
5282947	1,705				3'-5'		16	365
5682856	2,715	TRIM22	5'-3'	I 4	3'-5'	ok	38	407

6873737	2,22				3'-5'		33	520
7483238	2,55	OLFML1	5'-3'	I 2	3'-5'	ok	38	374
10161439	2,185	SBF2	3'-5'	I 2	3'-5'	no	22	417
14223005	2,445	SPON1	5'-3'	I 7	3'-5'	ok	40	373
14771531	2,51	PDE3B	5'-3'	I 4	3'-5'	ok	33	356
14836924	2,47	PDE3B	5'-3'	I 12 E 12 I 13	3'-5'	ok	34	338
21208261	1,205	NELL1	5'-3'	I 14	3'-5'	ok	53	255
21292276	2,295	NELL1	5'-3'	I 14	3'-5'	ok	29	465
21757869	1,89				3'-5'		20	456
22760198	2,055	GAS2	5'-3'	I 6	3'-5'	ok	45	345
23931002	2,53				3'-5'		41	418
24517534	2,68	LUZP2	5'-3'	I 1	3'-5'	ok	35	370
**24614496**	**2,555**	**LUZP2**	5'-3'	**I 1**	**3'-5'**	ok	**33**	**453**
25690694	2,765				3'-5'		37	411
26669797	2,205	SLC5A12	3'-5'	E 9	3'-5'	no	24	385
27859482	2,525				3'-5'		42	409
28936711	2,355				3'-5'		45	413
29771340	2,8				3'-5'		36	404
31237007	1,755				3'-5'		23	305
31565196	2,28	ELP4	5'-3'	I 3	3'-5'	ok	44	438
31592354	2,465	ELP4	5'-3'	I 5	3'-5'	ok	27	407
34786532	1,505				3'-5'		25	583
36134119	1,985	LDLRAD3	5'-3'	I 4	3'-5'	ok	52	401
36978710	2,35				3'-5'		27	434
38150838	1,835				3'-5'		30	567
40103175	2,75	LRRC4C	3'-5'	I 1	3'-5'	no	34	424
40744117	2,87				3'-5'		35	408
41350610	1,87				3'-5'		20	358
42161126	1,645				3'-5'		42	233
42759271	2,585				3'-5'		36	361
43953990	2,255				3'-5'		45	385
49300566	2,145				3'-5'		40	313
49898685	1,665				3'-5'		54	357
56914919	2,025				3'-5'		19	419
58672095	1,925	FAM111A	5'-3'	I 2	3'-5'	ok	51	439
58968138	2,14	OR5A1	5'-3'	E1	3'-5'	ok	49	402
59003402	2,2				3'-5'		22	414
61800938	2,07				3'-5'		25	470
62290610	2,375	POLR2G	5'-3'	E 8	3'-5'	ok	38	479
67763485	2,645				3'-5'		36	445

69511891	2,38				3'-5'		36	320
71737045	2,2	CLPB	3'-5'	I 6	3'-5'	no	26	454
81168726	1,53				3'-5'		54	488
81744979	1,225				3'-5'		16	549
83260625	2,255	DLG2	3'-5'	I 11	3'-5'	no	45	433
83270991	2,81	DLG2	3'-5'	I 10	3'-5'	no	36	412
84813552	2,275				3'-5'		45	429
86781319	2,665				3'-5'		32	397
86871203	2,215				3'-5'		33	521
87224089	2,025				3'-5'		18	409
88017168	2,755	GRM5	3'-5'	I 3	3'-5'	no	37	413
88727037	1,715	NOX4	3'-5'	I 13	3'-5'	no	21	317
88848335	2,12	NOX4	3'-5'	I 2	3'-5'	no	21	420
90282439	2,095				3'-5'		22	383
90760326	2,285				3'-5'		45	427
91952156	2,15	FAT3	5'-3'	I 3	3'-5'	ok	47	434
92042982	2,765	FAT3	5'-3'	I 3	3'-5'	ok	34	421
92950947	2,2				3'-5'		48	404
95776063	2,41				3'-5'		28	386
97735652	2,695				3'-5'		33	425
99349980	2,205	CNTN5	5'-3'	I 8	3'-5'	ok	28	473
100831265	2,4	TRPC6	3'-5'	I 10	3'-5'	no	30	364
100883719	1,955	TRPC6	3'-5'	I 1	3'-5'	no	48	355
102711970	1,96	DYNC2H1	5'-3'	I 83	3'-5'	ok	51	432
**102783264**	**2,51**	**DYNC2H1**	5'-3'	**I 85**	**3'-5'**	ok	**41**	**422**
106297558	2,39	GUCY1A2	3'-5'	I 4	3'-5'	no	39	466
106566330	1,835				3'-5'		23	321
107445288	2,29	CUL5	5'-3'	I 7	3'-5'	ok	26	436
110952961	2,575				3'-5'		40	419
111126057	2,745	PPP2R1B	3'-5'	I 10	3'-5'	no	36	393
114035897	2,24				3'-5'		46	426
116463235	2,105	KIAA0999	3'-5'	I 1	3'-5'	no	26	473
121112109	1,92				3'-5'		17	420
122310374	2,455	C11orf63	5'-3'	E 5	3'-5'	ok	28	423
122451569	2,26	ASAM	3'-5'	I 5	3'-5'	no	46	396
123470503	1,64				3'-5'		44	566
123913738	1,625				3'-5'		54	349
124351331	2,05	CCDC15	5'-3'	I 5	3'-5'	ok	47	454
127422345	2,39				3'-5'		37	486
127790377	1,4				3'-5'		50	554

	128616647	2,26				3'-5'		23	406
**Ø**				**38**			**72**	**34,68**	**416,27**
				**35**			**28**	**36,3**	**411,03**
				**73**			**44**	**33,51**	**420,06**
**12**	6181630	1,425	CD9	5'-3'	I 1	5'-3'	no	22	569
	14688045	2,295	GUCY2C	3'-5'	I 16	5'-3'	ok	34	303
153 results	17337414	2,75				5'-3'		37	414
	17979668	2,425				5'-3'		30	449
	21764219	2,48				5'-3'		31	448
	22029995	2,685				5'-3'		36	437
	23635338	2,5	SOX5	3'-5'	I 9	5'-3'	ok	39	444
	23948721	2,485	SOX5	3'-5'	I 1	5'-3'	ok	32	361
	24501483	2,09	SOX5	3'-5'	I 1	5'-3'	ok	50	416
	24798096	2,225				5'-3'		46	429
	25389891	2,705				5'-3'		33	395
	27303878	2,08	STK38L	5'-3'	I 1	5'-3'	no	21	428
	27310675	1,875	STK38L	5'-3'	I 1	5'-3'	no	16	419
	28059586	1,805				5'-3'		20	345
	28497890	2,75	CCDC91	5'-3'	I 10	5'-3'	no	33	414
	29276691	1,81	MLSTD1	5'-3'	I 1	5'-3'	no	42	552
	29901073	1,595				5'-3'		44	243
	31840613	1,9				5'-3'		25	314
	33156131	2,815				5'-3'		36	411
	33613138	1,445				5'-3'		17	303
	38077522	2,495	KIF21A	3'-5'	I 1	5'-3'	ok	38	455
	**44911163**	**2,055**	**SLC38A1**	**3'-5'**	**I 3**	**5'-3'**	ok	**49**	**385**
	**44911163**	**2,055**	**SLC38A1**	**3'-5'**	**I 3**	**5'-3'**	ok	**49**	**385**
	48070786	2,67	SPATS2	5'-3'	I 2	5'-3'	no	37	388
	50386094	1,975	SCN8A	5'-3'	I 10 E 11	5'-3'	no	38	259
	50691984	1,885	GRASP	3'-5'	I 4	5'-3'	ok	22	477
	55747473	2,47	KIAA0286	3'-5'	I 3	5'-3'	ok	31	368
	56477384	2,32	TSFM	5'-3'	E 7	5'-3'	no	40	348
	56827707	2,24				5'-3'		24	426
	60479526	1,815	FAM19A2	3'-5'	I 2	5'-3'	ok	29	561
	60585433	2,245	FAM19A2	3'-5'	I 1	5'-3'	ok	23	415
	60874017	2,795				5'-3'		36	403
	62276035	2,33	DPY19L2	3'-5'	E 15 I 14	5'-3'	ok	45	400
	64476309	1,79				5'-3'		50	342
	65753192	1,815				5'-3'		19	461

65940132	2,39				5'-3'		26	402
66431383	2,235				5'-3'		24	427
69122620	2,61				5'-3'		40	406
74084115	2,705	GLIPR1L2	5'-3'	I 1	5'-3'	no	36	433
74547874	2,245				5'-3'		23	403
75425119	1,625	OSBPL8	3'-5'	I 1	5'-3'	ok	24	269
76127758	2,09				5'-3'		24	456
76487215	2,65				5'-3'		39	414
76823283	2,18	NAV3	5'-3'	I 1	5'-3'	no	24	438
76920706	2,605	NAV3	5'-3'	I 7	5'-3'	no	32	433
**78788370**	**2,64**	**PPP1R12A**	**3'-5'**	**I 2**	**5'-3'**	ok	**36**	**372**
79787764	2,34	LIN7A	3'-5'	I 2	5'-3'	ok	37	496
81330552	2,87	C12orf26	5'-3'	I 5	5'-3'	no	35	410
85307554	2,135	MGAT4C	3'-5'	I 3	5'-3'	ok	24	371
85307791	2,78	MGAT4C	3'-5'	I 3	5'-3'	ok	36	418
85915320	1,965				5'-3'		38	257
87651615	2,25				5'-3'		44	374
88878910	2,7				5'-3'		34	384
89770848	2,34				5'-3'		29	456
90718531	1,72				5'-3'		20	490
91743436	2,3	EEA1	3'-5'	I 13	5'-3'	ok	46	414
92825509	1,825				5'-3'		18	369
93999553	1,765	FGD6	3'-5'	I 20	5'-3'	ok	21	491
94067585	2,54	FGD6	3'-5'	I 5	5'-3'	ok	34	352
95125090	1,95	ELK3	5'-3'	I 1	5'-3'	no	49	364
95386217	2,345				5'-3'		41	455
97111120	2,095				5'-3'		20	403
98668839	1,855	ANKS1B	3'-5'	I 8	5'-3'	ok	16	395
101677886	2,48				5'-3'		29	428
102175224	1,525				5'-3'		23	259
102177489	2,095				5'-3'		27	485
110441907	1,56	ATXN2	3'-5'	I 8 E 8 I 7	5'-3'	ok	51	306
116570807	2,305	KSR2	3'-5'	I 3	5'-3'	ok	27	443
122377090	2,42	SBNO1	3'-5'	I 12	5'-3'	ok	26	408
123055866	2,46	ZNF664	5'-3'	I 4	5'-3'	no	33	472
124631268	2,57	TMEM132B	5'-3'	I 4	5'-3'	no	36	460
128223564	2,465	TMEM132D	3'-5'	I 5	5'-3'	ok	27	407
128633751	1,585	TMEM132D	3'-5'	I 2	5'-3'	ok	24	261
938274	2,64				3'-5'		36	372
1140811	2,675	ERC1	5'-3'	I 7	3'-5'	ok	35	449

1831889	2,665	CACNA2D4	3'-5'	I 23	3'-5'	no	37	387
8115875	2,005				3'-5'		20	433
9965834	1,86				3'-5'		54	422
11039967	2,69	TAS2R49	3'-5'	E 1	3'-5'	no	36	382
13928903	2,72	GRIN2B	3'-5'	I 1	3'-5'	no	38	408
14117205	1,645				3'-5'		46	545
16059572	2,725	DERA	5'-3'	I 6	3'-5'	ok	35	379
16321247	1,965				3'-5'		17	411
17513121	1,825				3'-5'		17	439
17720212	1,53				3'-5'		16	330
20125063	2,715				3'-5'		36	431
21484098	2,28	PYROXD1	5'-3'	I 1 E 2 I 2	3'-5'	ok	45	428
25070177	1,885				3'-5'		54	401
**25138816**	**2,185**	**LRMP**	**5'-3'**	**I 13**	**3'-5'**	ok	**35**	**547**
26206887	1,97				3'-5'		17	408
27034200	1,19	TM7SF3	3'-5'	I 6 E 6	3'-5'	no	53	252
27658421	2,555	PPFIBP1	5'-3'	I 2	3'-5'	ok	39	433
28223185	1,77				3'-5'		22	318
30351948	2,155				3'-5'		22	395
32375546	2,035	BICD1	5'-3'	I 5	3'-5'	ok	34	251
33479244	2,31	SYT10	3'-5'	I 1	3'-5'	no	25	422
37732402	2,67				3'-5'		35	368
38173304	2,145				3'-5'		39	515
43056205	2,755	TMEM117	5'-3'	I 6 E 7 I 7	3'-5'	ok	34	395
43482980	2,03	NELL2	3'-5'	I 4	3'-5'	no	26	330
46063094	1,84				3'-5'		50	352
49542543	2,215	TMPRSS12	5'-3'	I 3	3'-5'	ok	43	357
50952888	2,065				3'-5'		48	441
51264560	1,715				3'-5'		48	511
55270001	1,345				3'-5'		24	605
55933542	2,505	R3HDM2	3'-5'	E 14	3'-5'	no	36	473
56137229	1,915	INHBE	5'-3'	E 2	3'-5'	ok	53	421
58342321	2,135				3'-5'		21	417
58876232	2,2				3'-5'		44	454
59020546	2,6				3'-5'		38	384
62517254	2,6				3'-5'		31	394
63092509	1,6	XPOT	5'-3'	I 2	3'-5'	ok	43	234
65200449	2,83	GRIP1	3'-5'	I 5	3'-5'	no	35	400
66211966	2,19				3'-5'		47	426
68268826	1,27	CCT2	5'-3'	I 6	3'-5'	ok	17	550

72797913	2,265				3'-5'		35	531
73047255	2,65				3'-5'		37	384
73261884	2,135				3'-5'		47	381
76758739	1,88	NAV3	5'-3'	I 1	3'-5'	ok	24	498
77515220	2,745				3'-5'		33	403
79238197	2,02				3'-5'		34	248
79828175	2,23	_IN7A/MALS-1	3'-5'	I 1	3'-5'	no	25	438
79837663	2,395	_IN7A/MALS-1	3'-5'	I 1	3'-5'	no	33	333
81456960	1,765				3'-5'		40	237
82379437	2,46				3'-5'		27	406
85106264	2,06	MGAT4C	3'-5'	I 4	3'-5'	no	49	386
85293297	2,625	MGAT4C	3'-5'	I 3	3'-5'	no	35	459
86925312	2,355	C12orf50	3'-5'	I 3	3'-5'	no	45	413
87841884	2,1				3'-5'		44	344
87940855	2,215				3'-5'		23	397
88121111	1,345				3'-5'		19	555
90713408	2,45				3'-5'		30	444
94096341	2,63	FGD6	3'-5'	I 2	3'-5'	no	37	380
96951434	1,66				3'-5'		46	542
96993007	2,12				3'-5'		46	368
97171455	1,77				3'-5'		24	520
98519284	1,94	ANKS1B	3'-5'	I 9	3'-5'	no	17	416
99309736	1,815	SLC17AB		I 3	3'-5'		19	461
99867629	2,48	TMEM16D	5'-3'	I 4	3'-5'	ok	31	448
101675690	1,59				3'-5'		16	342
103233364	1,935	TXNRD1	5'-3'	I 4 E 5 I 5	3'-5'	ok	38	251
105000862	2,095	NUAK1	3'-5'	I	3'-5'	no	38	535
110485183	2,6	ATXN2	3'-5'	I 1	3'-5'	no	38	434
110499186	1,825	ATXN2	3'-5'	I 1	3'-5'	no	19	459
110869197	2,47	TMEM116	3'-5'	I 6	3'-5'	no	35	490
115403020	1,905				3'-5'		54	405
115753189	2,725	TMEM118	5'-3'	I 8	3'-5'	ok	32	409
117237398	2,12	TAOK3	3'-5'	I 1	3'-5'	no	22	430
117377283	2,145				3'-5'		28	485
118310384	2,555	CCDC60	5'-3'	I 1	3'-5'	ok	40	423
122356033	2,12	SBNO1	3'-5'	I 27	3'-5'	no	27	338
124896431	2,43				3'-5'		37	340
125716791	2,475				3'-5'		31	369
127251702	2,6				3'-5'		40	414
**Ø**			**31**			**78**	**33,16**	**408,16**

				**47**			**36**	**33,56**	**408,64**
				**78**			**42**	**32,75**	**407,67**
**13**	19039961	2,165			I	5'-3'		49	411
	25284336	2,325	ATP8A2	5'-3'	I 20	5'-3'	no	39	339
159 results	28229398	2,505				5'-3'		30	433
	28345620	2,325				5'-3'		24	409
	28550604	2,41	KIAA0774	5'-3'	I 2	5'-3'	no	27	422
	28903091	1,98	KIAA0774	5'-3'	I 6	5'-3'	no	46	478
	31613642	2,095	FRY	5'-3'	I 11	5'-3'	no	28	495
	32558183	1,61				5'-3'		16	346
	32814284	2,005				5'-3'		50	385
	34515995	2,615	NBEA	5'-3'	I 3	5'-3'	no	36	451
	34553166	2,7	NBEA	5'-3'	I 11	5'-3'	no	38	414
	34588008	2,395	NBEA	5'-3'	I 14	5'-3'	no	36	495
	35694758	2,22				5'-3'		45	378
	36519667	2,33	FAM48A	3'-5'	E4 I 4 E 5 I 5	5'-3'	ok	26	428
	37200600	1,65	TRPC4	3'-5'	I 3	5'-3'	ok	16	464
	38182647	2,32	FREM2	5'-3'	I 2	5'-3'	no	46	408
	38302761	2,355	FREM2	5'-3'	I 6	5'-3'	no	26	423
	39012730	1,865	LHFP	3'-5'	I 2	5'-3'	ok	47	491
	39154530	2,37	COG6	5'-3'	I 8	5'-3'	no	45	408
	39313506	2,405				5'-3'		39	463
	40228145	2,11	MRPS31	3'-5'	I 4	5'-3'	ok	49	396
	40392734	2,525				5'-3'		30	389
	41533698	1,73	DGKH	5'-3'	I 1	5'-3'	no	40	588
	43039261	1,385	ENOX1	5'-3'	I 1	5'-3'	no	17	291
	43220254	2,75				5'-3'		35	384
	46445984	2,19				5'-3'		25	446
	46876193	2,445				5'-3'		27	403
	47033105	1,855				5'-3'		16	395
	48643240	2,71	FNDC3A	5'-3'	I 10	5'-3'	no	38	412
	49085581	1,97				5'-3'		18	420
	49953549	2,165				5'-3'		47	387
	50836245	2,18	INTS6	3'-5'	E 19	5'-3'	ok	45	370
	53942382	1,45				5'-3'		21	554
	55135433	2,385				5'-3'		44	417
	55188824	2,235				5'-3'		39	497
	55957275	2,46				5'-3'		30	376
	57170938	2,285	PCDH17	3'-5'	I 3	5'-3'	ok	46	401

57397130	2,28				5'-3'		43	370
58431115	2,43				5'-3'		32	468
60111610	2,375				5'-3'		26	399
60662100	2,56				5'-3'		30	396
63159794	2,44				5'-3'		42	392
63242058	2				5'-3'		31	544
65077293	2,405				5'-3'		40	365
65735124	2,11				5'-3'		40	512
66273453	2,57	PCDH9	5'-3'	I 2	5'-3'	no	32	378
**66410839**	**2,1**	**PCDH9**	5'-3'	**I 2**	**5'-3'**	no	**49**	**394**
70748575	2,685				5'-3'		33	391
70959948	2,355	DACH1	3'-5'	I 3	5'-3'	ok	29	365
72298776	1,995	C13orf24	5'-3'	I 6 E 7 I 7	5'-3'	no	23	353
76742427	1,855	MYCBP2	3'-5'	E 6 I 7	5'-3'	ok	28	543
77302166	2,615				5'-3'		33	441
77806897	2,24				5'-3'		24	426
77859531	1,96				5'-3'		17	406
78323474	2,48				5'-3'		28	400
79700248	2,55				5'-3'		33	454
80673190	2,775				5'-3'		36	399
81122659	1,915				5'-3'		25	317
81494287	2,525				5'-3'		35	479
83283692	2,23				5'-3'		26	370
84332372	2,765				5'-3'		34	397
84464727	2,425				5'-3'		40	449
85748026	2,29				5'-3'		46	402
86855494	2,235				5'-3'		45	437
88688162	2,115				5'-3'		50	407
89519362	2,39				5'-3'		26	402
90231362	2,065				5'-3'		48	377
92517080	2,6				5'-3'		30	404
92553946	2,235				5'-3'		47	401
**93551145**	**1,515**	**GPC6**	**5'-3'**	**I 4**	**5'-3'**	no	**22**	**267**
94294083	2,7				5'-3'		32	414
97069353	1,48				5'-3'		18	300
97153896	2,265				5'-3'		43	451
100384993	1,91				5'-3'		18	432
103844164	2,335				5'-3'		42	371
104423475	1,795				5'-3'		48	495
104520586	1,98				5'-3'		48	360

105404657	2,06				5'-3'		29	512
108819275	1,965				5'-3'		48	357
110601572	1,71	ARHGEF7	5'-3'	I 1	5'-3'	no	18	472
20075069	2,78	IFT88	5'-3'	I 12	3'-5'	ok	36	418
21109718	1,95				3'-5'		23	344
21736650	2,615				3'-5'		33	377
23463881	2,22				3'-5'		39	500
25793043	2,61	CDK8	5'-3'	I 1	3'-5'	ok	38	386
26783094	2,15				3'-5'		36	274
29913984	1,915				3'-5'		21	461
31020273	1,855				3'-5'		47	325
33401355	2,295	RFC3	5'-3'	I 8	3'-5'	ok	44	435
33974732	1,82				3'-5'		16	430
34339196	1,76				3'-5'		48	502
37638777	2				3'-5'		35	234
37665147	1,625				3'-5'		45	559
37923914	2,38				3'-5'		28	380
39938845	2,035				3'-5'		33	557
40631420	2,47				3'-5'		29	430
42036796	1,99				3'-5'		43	312
42542587	2,045	DNAJC15	5'-3'	I 3	3'-5'	ok	40	293
46888451	2,27				3'-5'		43	368
47096899	1,8				3'-5'		28	264
50155143	1,75				3'-5'		19	344
53857709	2,065				3'-5'		51	411
53919194	2,42				3'-5'		27	398
57135153	2,49	PCDH17	5'-3'	I 1	3'-5'	ok	28	416
57709945	1,92				3'-5'		21	358
58107586	2,145				3'-5'		21	403
59042953	1,88				3'-5'		50	458
59177314	2,37	DIAPH3	3'-5'	I 20	3'-5'	no	30	358
59550984	2,105	DIAPH3	3'-5'	I 4	3'-5'	no	45	355
60170899	1,965				3'-5'		27	511
60750801	2,175				3'-5'		24	379
60909717	2,52				3'-5'		32	368
60985532	2,325				3'-5'		25	399
61032387	2,105				3'-5'		25	355
61324505	1,965				3'-5'		17	407
61486048	2,65				3'-5'		39	414
62263810	2,72				3'-5'		32	408

63207261	2,185				3'-5'		22	417
64524342	2,03				3'-5'		19	418
65831448	2,685	PCDH9	3'-5'	I 3	3'-5'	no	38	417
65993148	2,58	PCDH9	3'-5'	I 3	3'-5'	no	40	400
66724167	1,78				3'-5'		49	488
67898604	2,545				3'-5'		30	425
69706396	2,455				3'-5'		30	375
70053666	1,06				3'-5'		18	216
73999222	2,19				3'-5'		48	416
74429330	2,56				3'-5'		35	472
75019844	2,295				3'-5'		31	485
75379447	1,915				3'-5'		28	287
75516500	1,885				3'-5'		23	487
78345799	2,1				3'-5'		45	354
78825784	1,95	RBM26	3'-5'	I 13	3'-5'	no	23	344
80561563	2,45				3'-5'		42	424
81060260	2,645				3'-5'		39	415
81062056	1,995				3'-5'		18	403
81787219	1,53				3'-5'		16	488
83505836	2,57				3'-5'		38	378
84039234	1,765				3'-5'		29	571
84465083	1,945				3'-5'		53	403
86466066	2,27				3'-5'		42	460
86563276	1,89				3'-5'		49	466
88391179	2,18				3'-5'		38	300
88698919	1,91				3'-5'		54	412
89162661	2,64				3'-5'		33	382
89507842	1,96				3'-5'		18	396
90281920	1,78				3'-5'		51	350
93326643	2,345	GPC6	5'-3'	I 3	3'-5'	ok	25	415
93595447	2,315	GPC6	5'-3'	I 4	3'-5'	ok	37	317
93943224	2,18				3'-5'		26	458
95526781	1,865				3'-5'		51	451
96248327	1,95	HS6ST3	5'-3'	I 1	3'-5'	ok	30	544
96471223	2,38				3'-5'		29	370
97296706	1,955				3'-5'		20	375
97891318	2,365	FARP1	5'-3'	I 24	3'-5'	ok	30	357
100881366	2,455				3'-5'		28	395
104881247	1,95				3'-5'		36	234
105016705	1,905				3'-5'		50	365

	106321271	2,025				3'-5'		24	469
	109015371	1,535				3'-5'		43	221
**Ø**				**27**			**40**	**33,89**	**407,21**
				**13**			**23**	**34,4**	**410,03**
				**40**			**17**	**33,72**	**406,27**
**14**	18232873	2,455			I	5'-3'		43	413
	19158149	2,18				5'-3'		41	488
120 results	25496578	2,21				5'-3'		46	432
	25903668	2,445				5'-3'		29	435
	27456008	2,105				5'-3'		47	443
	28330675	1,71				5'-3'		28	246
	29194395	2,22	PRKD1	3'-5'	I 4	5'-3'	ok	42	348
	29435727	2,29	PRKD1	3'-5'	I 1	5'-3'	ok	44	436
	30012325	1,855				5'-3'		22	335
	33849276	2,28				5'-3'		24	418
	36390313	2,8	SLC25A21	3'-5'	I 2	5'-3'	ok	36	414
	39807855	2,045				5'-3'		42	313
	40381046	2,435				5'-3'		34	331
	41365445	2,72	LRFN5	5'-3'	I 2	5'-3'	no	38	408
	41373495	1,58	LRFN5	5'-3'	I 2	5'-3'	no	20	518
	41419386	2,835	LRFN5	5'-3'	I 2	5'-3'	no	35	417
	41694820	2,185				5'-3'		48	417
	41833414	1,68				5'-3'		21	508
	43154297	2,26				5'-3'		45	432
	44006250	2,29				5'-3'		45	392
	45830677	2,37				5'-3'		32	338
	46205912	2,2				5'-3'		47	424
	46270769	1,715				5'-3'		48	511
	48366700	2,16				5'-3'		23	386
	50504509	2,765				5'-3'		35	431
	50714858	1,855				5'-3'		18	443
	53062066	1,78				5'-3'		38	598
	53075840	2,465				5'-3'		39	451
	53870000	1,835				5'-3'		48	487
	56944242	2,08	NAT12	5'-3'	I 4	5'-3'	no	45	350
	59048226	2,45				5'-3'		29	434
	59175730	2,68	RTN1	3'-5'	I 3	5'-3'	ok	32	400
	61301273	2,385	SNAPC1	5'-3'	I 1	5'-3'	no	27	391
	61655352	2,07				5'-3'		28	318

61692714	2,655				5'-3'		35	365
62863773	1,805				5'-3'		29	255
**65277499**	**1,625**	**FUT8**	**5'-3'**	**I 10**	**5'-3'**	no	**51**	**319**
65381909	2,49				5'-3'		28	416
72610867	2,39	RBM25	5'-3'	I 2	5'-3'	no	30	456
77580155	2,39				5'-3'		27	426
78510092	2,645	NRXN3	5'-3'	I 11	5'-3'	no	35	363
78660780	2,52	NRXN3	5'-3'	I 12	5'-3'	no	36	470
80695792	2,745				5'-3'		36	425
81040111	2,395	SEL1L	3'-5'	I 5 E 5 I 4	5'-3'	ok	39	353
84527198	1,775				5'-3'		21	489
84528599	2,2				5'-3'		24	384
84543717	2,395				5'-3'		27	393
88188513	1,845	EML5	3'-5'	I 28	5'-3'	ok	18	373
90371720	1,885				5'-3'		42	281
91064900	1,925				5'-3'		20	449
91127658	2,785	C14orf161	3'-5'	I 23 E 23 I 22	5'-3'	ok	35	427
92354428	2,515	GOLGA5	5'-3'	I 7	5'-3'	no	30	431
94631170	2,35	DICER1	3'-5'	I 24 E 24	5'-3'	ok	33	324
97001526	1,48				5'-3'		51	290
98179476	1,81				5'-3'		19	462
99789702	2,425	YY1	5'-3'	I 1	5'-3'	no	42	389
100840419	1,235				5'-3'		17	261
105613793	2,275				5'-3'		29	469
18554672	2,18				3'-5'		41	488
20802630	2,685	HNRNPC	3'-5'	E 2 I 1	3'-5'	no	34	381
21080871	2,035				3'-5'		51	401
21246766	2,03				3'-5'		44	330
26349235	2,715				3'-5'		37	421
26905841	2,12				3'-5'		20	408
29359474	2,52	PRKD1	3'-5'	I 1	3'-5'	no	33	358
29371485	2,425	PRKD1	3'-5'	I 1	3'-5'	no	30	449
31344604	1,93	NUBPL	5'-3'	I 7	3'-5'	ok	51	380
31638298	1,99	ARHGAP5	5'-3'	I 2	3'-5'	ok	20	382
32552182	1,95	NPAS3	5'-3'	I 1	3'-5'	ok	53	414
32832098	2,615	NPAS3	5'-3'	I 2	3'-5'	ok	33	441
32853987	2,345	NPAS3	5'-3'	I 2	3'-5'	ok	27	383
32878973	2,195	NPAS3	5'-3'	I 2	3'-5'	ok	37	293
33739394	1,975				3'-5'		31	269
34813576	2,625				3'-5'		31	419

37423578	2,6				3'-5'		37	444
37428335	1,835				3'-5'		54	391
37428492	1,315				3'-5'		53	541
37578114	2,725				3'-5'		35	439
38259192	2,025				3'-5'		37	259
38995964	2,105				3'-5'		35	563
41627270	2,55				3'-5'		29	414
41952777	2,465				3'-5'		28	421
42683509	2,705				3'-5'		38	413
44072123	2,265				3'-5'		44	377
46100581	2,775				3'-5'		33	409
46425657	2,34	MDGA2	3'-5'	I 10	3'-5'	no	42	372
46736304	1,71	MDGA2	3'-5'	I 3	3'-5'	no	18	346
46882180	2,355	MDGA2	3'-5'	I 1	3'-5'	no	40	463
47377174	2,15				3'-5'		49	404
47672011	2,865				3'-5'		35	407
47882013	1,13				3'-5'		18	230
47997247	1,525				3'-5'		18	509
48434971	1,705				3'-5'		54	453
50317611	2,625	NIN	3'-5'	I 4	3'-5'	no	32	389
51259740	2,125	FRMD6	5'-3'	I 12	3'-5'	ok	50	409
52335179	1,93				3'-5'		18	390
58190302	1,805				3'-5'		19	355
59729622	1,815				3'-5'		27	277
60026847	1,935				3'-5'		49	361
60583464	2,495	SLC38A6	5'-3'	I 12	3'-5'	ok	30	435
61031710	2,28	PRKCH	5'-3'	I 10	3'-5'	ok	43	370
61571687	2,385	SYT16	5'-3'	I 1	3'-5'	ok	43	391
61733164	1,575				3'-5'		20	299
63233597	2,215	SGPP1	3'-5'	I 2	3'-5'	no	26	367
63885512	1,975				3'-5'		18	419
65934866	2,065				3'-5'		51	411
66450430	2,21	GPHN	5'-3'	I 5	3'-5'	ok	47	422
70505100	2,27	PCNX	5'-3'	I 4 E 5 I 5	3'-5'	ok	40	338
72231270	1,905	DPF3	3'-5'	I 5	3'-5'	no	27	295
72858484	2,785	NUMB	3'-5'	I 5	3'-5'	no	34	401
72963644	1,615	NUMB	3'-5'	I 1	3'-5'	no	17	481
75694075	2,44	C14orf118	5'-3'	I 2	3'-5'	ok	39	362
78906788	1,925	NRXN3	5'-3'	I 12	3'-5'	ok	19	379
83685331	2,455				3'-5'		31	365

	86626014	2,23				3'-5'		47	418
	86789902	2,05				3'-5'		23	454
	88413017	2,485	TTC8	5'-3'	I 14 E 15	3'-5'	ok	40	437
	88678211	2,1				3'-5'		50	404
	91728827	1,875				3'-5'		26	299
	103166165	2,32	KLC1	5'-3'	I 1	3'-5'	ok	44	430
**Ø**				**27**			**46**	**34,61**	**398,38**
				**19**			**22**	**35,15**	**394,67**
				**46**			**24**	**34,27**	**400,69**
**15**	18596258	2,47			I	5'-3'		32	358
	19607508	2,47				5'-3'		32	358
85 results	19849668	2,53				5'-3'		36	350
	22519789	1,905				5'-3'		50	365
	27308110	2,47				5'-3'		39	368
	35801362	2,395				5'-3'		27	425
	36131728	2,15				5'-3'		22	424
	37680074	1,575	FSIP1	3'-5'	E 12 I 11	5'-3'	ok	25	569
	39849175	2,43	MGA	5'-3'	E 24	5'-3'	no	43	418
	40996629	2,56	TTBK2	3'-5'	I 1	5'-3'	ok	39	386
	42370017	2,605	CASC4	5'-3'	I 1	5'-3'	no	38	433
	44173445	2,385				5'-3'		44	401
	44359415	2,735				5'-3'		35	437
	46158418	1,955				5'-3'		27	513
	**46965860**	**2,805**	**SHC4**	**3'-5'**	**I 3**	**5'-3'**	ok	**35**	**395**
	47906699	2,315				5'-3'		41	357
	49157501	1,48	TNFAIP8L3	3'-5'	I 2	5'-3'	ok	47	250
	50596785	2,185	MYO5A	3'-5'	I 1	5'-3'	ok	47	427
	50708044	2,48	KIAA1370	3'-5'	I 2	5'-3'	ok	34	340
	52789173	1,72				5'-3'		23	298
	55018977	2,27	TCF12	5'-3'	I 3	5'-3'	no	41	470
	57528093	2,37	FAM81A	5'-3'	I 1	5'-3'	no	29	368
	58182986	2,815				5'-3'		35	421
	58770084	2,27	RORA	3'-5'	I 1	5'-3'	ok	29	470
	59913083	1,825				5'-3'		28	549
	69071146	2,7	LRRC49	5'-3'	I 11	5'-3'	no	34	384
	70423230	2,15	HEXA	3'-5'	I 13 E 13 I 12	5'-3'	ok	44	354
	71128564	1,61				5'-3'		19	316
	72646277	2,54	ARID3B	5'-3'	I 2	5'-3'	no	40	426
	75280338	2,36				5'-3'		41	452

77756432	2,39				5'-3'		26	402
79697489	2,31				5'-3'		42	452
86419767	1,77	NTRK3	3'-5'	I 12	5'-3'	ok	17	450
87938028	2,16	C15orf42	5'-3'	I 6	5'-3'	no	22	396
94412795	2,485				5'-3'		39	371
96838247	2,525	FLJ39743	3'-5'	I 4	5'-3'	ok	37	359
96840922	2,47	FLJ39743	3'-5'	I 4 E 4 I 3	5'-3'	ok	37	470
99270977	2,735	ALDH1A3	5'-3'	I 12 E 12	5'-3'	no	33	417
99511066	2,035				5'-3'		25	341
18787184	2,48				3'-5'		32	360
19129288	2,63				3'-5'		36	370
19182082	2,28				3'-5'		26	380
20482431	2,655	CYFIP1	5'-3'	I 6	3'-5'	ok	37	385
23794172	2,04				3'-5'		51	402
24482273	2,21	GABRB3	3'-5'	I 3	3'-5'	no	25	376
24741296	2,115	LOC441742	5'-3'	I 1	3'-5'	ok	20	411
25475540	2,32				3'-5'		25	420
27100006	2,6	APBA2	5'-3'	I 2	3'-5'	ok	35	464
27547812	2,38				3'-5'		31	468
30582986	2,62				3'-5'		39	420
32116823	1,975	AVEN/CHRM5	3'-5'	I 1	3'-5'	no	22	359
33747295	1,65				3'-5'		44	564
33865445	2,09				3'-5'		47	372
36483537	1,45				3'-5'		44	214
38579614	1,9				3'-5'		16	414
42727584	2,635	SPG11	3'-5'	I 7	3'-5'	no	36	371
49444318	2,545	GLDN	5'-3'	I 1	3'-5'	ok	31	383
52390913	2,035	UNC13C	5'-3'	I 12	3'-5'	ok	19	401
54471604	1,985	TEX9	5'-3'	I 7	3'-5'	ok	31	271
62643104	1,57	ZNF609	5'-3'	I 1	3'-5'	ok	45	248
63626781	1,89	PTPLAD1	5'-3'	I 1	3'-5'	ok	43	526
64387545	1,85	DIS3L	5'-3'	I 3 E 4	3'-5'	ok	49	344
64400157	2,435	DIS3L	5'-3'	I 9	3'-5'	ok	27	401
65082876	2,61				3'-5'		32	386
65315777	2,265	FLJ11506	3'-5'	I 1 E 2 I 2 E 3 I 3	3'-5'	no	24	421
65686715	1,57	MAP2K5	5'-3'	I 8	3'-5'	ok	42	218
65918879	2,315				3'-5'		28	367
68794020	2,185	UACA	3'-5'	I 1	3'-5'	no	22	401
69925360	1,505	MYO9A	3'-5'	I 39	3'-5'	no	19	523
70592139	1,57	ARIH1	5'-3'	I 1	3'-5'	ok	20	298

	71037272	2,075				3'-5'		49	389
	71575868	2,78	LOC283677	5'-3'	I 2	3'-5'	ok	34	418
	74944398	2,24	SCAPER	3'-5'	I 1	3'-5'	no	47	402
	77980392	2,455	ST20	3'-5'	I 2	3'-5'	no	28	395
	78042804	2,385	BCL2A1	3'-5'	I 1	3'-5'	no	28	381
	**78520611**	**1,865**	**ARNT2**	**5'-3'**	**I 1**	**3'-5'**	ok	**19**	**451**
	80194641	1,97				3'-5'		50	440
	81663439	2,635	HDGFRP3	3'-5'	I 1	3'-5'	no	37	381
	82507668	2,025				3'-5'		36	569
	84380993	2,525				3'-5'		39	439
	84924276	1,93	LOC731136	5'-3'	I 2	3'-5'	ok	17	400
	90842544	1,975	C15orf32	5'-3'	I 2	3'-5'	ok	43	509
	96012906	1,95				3'-5'		19	384
	96069712	1,96				3'-5'		22	356
	98628293	1,96	ADAMTS17	3'-5'	I 4	3'-5'	no	53	412
**Ø**				**24**			**46**	**33,56**	**400,05**
				**22**			**19**	**33,13**	**398,52**
				**46**			**27**	**34,08**	**401,85**
**16**	46	1,2			I	5'-3'		20	224
	2207814	2,305				5'-3'		32	325
81 results	14182587	2,455	MKL2	5'-3'	I 3	5'-3'	no	43	413
	15842433	2,33	MYH11	3'-5'	I 1	5'-3'	ok	25	418
	17487577	2,31				5'-3'		41	462
	17670683	2,48				5'-3'		28	400
	18808932	1,77	SMG1	3'-5'	I 5	5'-3'	ok	43	550
	21396178	1,865				5'-3'		43	531
	21827971	1,865				5'-3'		43	531
	23522270	2,03	PALB2	3'-5'	E 13 I 12	5'-3'	ok	21	380
	24552140	2,55				5'-3'		39	384
	29478244	1,865				5'-3'		43	531
	30217956	1,865				5'-3'		43	531
	31209272	2,555	ITGAM	5'-3'	I 12	5'-3'	no	41	405
	32244905	2,48				5'-3'		32	360
	47177883	2,205	N4BP1	3'-5'	I 1	5'-3'	ok	23	395
	49352156	2,36	CYLD	5'-3'	I 6	5'-3'	no	43	386
	49357347	2,59	CYLD	5'-3'	I 6	5'-3'	no	32	382
	51063949	2,285	TOX3	3'-5'	I 1	5'-3'	ok	26	437
	51073724	2,365	TOX3	3'-5'	I 1	5'-3'	ok	31	471
	51157879	2,75				5'-3'		33	414

51580600	1,74				5'-3'		26	272
52776826	2,63				5'-3'		32	390
53446729	2				5'-3'		33	254
55001061	1,575	AMFR	3'-5'	I 2	5'-3'	ok	22	539
59426988	2,255				5'-3'		25	385
60093731	2,7				5'-3'		38	404
60580847	1,965	CDH8	3'-5'	I 2	5'-3'	ok	51	431
61863100	2,175				5'-3'		22	399
63303938	2,335				5'-3'		28	371
63355574	2,355				5'-3'		41	453
68031878	2,535	CYB5B	5'-3'	I 1	5'-3'	no	34	351
68341024	1,99	NOB1	3'-5'	E 4 I 3	5'-3'	ok	20	382
71067381	2,545				5'-3'		41	415
71770132	2,06				5'-3'		46	462
73714066	2,49				5'-3'		31	372
78505354	2,705				5'-3'		38	405
82956836	1,585				5'-3'		51	507
1597620	1,51				3'-5'		22	266
6390811	2,38	A2BP1	5'-3'	I 2	3'-5'	ok	26	418
6544716	1,285	A2BP1	5'-3'	I 2	3'-5'	ok	18	261
6781572	1,94	A2BP1	5'-3'	I 3	3'-5'	ok	17	416
7818197	2,375				3'-5'		25	409
10154295	2,115	GRIN2A	3'-5'	I 3	3'-5'	no	37	277
11732285	2,415	TXNDC11	3'-5'	I 3	3'-5'	no	29	441
12303548	2,545	LOC92017	5'-3'	I 9	3'-5'	ok	30	393
13043691	1,92				3'-5'		27	298
20029764	2,21				3'-5'		48	412
20941623	1,885	DNAH3	3'-5'	I 39	3'-5'	no	34	221
21041882	2,495	DNAH3	3'-5'	I 9	3'-5'	no	29	425
22379775	1,865				3'-5'		43	531
22694296	1,91				3'-5'		53	422
24843282	2,325	ARHGAP17	3'-5'	I 19	3'-5'	no	28	369
25534763	2,1				3'-5'		44	474
26315029	2,37				3'-5'		32	338
26405549	1,675				3'-5'		54	459
33848072	2,065				3'-5'		51	411
45153530	1,905				3'-5'		53	395
52236327	2,755	RPGRIP1L	3'-5'	I 17	3'-5'	no	35	385
54218748	2,245				3'-5'		41	475
54544030	2,27				3'-5'		25	388

	58507014	1,86				3'-5'		20	356
	60469226	2,165	CDH8	3'-5'	I 3	3'-5'	no	27	471
	62678487	2,155				3'-5'		38	523
	62835735	1,61				3'-5'		27	236
	63460658	2,195				3'-5'		46	435
	63525506	1,925				3'-5'		48	469
	67252190	2,075	CDH3	5'-3'	I 2	3'-5'	ok	19	409
	70823851	1,635				3'-5'		23	281
	72611149	2,31				3'-5'		32	326
	73735321	2,215				3'-5'		23	397
	74720038	2,29				3'-5'		46	402
	76422395	2,46	KIAA1576	5'-3'	I 3	3'-5'	ok	28	396
	76431772	2,4	KIAA1576	5'-3'	I 3	3'-5'	ok	28	434
	77620392	1,88	WWOX	5'-3'	I 5	3'-5'	ok	41	270
	77622920	2,455	WWOX	5'-3'	I 5	3'-5'	ok	36	483
	77624004	2,615	WWOX	5'-3'	I 5	3'-5'	ok	36	451
	79605929	1,66	CENPN	5'-3'	I 3	3'-5'	ok	21	306
	80752592	2,25	MPHOSPH6	3'-5'	I 2	3'-5'	no	45	434
	81423564	2,465	CDH13	5'-3'	I 1	3'-5'	ok	27	407
	82094601	2,195	CDH13	5'-3'	I 1	3'-5'	ok	42	475
**Ø**				**18**			**35**	**34,05**	**402,06**
				**17**			**13**	**31,09**	**402,34**
				**35**			**22**	**36,3**	**401,85**
**17**	1064471	2,675			I	5'-3'		38	399
	1737617	1,565	RPA1	5'-3'	I 13	5'-3'	no	45	571
68 results	1746481	1,23	RPA1	5'-3'	I 16	5'-3'	no	20	230
	7999126	2,22				5'-3'		22	408
	12654959	1,605	RICH2	5'-3'	I 1	5'-3'	no	41	603
	13219652	2,755				5'-3'		37	405
	14042952	1,745	COX10	5'-3'	I 6	5'-3'	no	24	293
	16513888	2,575				5'-3'		30	419
	19236523	2,42				5'-3'		27	420
	21424902	2,535				5'-3'		33	361
	26573299	1,89	NF1	5'-3'	I 15	5'-3'	no	18	436
	27266679	2,125				5'-3'		50	409
	28147378	1,7	MYO1D	3'-5'	I 1	5'-3'	ok	17	464
	28164039	1,865	MYO1D	3'-5'	I 1	5'-3'	ok	45	307
	30937505	1,675				5'-3'		19	329
	33684122	1,82				5'-3'		21	480

33932347	2,615				5'-3'		40	407
42597359	2,755	CDC27	3'-5'	I 4	5'-3'	ok	34	395
42604094	1,82	CDC27	3'-5'	I 3 E 3 I 2	5'-3'	ok	16	430
43717037	2,39	SKAP1	3'-5'	I 4	5'-3'	ok	27	392
44858177	2,57				5'-3'		31	430
46471465	2,35	SPAG9	3'-5'	I 5	5'-3'	ok	42	444
48561290	2,115				5'-3'		23	377
49398485	2,075				5'-3'		23	449
51086450	2,53				5'-3'		29	400
52295041	2,215	DGKE	5'-3'	E 11	5'-3'	no	23	421
53962059	2,455	SEPT4	3'-5'	I 1	5'-3'	ok	31	453
56562306	2,385	BCAS3	5'-3'	I 22	5'-3'	no	43	427
56987224	2,075				5'-3'		51	409
57032603	2,275				5'-3'		40	479
57929291	2,095	TLK2	5'-3'	I 2	5'-3'	no	48	383
58591560	2,61				5'-3'		39	396
59259967	2,3	PSMC5	5'-3'	I 2 E 3	5'-3'	no	29	354
63284722	1,86	BPTF	5'-3'	I 2	5'-3'	no	41	266
63722879	1,86				5'-3'		41	266
65586106	2,385	KCNJ16	5'-3'	I 1	5'-3'	no	42	437
66082405	2,345				5'-3'		25	403
68567518	2,175	SLC39A11	3'-5'	I 3	5'-3'	ok	40	499
78176122	1,475	WDR45L	3'-5'	I 5 E 5 I 4	5'-3'	ok	23	569
6576465	2,625				3'-5'		40	409
8787363	1,745				3'-5'		53	455
9168866	1,815	STX8	3'-5'	I 7	3'-5'	no	40	247
11746857	1,115	DNAH9	5'-3'	E 60 I 60	3'-5'	ok	16	247
12361171	2,63				3'-5'		33	380
13958175	2,27	COX10	5'-3'	I 4	3'-5'	ok	47	408
14567823	2,315				3'-5'		40	347
17849998	1,735	LRRC48	5'-3'	I 10 E 11 I 11	3'-5'	ok	20	331
21742260	2,535				3'-5'		33	361
22306637	2,03				3'-5'		51	418
26555677	2,325	NF1	5'-3'	I 11	3'-5'	ok	41	459
31264094	1,72				3'-5'		48	510
31293041	2,585				3'-5'		30	417
36271157	2,7	KRT12	3'-5'	E 8 I 7	3'-5'	no	34	434
36754503	2,425				3'-5'		26	409
46464823	2	SPAG9	3'-5'	I 5	3'-5'	no	20	434
**47228679**	**2,72**	**CA10**	**3'-5'**	**I 3**	**3'-5'**	no	**37**	**420**

	48678979	1,805				3'-5'		17	375
	49413982	1,415				3'-5'		45	217
	50641710	2,575				3'-5'		30	419
	52619436	2,27				3'-5'		47	408
	53894347	1,67	HSF5	3'-5'	I 4	3'-5'	no	18	480
	56885404	2,22				3'-5'		48	410
	57441315	2,535	MED13	3'-5'	I 9	3'-5'	no	40	391
	60671356	2,255				3'-5'		43	453
	64375581	2,875	ABCA8	3'-5'	E 37 I 37 E 36 I 3	3'-5'	no	35	409
	66276111	1,75				3'-5'		22	314
	69433603	1,885				3'-5'		46	497
	72246502	1,8	MFSD11	5'-3'	I 2 E 3 I 3	3'-5'	ok	26	534
**Ø**				**16**			**32**	**33,74**	**406,07**
				**16**			**18**	**31,97**	**411,5**
				**32**			**14**	**35,31**	**401,25**
**18**	1180474	2,095			I	5'-3'		21	425
	3091644	2,275	MYOM1	3'-5'	I 23	5'-3'	ok	28	359
116 results	3556728	2,51	DLGAP1	3'-5'	I 7	5'-3'	ok	33	462
	3925173	1,705				5'-3'		22	305
	4152467	2,535				5'-3'		40	391
	4433861	2,765				5'-3'		34	421
	9271859	2,715	ANKRD12	5'-3'	E 13	5'-3'	no	35	377
	11156985	1,47				5'-3'		19	530
	11548809	2,56				5'-3'		35	472
	17361489	1,845				5'-3'		29	555
	19078544	2,1	CABLES1	5'-3'	I 7	5'-3'	no	48	384
	20397687	2,555				5'-3'		34	463
	22921505	1,86	CHST9	3'-5'	I 2	5'-3'	ok	51	452
	25521913	1,995				5'-3'		51	425
	26481309	2,465				5'-3'		27	407
	27128754	2,34				5'-3'		37	322
	34323893	2,15				5'-3'		46	444
	35299044	2,14				5'-3'		25	456
	35437710	1,92				5'-3'		30	268
	35475006	2,725				5'-3'		33	399
	36002443	2,5				5'-3'		42	414
	37233952	2,46				5'-3'		27	406
	37329505	1,365				5'-3'		17	531
	38684869	2,28	RIT2	3'-5'	I 4	5'-3'	ok	24	418

38933395	2,425	RIT2	3'-5'	I 1	5'-3'	ok	43	399
39013709	1,855				5'-3'		17	385
39620366	1,95				5'-3'		47	474
**41229807**	**1,675**				**5'-3'**		**21**	**509**
42859600	2,205	KATNAL2	5'-3'	I 12	5'-3'	no	22	413
43224742	2,07				5'-3'		48	440
43547936	2,13				5'-3'		23	380
45819369	2,35	MYO5B	3'-5'	I 3	5'-3'	ok	38	334
45845095	1,895	MYO5B	3'-5'	I 1	5'-3'	ok	22	343
46972030	2,5	MEX3C	3'-5'	I 1	5'-3'	ok	31	444
48492821	1,265	DCC	5'-3'	I 1	5'-3'	no	16	541
50212386	2,12				5'-3'		23	440
56805776	2,55				5'-3'		33	454
57555564	2,195				5'-3'		41	333
58768496	2,435	PHLPP	5'-3'	I 13	5'-3'	no	33	477
61840830	2,365				5'-3'		26	421
62248737	2,39				5'-3'		43	392
63103099	2,265				5'-3'		47	411
64415202	2,275				5'-3'		43	369
64855387	2,115	CCDC102B	5'-3'	I 6	5'-3'	no	21	397
65813084	2,735				5'-3'		33	401
68528257	2,285				5'-3'		33	311
68622288	1,91	NETO1	3'-5'	I 4	5'-3'	ok	18	386
68631083	1,955	NETO1	3'-5'	I 4	5'-3'	ok	26	503
68850519	2,705				5'-3'		34	433
69390852	2,27				5'-3'		24	420
70684326	2,225	ZNF407	5'-3'	I 3	5'-3'	no	28	469
70991347	2,07				5'-3'		19	410
73322082	1,98				5'-3'		23	468
74206796	2,005				5'-3'		20	385
1443280	2,71				3'-5'		37	396
1717706	2,55				3'-5'		39	434
1894295	2,305				3'-5'		44	385
3243479	1,77	MRCL3	5'-3'	I 2 E 3 I 3	3'-5'	ok	17	450
5074608	2,44				3'-5'		43	416
7028342	1,6	LAMA1	3'-5'	I 11 E 11 I 10	3'-5'	no	51	504
7905901	1,745	PTPRM	5'-3'	I 4	3'-5'	ok	54	445
11097455	2,005				3'-5'		19	395
13811148	2,12				3'-5'		37	278
14069023	2,18				3'-5'		24	380

17770927	2,025				3'-5'		52	409
18402623	2,565				3'-5'		30	421
22997738	2,465	CHST9	3'-5'	I 1	3'-5'	no	32	357
**23292389**	**2,16**				**3'-5'**		**26**	**462**
23707691	2,135				3'-5'		49	417
23896462	2,32	CDH2	3'-5'	I 2	3'-5'	no	29	460
25918778	2,105				3'-5'		48	433
27879196	2,585	RNF125	5'-3'	I 3 E 4 I 4	3'-5'	ok	33	447
28797348	1,98	C18orf34	3'-5'	I 21	3'-5'	no	49	448
28814902	1,885	C18orf34	3'-5'	I 20	3'-5'	no	51	371
29883901	1,595	NOL4	3'-5'	I 5	3'-5'	no	54	475
31205752	2,21	ZNF396	3'-5'	I 3	3'-5'	no	27	462
31758435	2,445				3'-5'		33	343
34374270	2,385				3'-5'		39	351
34467977	2,545				3'-5'		30	393
34872934	2,4				3'-5'		42	384
35842704	2,365				3'-5'		42	377
37352141	2,47				3'-5'		31	450
37622743	1,91				3'-5'		51	376
38756074	1,845	RIT2	3'-5'	I 4	3'-5'	no	17	383
41235289	2,11				3'-5'		47	376
42385525	2,545				3'-5'		32	445
43264205	2,325				3'-5'		30	469
43829423	1,985				3'-5'		38	557
48342075	1,355	DCC	5'-3'	I 1	3'-5'	ok	54	523
48747980	1,935	DCC	5'-3'	I 5	3'-5'	ok	17	417
48782194	2,58	DCC	5'-3'	I 5	3'-5'	ok	39	428
49373938	1,745				3'-5'		28	565
49500554	2,56				3'-5'		38	376
50148387	2,39	C18orf54	5'-3'	I 5	3'-5'	ok	37	486
50520556	2,12				3'-5'		28	328
**51054845**	**1,72**	**TCF4**	**3'-5'**	**I 15**	**3'-5'**	no	**53**	**358**
52592721	1,91	WDR7	5'-3'	I 16	3'-5'	ok	33	236
56770460	2,075				3'-5'		50	399
58317515	1,565				3'-5'		51	511
58548951	2,105	PHLPP	3'-5'	I 2	3'-5'	no	37	543
58948215	1,86	BCL2	3'-5'	I 2	3'-5'	no	52	376
59409383	2,005	SERPINB13	5'-3'	I 3	3'-5'	ok	29	295
60596819	2,53				3'-5'		32	370
61831334	1,675				3'-5'		16	359

	62100901	2,37				3'-5'		43	430
	62330350	2,19	CDH19	3'-5'	I 9	3'-5'	no	25	372
	63319502	2,46				3'-5'		27	412
	65831373	1,98	RTTN	3'-5'	I 46	3'-5'	no	40	280
	65982816	1,85	RTTN	3'-5'	I 14	3'-5'	no	16	394
	66549290	2,38				3'-5'		40	360
	67334483	2,45				3'-5'		42	424
	67382266	2,36	[illegible]48957/AK093	3'-5'	I 1	3'-5'	no	38	482
	68735849	2,55				3'-5'		35	344
	68795614	2				3'-5'		40	534
	70716323	2,04	ZNF407	5'-3'	I 3	3'-5'	ok	42	506
	71263201	1,865	LOC284274	3'-5'	I 1	3'-5'	no	17	387
**Ø**				**17**			**43**	**34,14**	**416,14**
				**26**			**23**	**33,95**	**419,6**
				**43**			**20**	**34,25**	**414,1**
**19**	**6935702**	**1,695**	**EMR4**	**3'-5'**	**E 5 I 4**	**5'-3'**	ok	**48**	**303**
	14805360	2,49				5'-3'		34	342
24 results	20075971	1,84				5'-3'		48	332
	21903769	2,26				5'-3'		35	532
	22899464	2,155				5'-3'		35	265
	35197605	2,8	C19orf2	5'-3'	I 10 E 11	5'-3'	no	35	394
	35997270	1,435				5'-3'		48	567
	36640476	2,395				5'-3'		29	373
	37396007	2,795				5'-3'		34	415
	39167479	1,04				5'-3'		16	586
	42662880	2,35	ZNF570	5'-3'	I 4	5'-3'	no	25	414
	55232947	1,97	ZNF473	5'-3'	I 2	5'-3'	no	29	288
	6905898	1,78				3'-5'		45	290
	12448350	2,45	ZNF709	3'-5'	I 1	3'-5'	no	31	454
	20063051	2,135				3'-5'		40	311
	22074797	2,16				3'-5'		46	442
	33138981	2,67				3'-5'		37	388
	33912708	2,095				3'-5'		20	403
	37056396	2,405				3'-5'		27	395
	40782685	1,945				3'-5'		18	425
	46741488	2,055				3'-5'		50	395
	49572256	1,86				3'-5'		18	442
	51350386	1,995	IGFL2	5'-3'	I 2	3'-5'	ok	22	363
	61764611	2,025				3'-5'		25	479

**Ø**				**4**			**6**	**33,13**	**399,92**
				**2**			**4**	**31,67**	**369,33**
				**6**			**2**	**33,61**	**410,11**
**20**	88649	2,795			I	5'-3'		34	403
	4441354	2,44				5'-3'		39	362
61 results	4927768	2,565	SLC23A2	3'-5'	I 1	5'-3'	ok	40	397
	10156213	2,275	SNAP25	5'-3'	I 1	5'-3'	no	24	419
	14311468	2,655	MACROD2	5'-3'	I 3	5'-3'	no	32	395
	**15432054**	**2,09**	**MACROD2**	5'-3'	**I 8**	**5'-3'**	no	**49**	**426**
	16564689	2,12				5'-3'		20	408
	21274366	2,865	XRN2	5'-3'	I 16	5'-3'	no	35	407
	25080266	1,975				5'-3'		49	449
	31655305	1,725	CBFA2T2	5'-3'	I 1	5'-3'	no	48	509
	32788395	1,92	NCOA6	3'-5'	I 12	5'-3'	ok	29	278
	33087066	2,695	TRPC4AP	3'-5'	I 7	5'-3'	ok	33	425
	33714430	2,625	CPNE1/RBM1[illegible]	3'-5'	I 1/I 1	5'-3'	ok	38	389
	36618656	1,885	KIAA1219	5'-3'	I 22	5'-3'	no	50	361
	37225861	1,855				5'-3'		26	295
	37629536	2,21				5'-3'		25	376
	37828758	2,225				5'-3'		23	419
	38149987	2,76				5'-3'		33	412
	40395332	2,685	PTPRT	3'-5'	I 11	5'-3'	ok	32	401
	40877846	2,07	PTPRT	3'-5'	I 2	5'-3'	ok	36	560
	**49702519**	**1,875**	**ATP9A**	**3'-5'**	**I 14**	**5'-3'**	ok	**34**	**599**
	53194950	2,085				5'-3'		25	351
	54313955	1,915				5'-3'		18	387
	55354631	2,105				5'-3'		47	443
	59455225	2,76	CDH4	5'-3'	I 2	5'-3'	no	35	386
	3056657	1,645	UBOX5	3'-5'	I 1	3'-5'	no	18	333
	5952345	1,865	CRLS1	5'-3'	I 3	3'-5'	ok	41	551
	6199943	1,86				3'-5'		28	542
	7338999	2,32				3'-5'		25	420
	7360163	2,75				3'-5'		33	404
	8497721	2,35	PLCB1	5'-3'	I 3	3'-5'	ok	45	414
	8551552	2,345	PLCB1	5'-3'	I 3	3'-5'	ok	42	373
	9938478	2,425				3'-5'		30	369
	11253886	1,78				3'-5'		51	350
	11530514	2,365				3'-5'		38	337
	12020524	2,36				3'-5'		27	386

	12183596	2,675				3'-5'		33	389
	13452242	1,98	TASP1	3'-5'	I 10	3'-5'	no	21	448
	16621073	1,81				3'-5'		30	572
	19418653	1,715	SLC24A3	5'-3'	I 2	3'-5'	ok	52	347
	20912270	1,955				3'-5'		46	335
	29830068	1,8	TPX2	5'-3'	I 9 E 10 I 10	3'-5'	ok	50	344
	30854124	2,485	DNMT3B	5'-3'	I 20	3'-5'	ok	42	417
	31636928	1,81	CBFA2T2	5'-3'	I 1	3'-5'	ok	30	246
	33679879	2,56	CPNE1	3'-5'	I 13	3'-5'	no	41	406
	36161961	2,645				3'-5'		38	425
	37167993	2,03				3'-5'		28	508
	39970882	2,05				3'-5'		22	374
	42265519	2,585	C20orf111	3'-5'	I 2	3'-5'	no	36	457
	42279313	1,35				3'-5'		18	274
	42552121	2,44	C20orf121	5'-3'	E 5	3'-5'	ok	41	382
	43069118	2,08	STK4	5'-3'	I 9	3'-5'	ok	38	280
	46992758	2,425	ARFGEF2	5'-3'	I 3	3'-5'	ok	37	339
	47929226	1,92	SLC9A8	5'-3'	I 12	3'-5'	ok	16	408
	49066004	2,605	KCNG1	3'-5'	I 1	3'-5'	no	31	395
	50521157	2,41				3'-5'		32	472
	50830801	2,055				3'-5'		31	285
	51346036	2,07	TSHZ2	5'-3'	I 2	3'-5'	ok	19	408
	55343801	2,34	SPO11	5'-3'	I 6 E 7 I 7 E 8 I 8	3'-5'	ok	39	476
	56239556	2,07				3'-5'		21	388
	57271166	2,335				3'-5'		40	351
**Ø**				**20**			**32**	**33,84**	**401,02**
				**12**			**12**	**36,06**	**405,5**
				**32**			**20**	**31,38**	**369,07**
**21**	16198805	1,985			I	5'-3'		48	457
	18614106	2,255	PRSS7	3'-5'	I 16	5'-3'	ok	47	413
32 results	20923197	2,065				5'-3'		50	421
	21325923	2,475	NCAM2	5'-3'	I 1	5'-3'	no	29	389
	22466434	2,075				5'-3'		50	419
	23870733	2,375				5'-3'		28	439
	24104366	2,185				5'-3'		39	311
	25139835	2,09				5'-3'		20	416
	25645173	2,69				5'-3'		37	392
	33133929	2,335				5'-3'		44	427
	34095872	2,15	ITSN1	5'-3'	I 19	5'-3'	no	21	404

	38566460	2,05	C441964/KCN	5'-3'	I 4/ I 2	5'-3'	no	48	374
	39471226	2,155	DSCR2	3'-5'	E 6 I 5	5'-3'	ok	27	345
	44297144	2,38	TMEM1	5'-3'	I 4	5'-3'	no	26	418
	20361352	2,355				3'-5'		25	413
	21311227	2,19	NCAM2	5'-3'	I 1	3'-5'	ok	48	402
	21347316	1,72	NCAM2	5'-3'	I 1	3'-5'	ok	53	358
	21909639	1,955				3'-5'		53	413
	**22052894**	**2,57**				**3'-5'**		**39**	**430**
	22372281	1,865				3'-5'		50	461
	23597818	1,68				3'-5'		16	458
	24011561	2,385				3'-5'		42	437
	26129879	1,745				3'-5'		50	333
	26843730	2,39	CYYR1	3'-5'	I 2	3'-5'	no	33	332
	29264635	2,08	ZNF294	3'-5'	I 7 E 6 I 6	3'-5'	no	40	518
	29657226	1,675				3'-5'		47	289
	33732920	1,51				3'-5'		54	326
	34114333	2,34	ITSN1	5'-3'	I 24	3'-5'	ok	33	496
	37584554	1,675				3'-5'		24	539
	37744169	2,755	DYRK1A	5'-3'	I 1	3'-5'	ok	35	433
	38467512	0,99				3'-5'		53	606
	39076832	1,58				3'-5'		25	568
**Ø**				**8**			**12**	**38,56**	**419,91**
				**4**			**6**	**36,67**	**406,83**
				**12**			**6**	**39,7**	**427,75**
**22**	14736033	2,195			I	5'-3'		41	485
	15670554	2,645	XKR3	3'-5'	I 1	5'-3'	ok	38	393
24 results	15734747	1,875				5'-3'		16	419
	15749500	2,53				5'-3'		41	400
	17247960	1,415				5'-3'		18	531
	**20958063**	**2,48**				**5'-3'**		**32**	**360**
	24856137	2,02				5'-3'		27	500
	32575351	2,74	LARGE	3'-5'	I 2	5'-3'	ok	37	402
	38623209	1,885				5'-3'		16	417
	**40260955**	**2,14**	**POLR3H**	**3'-5'**	**I 2**	**5'-3'**	ok	**42**	**332**
	42505199	2,58	EFCAB6	3'-5'	I 3	5'-3'	ok	33	370
	45598637	1,73	TBC1D22A	5'-3'	I 4	5'-3'	no	26	548
	46696436	2,18				5'-3'		47	390
	15040134	2,445				3'-5'		43	415
	15400519	2,48				3'-5'		32	360

	17830919	1,215	UFD1L	3'-5'	I 5	3'-5'	no	17	257
	17937100	2,545				3'-5'		32	445
	27204747	2,125				3'-5'		47	439
	**29941068**	**2,49**	**LIMK2**	**5'-3'**	**I 1**	**3'-5'**	ok	**33**	**466**
	31919585	2,51				3'-5'		29	396
	32726330	1,965				3'-5'		17	411
	40179854	2,27				3'-5'		23	410
	47780335	1,3				3'-5'		17	274
	49159682	2,1	SAPS2	5'-3'	I 2	3'-5'	ok	22	434
**Ø**				**3**			**8**	**30,25**	**410,58**
				**5**			**2**	**31**	**400,25**
				**8**			**6**	**29,88**	**415,75**
**X**	4588098	2,03			I	5'-3'		39	280
	4910921	2,08				5'-3'		28	320
204 results	5366741	1,865				5'-3'		27	287
	5383731	1,85				5'-3'		50	354
	6077708	2,445	NLGN4X	3'-5'	I 2	5'-3'	ok	29	435
	7002058	1,24	HDHD1A	3'-5'	I 3	5'-3'	ok	49	596
	7307480	1,505				5'-3'		43	215
	10986009	2,505				5'-3'		38	365
	11157280	2,365	ARHGAP6	3'-5'	I 3	5'-3'	ok	29	451
	14654252	2,38	GLRA2	5'-3'	I 8	5'-3'	no	41	370
	17565670	1,505	NHS	5'-3'	I 1	5'-3'	no	27	215
	17871683	2,3				5'-3'		45	394
	18955972	2,785	GPR64	3'-5'	I 9 E 9 I 8	5'-3'	ok	36	401
	19401455	2,255	MAP3K15	3'-5'	I 1	5'-3'	ok	47	405
	22343326	1,955				5'-3'		18	395
	22917325	2,505				5'-3'		42	405
	22921869	1,845				5'-3'		16	425
	24211742	1,73				5'-3'		30	588
	24792401	2,505	POLA1	5'-3'	I 34	5'-3'	no	28	413
	26280930	1,82				5'-3'		21	338
	26325674	2,44				5'-3'		28	426
	27286861	2,775				5'-3'		37	409
	27611418	2,31				5'-3'		34	512
	27848059	2,305				5'-3'		44	433
	29059418	2,485	IL1RAPL1	5'-3'	I 2	5'-3'	no	35	487
	29332298	2,44	IL1RAPL1	5'-3'	I 5	5'-3'	no	27	416
	30023190	2,125				5'-3'		50	409

32384340	2,395	DMD	3'-5'	I 25	5'-3'	ok	40	363
35989996	2,46	CXorf59	5'-3'	I 1	5'-3'	no	43	412
37056924	2,315				5'-3'		24	411
37405508	2,5	LANCL3	5'-3'	I 3	5'-3'	no	31	374
38169538	2,745				5'-3'		36	425
38274738	2,4				5'-3'		36	494
40567988	2,435				5'-3'		30	371
42161810	2,41				5'-3'		27	396
42471124	2,28				5'-3'		46	418
43193847	2,21				5'-3'		24	432
43271194	1,77				5'-3'		17	450
44986548	2,105				5'-3'		21	423
48184424	1,875				5'-3'		22	339
53222377	2,21				5'-3'		29	482
53231413	2,305				5'-3'		24	413
56125373	2,765				5'-3'		36	421
65903906	2,055				5'-3'		29	305
65911327	2,82				5'-3'		34	408
66419150	2,19				5'-3'		24	436
66500324	1,805				5'-3'		33	603
66748710	2,48	AR	5'-3'	I 1	5'-3'	no	28	418
66964082	1,855				5'-3'		38	235
71231341	2,41				5'-3'		38	346
74548902	2,42	ZDHHC15	3'-5'	I 10	5'-3'	ok	42	388
79704930	1,92				5'-3'		47	338
79992747	2,555				5'-3'		39	433
80127980	2,245				5'-3'		24	393
80744007	2,155				5'-3'		49	413
81089894	2,025				5'-3'		20	389
81242754	2,72				5'-3'		38	410
83348188	1,51				5'-3'		44	226
83691460	2,66				5'-3'		37	432
84616419	1,955				5'-3'		35	593
85550097	1,905	DACH2	5'-3'	I 2	5'-3'	no	17	395
86285308	2,405				5'-3'		26	413
86805359	2,64	KLHL4	5'-3'	I 9	5'-3'	no	39	416
86880745	2,285				5'-3'		43	447
86958016	2,825				5'-3'		35	399
86971314	1,9				5'-3'		21	464
87290844	2,11				5'-3'		36	266

88638329	2,67				5'-3'		38	398
88989660	2,685				5'-3'		36	437
89297926	1,795				5'-3'		50	343
89711214	2,18				5'-3'		22	418
90674984	2,6				5'-3'		40	414
90758893	2,8				5'-3'		36	404
91022130	2,175	PCDH11X	5'-3'	I 2	5'-3'	no	47	429
91921352	2,075				5'-3'		51	409
92781982	2,49				5'-3'		42	416
94357507	1,695				5'-3'		20	495
94684893	2,075				5'-3'		51	409
95370261	1,935				5'-3'		43	301
97579840	2,61				5'-3'		39	396
97749374	1,905				5'-3'		23	483
99346759	2,06				5'-3'		23	452
102964781	2,13				5'-3'		48	390
104856115	2,105	IL1RAPL2	5'-3'	I 7	5'-3'	no	47	443
107255272	2,32	ATG4A	5'-3'	I 1	5'-3'	no	46	410
**107576770**	**2,13**	**COL4A5**	**5'-3'**	**I 1**	**5'-3'**	no	**49**	**418**
112702256	2,28				5'-3'		25	390
114147616	2,115	IL13RA2	3'-5'	I 8	5'-3'	ok	32	531
116968544	2,135	KLHL13	3'-5'	I 1	5'-3'	ok	23	437
117336879	2,715				5'-3'		38	411
117596667	1,915	DOCK11	5'-3'	I 13	5'-3'	no	50	451
119460199	2,24	LAMP2	3'-5'	I 7 E 7 I 6	5'-3'	ok	30	332
119842843	2,28				5'-3'		24	418
124263782	1,895				5'-3'		19	373
125821741	2,605				5'-3'		31	423
127331079	2,16				5'-3'		30	316
129201970	2,22	SUHW3	3'-5'	I 5	5'-3'	ok	32	308
131004908	2,17	RP6-213H19.[illegible]	5'-3'	I 1	5'-3'	no	26	358
131390623	1,89	MBNL3	3'-5'	I 1	5'-3'	ok	34	596
132213299	2,455				5'-3'		37	473
134066260	2,625				5'-3'		35	359
135672121	2,035	ARHGEF6	3'-5'	I 2	5'-3'	ok	47	361
136660381	2,6				5'-3'		37	374
136672365	2,615				5'-3'		40	411
136691497	2,18				5'-3'		47	428
141990441	2,44				5'-3'		39	456
142501349	2,19				5'-3'		39	506

144465787	2,52				5'-3'		41	420
145219146	2,595				5'-3'		30	415
146041299	1,39				5'-3'		46	222
147188203	2,01				5'-3'		50	386
150917939	2,54				5'-3'		41	402
151731524	2,42				5'-3'		38	470
153970538	2,535	BRCC3	5'-3'	I 5 E 6 I 6	5'-3'	no	37	361
154158422	2,565	CLIC2	3'-5'	E 6	5'-3'	ok	32	441
6518655	1,935				3'-5'		17	401
8103613	1,585				3'-5'		19	507
10789640	2,44				3'-5'		30	372
11104644	2,505	ARHGAP6	3'-5'	I 9	3'-5'	no	36	473
12428286	1,53	FRMPD4	5'-3'	I 2	3'-5'	ok	49	538
12841595	2,555	TLR8	5'-3'	I 2	3'-5'	ok	34	355
13160105	2,245				3'-5'		30	333
14751107	1,555				3'-5'		46	255
14994128	1,635				3'-5'		47	281
15711854	1,175	CA5B	5'-3'	I 8	3'-5'	ok	20	599
20870323	2,425				3'-5'		26	409
21364636	2,065	CNKSR2	5'-3'	I 3	3'-5'	ok	30	297
21445948	2,7	CNKSR2	5'-3'	I 9	3'-5'	ok	32	414
22226746	1,795				3'-5'		21	333
24135446	2,465	ZFX	5'-3'	E 6 I 6 E 7 I 7	3'-5'	ok	28	397
24620119	2,815				3'-5'		34	411
24760205	1,475	POLA1	5'-3'	I 32	3'-5'	ok	26	219
26613136	2,345				3'-5'		25	403
27203234	2,11				3'-5'		39	296
28594646	1,66	IL1RAPL1	5'-3'	I 1	3'-5'	ok	21	512
30783652	2,01	MAP3K7IP3	3'-5'	I 6	3'-5'	no	44	326
**31478206**	**2,86**	**DMD**	**3'-5'**	**I 55**	**3'-5'**	no	**35**	**406**
32377629	2,42	DMD	**3'-5'**	I 26	3'-5'	no	32	348
34381495	2,62				3'-5'		38	430
35170414	2,565				3'-5'		34	461
35648900	2,665				3'-5'		36	377
37054296	2,135				3'-5'		22	427
41332570	1,895	CASK	3'-5'	I 13	3'-5'	no	50	363
42703432	2,585				3'-5'		34	457
42926733	1,505				3'-5'		54	493
44772541	2,3	UTX	5'-3'	I 7	3'-5'	ok	38	324
44999074	2,02				3'-5'		42	308

45410277	1,995				3'-5'		22	455
46075013	1,73				3'-5'		24	290
46412208	2,06	SLC9A7	3'-5'	I 5	3'-5'	no	51	406
47538224	2,02				3'-5'		20	430
51610796	1,765	MAGED1	5'-3'	I 1	3'-5'	ok	19	347
53806283	1,99				3'-5'		45	486
55171752	1,64				3'-5'		28	586
57209240	2,245				3'-5'		27	455
63926455	1,995				3'-5'		27	505
64719237	1,24				3'-5'		18	252
66028984	1,75				3'-5'		18	354
68914660	1,925	EDA	5'-3'	I 1	3'-5'	ok	48	349
69945374	2,635	TEX11	3'-5'	I 9	3'-5'	no	39	417
75074279	1,905				3'-5'		45	503
77763548	2,23				3'-5'		44	448
78846828	2,535				3'-5'		38	447
79720964	2,62				3'-5'		40	410
81363970	2,465				3'-5'		42	397
81408995	1,96				3'-5'		50	442
82738449	2,245				3'-5'		45	435
82859065	2,2				3'-5'		38	514
84524616	2,29				3'-5'		35	292
85119624	2,41	CHM	3'-5'	I 4	3'-5'	no	28	386
86574297	2,555				3'-5'		35	473
86613650	2,68				3'-5'		32	400
89656302	2,22				3'-5'		47	398
89724206	1,885				3'-5'		46	321
92027378	2,1				3'-5'		22	434
92651819	2,025				3'-5'		20	429
92690008	2,21				3'-5'		48	406
93232226	2,25				3'-5'		29	344
93452634	2,08				3'-5'		49	390
96187940	1,74	DIAPH2	5'-3'	I 17	3'-5'	ok	16	372
98469530	2,505				3'-5'		33	463
99524999	2,765	PCDH19	3'-5'	I 2	3'-5'	no	33	411
99790073	2,735	SRPX2	5'-3'	I 2	3'-5'	ok	35	437
101054058	2,265	ZMAT1	3'-5'	I 1	3'-5'	no	44	377
109040396	2,2				3'-5'		30	324
111760993	2,12	LHFPL1	3'-5'	E 4	3'-5'	no	21	420
112748373	2,18				3'-5'		38	518

	112933601	2,315				3'-5'		24	407
	113271180	1,475				3'-5'		52	299
	115684215	2,325				3'-5'		25	399
	120969372	1,61				3'-5'		27	582
	121963495	2,275				3'-5'		29	349
	122978733	1,7	STAG2	3'-5'	I 1	3'-5'	no	24	284
	123511626	2,375	ODZ1	3'-5'	I 14	3'-5'	no	26	399
	123746573	1,805	ODZ1	3'-5'	I 3	3'-5'	no	50	345
	130156170	2,09				3'-5'		30	516
	131114752	2,635				3'-5'		31	401
	132159648	2,3				3'-5'		46	404
	139216756	2,16				3'-5'		46	376
	142608874	1,995				3'-5'		47	353
	142639759	2,745				3'-5'		37	403
	146920576	1,92				3'-5'		42	288
	150295637	2,01				3'-5'		18	406
	153858093	2,015	F8	3'-5'	I 6	3'-5'		18	407
**Ø**				**30**			**58**	**34,47**	**403,4**
				**29**			**31**	**34,69**	**402,69**
				**59**			**27**	**34,37**	**403,69**
**Y**	2857890	2,355			I	5'-3'		42	443
	2940326	2,675				5'-3'		34	439
56 results	3080590	2,69				5'-3'		38	402
	3430850	2,685				5'-3'		36	437
	4075373	2,21				5'-3'		22	412
	4784858	2,82				5'-3'		36	408
	5029955	2,175	LOC730420	5'-3'	I 2	5'-3'		47	429
	5692219	2,455				5'-3'		39	365
	5824374	2,075				5'-3'		51	409
	7521671	2,485				5'-3'		38	457
	7806566	2,11				5'-3'		35	256
	8094390	1,965				5'-3'		20	441
	9127015	2,105				5'-3'		45	355
	10126071	2,54				5'-3'		29	402
	14671927	1,975				5'-3'		19	429
	15220195	2,32	NLGN4Y	5'-3'	I 1	5'-3'	no	38	490
	15328014	2,135	NLGN4Y	5'-3'	I 2	5'-3'	no	48	427
	16807184	1,925				5'-3'		21	459
	17672089	2,255				5'-3'		43	365

	18551493	2,285				5'-3'		46	401
	19167324	2,23				5'-3'		43	458
	19488081	1,645				5'-3'		21	303
	19638623	2,345				5'-3'		37	495
	19969671	2,53				5'-3'		35	478
	20617431	2,295				5'-3'		42	363
	21912483	1,83				5'-3'		20	468
	22262525	2,41				5'-3'		44	406
	23879322	1,565				5'-3'		49	531
	24654370	2,285				5'-3'		46	401
	25494994	1,565				5'-3'		49	531
	26057979	0,96				5'-3'		16	602
	4020462	2,215				3'-5'		47	397
	4088336	1,87				3'-5'		46	318
	5930431	2,035				3'-5'		22	371
	6410963	2,535				3'-5'		29	401
	9069971	2,41				3'-5'		44	406
	10448344	1,895				3'-5'		20	455
	13779150	2,325				3'-5'		44	389
	13820601	2,24				3'-5'		23	416
	14612727	1,975				3'-5'		19	429
	15187843	2,66	NLGN4Y	5'-3'	I 1	3'-5'	ok	31	406
	16544203	1,945				3'-5'		53	403
	16705962	1,76				3'-5'		50	336
	17020082	1,93				3'-5'		21	458
	18596410	2,285				3'-5'		46	401
	19074240	1,645				3'-5'		21	303
	19395006	2,23	HSFY1/HSFY2	3'-5'	E 1	3'-5'	no	43	458
	21870942	1,65				3'-5'		54	464
	22204183	2,105				3'-5'		45	355
	22309814	2,55				3'-5'		29	404
	22831600	2,345				3'-5'		31	475
	23295332	2,425				3'-5'		38	469
	23651823	1,565				3'-5'		49	531
	24722612	0,96				3'-5'		16	602
	25285568	1,565				3'-5'		49	531
	26126212	2,285				3'-5'		46	401
**Ø**				**4**			**4**	**36,52**	**425,73**
				**1**			**3**	**41,4**	**442**
				**5**			**1**	**36,04**	**424,14**

Supplementary Material S2: Putative ncRNAs sequences.

NF1

CCAGTGTTATGTTTACCAAAAATGTTTGAGTGAGTCTTCTCTTTGTCTTTCTCTTTTTTAA
AAAATTCAGGCTCTGCTGGTTCTTCATCAGTTAGATAGCATTGATTTGTGGAATCCTGATG
CTCCTGTAGAAACATTTTGGGAGATTAGGTATATGTACTTTTATTTTTTAAATTCAACTTTT
AAATTTTATTTTGTATTTTTGTCTTGAAATATTAACTCTGTAGTACTTAGTACATTGTAAAA
CTTACACTTCCAAAGGTTTTATGGTTTTGTATTTTATTTGACTTCAAATTATTAGAATTTCTT
GTTTTAACTGTAAGAAAAGTATCACAGCAATTTAGAAAATAAATTTTAAGAATAGTGCTA
AATTTTG**TCACCCTAAC**ATAAGTACTGTTGTTTGG**TATATTA**CTTTTTTCAGATTTCAATG
TGGTTACTACTGTATTTTTA**ATAGATTTTCATAGTTATAAGCCT**A

PARK2

CCAGCATGATATTTGGCATCTCACTGGGATGCAAAAGGGAACATTATTTGCTGCTGCGT
GCCATTAGTGCCAGAAACATTTTTTCATAAAATGAAGAGTGCAGCTCCACTAATCTATTAG
TCCTAAGCCATTTTAGATCATACCATTGATTAACTAAAATATTTTTTTTCAAATGACGGCT
GATGACAGAGCA**CTACCTTAAAAGTCAA**AGAAC**TATAAA**TTAAACATGAATCCAGTTAC
CATGTTCTGTCAAAACAAGAAATACATGCGTGTGTTTGTG**TCCTTTATATGGGCCAACAT
A**

TNC

ACATATTTTCTATTTGGTCATTTAAGGAAATGTTTACTGATCCCTTCCAGTCAACCTTAC
TAAATTTTTATACTTACTTTCCAG**TCACCTTAAC**CTAACAAGGTGGATATTATCATACTTG
TGTA**TATA**GAGATTGAAGCAGAAAAAAAAAAATCATCTAGTAA**GTTCCCCTCCAAAAACA
TAATG**

Runx2

TCGGGTCATATAAGGCTTGCCTTGTCACACCTTACCCTTGACTAGAATCAATGAGAAAT
AACTTTGGCCACCTCTGTGTACTGAATAATCTTTTAGAAACATGTAGGATGAAGTGAGATA
ATGTATAAAATCACCTGGGGATAGGAGTGCTACTTAAATACAGATTATTATATGATGAGG
CCATATGTATCAATGTAAGTATGGTAGGAAAGGAGGTGTCTTCTGCACTGTAACATCACTA
GACTGGTTTCTGAGACTAGTGCTGACTTTTATAATTCAACTGAATATGATTCAAACTCA**TT
ACCATAAC**ATACACCTGTTAGTCAATTACATGGCAATAACTTCTCATTCAATTACAAACT**T
ATA**TGTGCTCTTGTTGTTCCATTACAATATTTAAGGCTACCCAATGTTAAGGAAATATTTC
CTGAATCATTGTA**TTGTACCTCTGAACTTGAAGACG**

NDUFS4
AGTGTCAAGGGAAACCATGCTCATGACTTCTCTTTTTATTTGACATTTAAAAGGGAAATG
AAAAAACTAAGGAGATAACAGGTAGGCTTTTATCTTCAGAATAGTCCCTGAATTGATATA
CAAATAATCAACTGTATCTCAAATACAATTGATAACAAATTGTATTTGATAATTGATATAC
AAATCAAATAACTGTCAAAAGCTAATATACTTTTAGGCTGCATTAATATAAAATACAATTT
GATAAGTGATACATAAATCATCTTTTGTGTAGGTATGTGGCAGTTTT**ACCCTAAC**TTCCTA
TTTCTAGATACTACTGAAAGGCAAGCATTAATGCCCATAAACG**TATAA**CCTTTGTCATATT

TTACTATTAACACAGAATCAGCTGTATTAACAAAAACTCATTTCCCCCTATACAGCATA**TGAGTAAGGCACTGGGCTAGA**

In red: PSE
In blue: TATA box
The primers are underlined

© 2012 by the authors; licensee MDPI, Basel, Switzerland. This article is an open access article distributed under the terms and conditions of the Creative Commons Attribution license (http://creativecommons.org/licenses/by/3.0/).
